# iMicrobe: Tools and data-driven discovery platform for the microbiome sciences

**DOI:** 10.1093/gigascience/giz083

**Published:** 2019-07-09

**Authors:** Ken Youens-Clark, Matt Bomhoff, Alise J Ponsero, Elisha M Wood-Charlson, Joshua Lynch, Illyoung Choi, John H Hartman, Bonnie L Hurwitz

**Affiliations:** 1 Department of Biosystems Engineering, University of Arizona, 1177 E. 4th St, Shantz Building, Room 403, Tucson, AZ, USA 85721-0038; 2 Environmental Genomics and Systems Biology Division, E.O. Lawrence Berkeley National Laboratory, Berkeley, CA, USA; 3 Department of Computer Science, University of Arizona, Tucson, AZ, USA; 4 BIO5 Institute, University of Arizona, Tucson, AZ, USA

**Keywords:** cyberinfrastructure, cloud computing, bioinformatics, metagenomics

## Abstract

**Background:**

Scientists have amassed a wealth of microbiome datasets, making it possible to study microbes in biotic and abiotic systems on a population or planetary scale; however, this potential has not been fully realized given that the tools, datasets, and computation are available in diverse repositories and locations. To address this challenge, we developed iMicrobe.us, a community-driven microbiome data marketplace and tool exchange for users to integrate their own data and tools with those from the broader community.

**Findings:**

The iMicrobe platform brings together analysis tools and microbiome datasets by leveraging National Science Foundation–supported cyberinfrastructure and computing resources from CyVerse, Agave, and XSEDE. The primary purpose of iMicrobe is to provide users with a freely available, web-based platform to (1) maintain and share project data, metadata, and analysis products, (2) search for related public datasets, and (3) use and publish bioinformatics tools that run on highly scalable computing resources. Analysis tools are implemented in containers that encapsulate complex software dependencies and run on freely available XSEDE resources via the Agave API, which can retrieve datasets from the CyVerse Data Store or any web-accessible location (e.g., FTP, HTTP).

**Conclusions:**

iMicrobe promotes data integration, sharing, and community-driven tool development by making open source data and tools accessible to the research community in a web-based platform.

## Introduction

iMicrobe is a platform that connects researchers’ own data to published, curated, microbial metagenomic datasets and high-performance computing (HPC) methods for their analysis [1]. In the past decade, the cost of sequencing has decreased at a rate far outpacing Moore's law, leading to a rapid increase in the number and size of biological datasets [2]. Researchers now have access to an unprecedented scale and variety of data ranging from large-scale ‘omics data to streaming data from sensors. Biologists increasingly need the power and storage of HPC clusters to perform analyses; however, most biologists have limited or no access to these resources and often have to run analyses on their own personal computers.

To address the growing need for HPC in computational biology, the National Science Foundation has funded shared cyberinfrastructure resources such as the Extreme Science and Engineering Discovery Environment (XSEDE) [3] and Stampede2, an 18-petaflop supercomputer at the Texas Advanced Computing Center (TACC) at the University of Texas at Austin. Developers at TACC have created Agave [4], a representational state transfer (REST) [5] API, to interact with Stampede2’s resources including creating and editing applications (apps), scheduling, and monitoring jobs, and viewing and retrieving the results of analysis jobs. The iMicrobe website makes use of the Agave API [6] to create a web-based portal to the CyVerse [7] Data Store [8] and HPC resources such as the Stampede2 HPC via a free CyVerse account.

**Figure 1:**
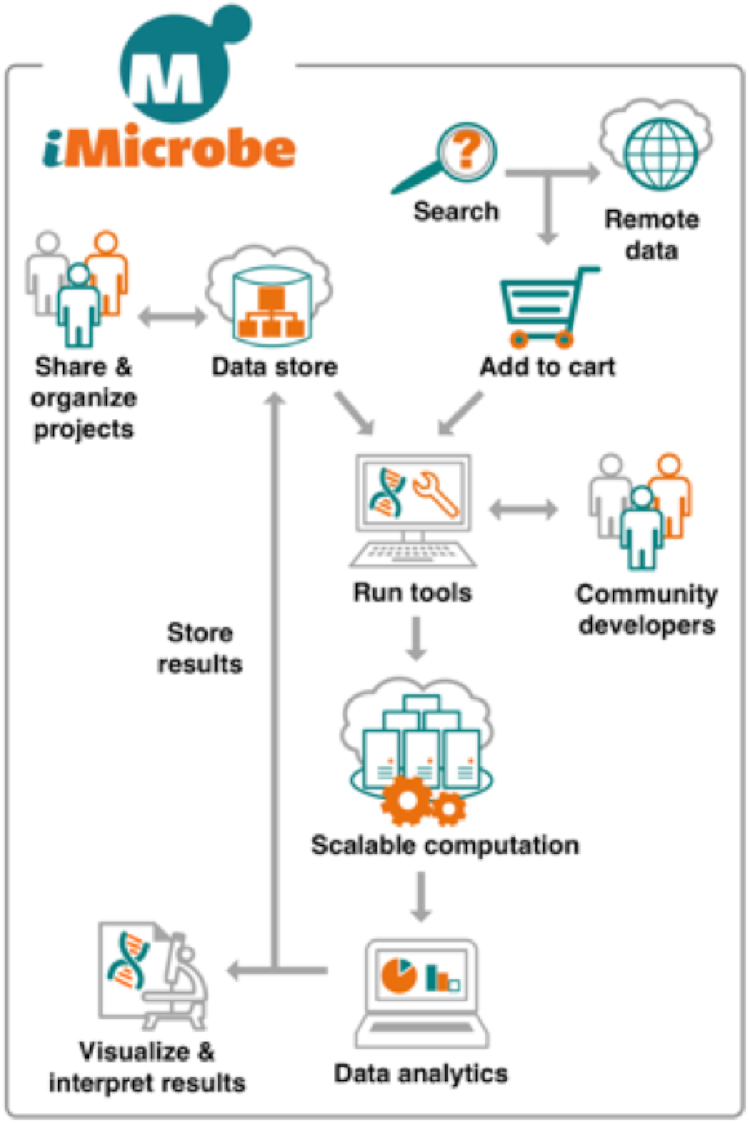
iMicrobe's architecture allows for the integration of datasets hosted by iMicrobe that can be placed into the data cart, those private to the user, and others publicly accessible on the Internet. Analyses created by iMicrobe or other developers run on Stampede2, and the results go into the user's home directory in the CyVerse Data Store.

iMicrobe users can use their web browser to search public metagenomics datasets like Cyberinfrastructure for Advanced Marine Microbial Ecology Research and Analysis (CAMERA) [9], save data to a cart, upload their own personal datasets, and run >30 analysis tools on both private and public datasets using free compute on Stampede2 (Fig. 1 ). All data in iMicrobe are also available via FTP [10] or from the CyVerse Data Store via iRODS [11] (command line) or the CyVerse Data Commons [12] (web browser). The Agave API also allows for direct command-line access to data and pipelines via the CyVerse Software Development Kit (SDK) [13]. Users can log in directly to Stampede2 and use iMicrobe's analysis tools in their Singularity [14] containers or build containers from source to use on their own computing resources. Finally, developers can use the Agave API to create novel tools that can be integrated into iMicrobe.

**Figure 2:**
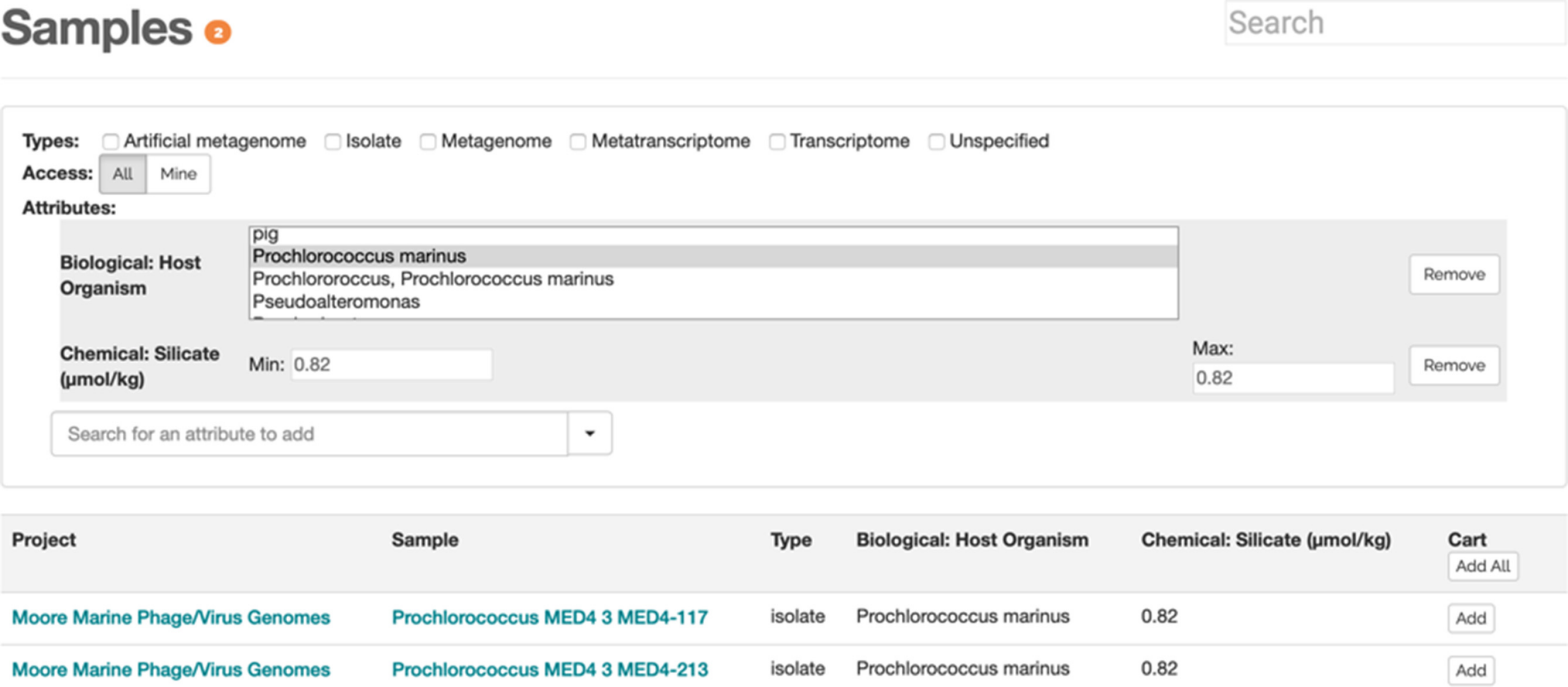
The sample metadata search allows users to search over both text and numeric values in any combination. Each additional search attribute updates the discovered samples immediately and restricts the next attributes the user can use to those found in the sample subset. Valid search values are displayed as multi-select boxes for strings and minimum/maximum values for numbers.

**Figure 3:**
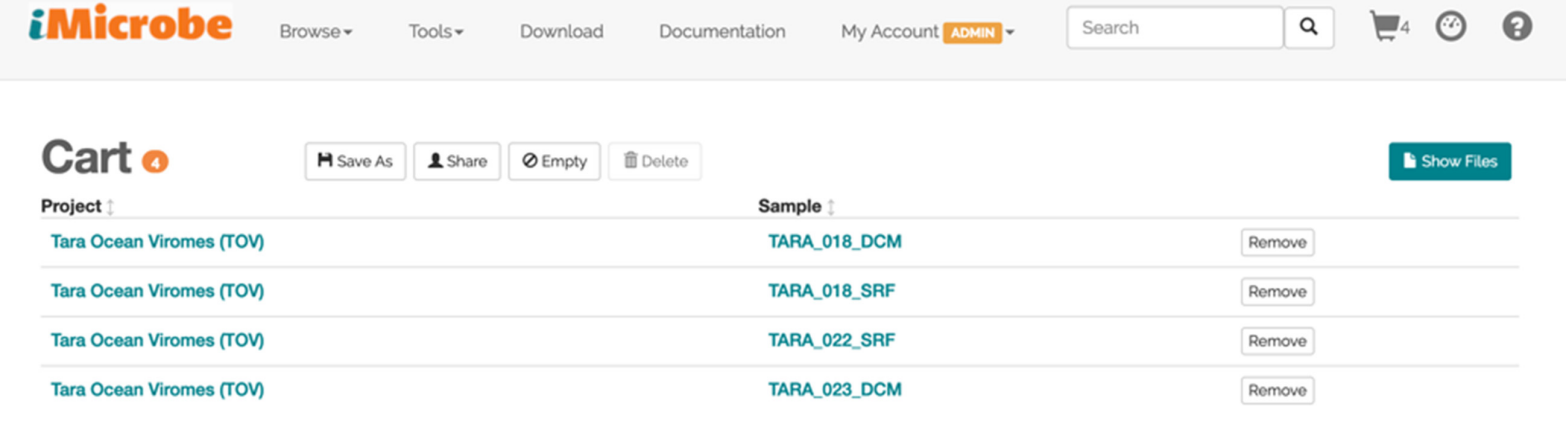
The data cart holds any number of samples selected by the user. Using the cart view, users can select the associated data files or analysis products to download. Cart contents can also be used as input values for apps.

## Findings

### iMicrobe leverages CyVerse cyberinfrastructure and XSEDE supercomputing resources

#### Security and trustworthiness

iMicrobe leverages the CyVerse cyberinfrastructure to provide services to users including the OAuth2 authentication system [15] for secure single sign-on between iMicrobe and all CyVerse services as well as the CyVerse Data Store for storing, sharing, and distributing large amounts of data and analyses. Users can also access HPC systems such as XSEDE's Stampede2 cluster to execute analyses that originate from iMicrobe apps.

#### Usability and data storage

iMicrobe uses the CyVerse Data Store for datasets and analysis results. CyVerse Data Store offers solutions to many contemporary data storage needs in the age of large, distributed, digital data. CyVerse's cloud-based data storage is optimized for large data, is free to most scientific researchers, is accessible through multiple interfaces, and leaves access control in the hands of the data owners. The Data Store provides research scientists, research groups, and research organizations with private, shared, or public storage allocations primarily for use within the CyVerse cyberinfrastructure. The CyVerse Data Commons houses public data within the Data Store for use by the research community either within or outside CyVerse cyberinfrastructure. The Data Store offers reliable, secure storage for datasets of any size that are actively being used for both research and/or education purposes. Data and metadata in the Data Store are stored in a high-performance storage resource that has built-in redundancy and is continuously monitored for security and failure. The Data Store is synchronously backed up at both the University of Arizona in Tucson, Arizona, and at the TACC in Austin, Texas. All users initially get 100 GB of storage but can request additional allocations through CyVerse [16]. Private user data is stored in a user's home folder (“/iplant/home/$user,” where $user is a CyVerse username) and will be accessible through a view in iMicrobe where files can be uploaded, deleted, shared, or modified. Users can share data that they own with other registered CyVerse users. Project leaders can request larger allocations for collaborative projects. Project leaders can also request a Community Data folder that will be made public [17]. Community Data folders are housed in the “iplant/homes/shared” directory and are visible via iMicrobe under "Community Data.” Data policies associated with iMicrobe are in sync with CyVerse policies. Additionally, iMicrobe is a data broker for the European Nucleotide Archive (ENA). Users can upload sequence files, enter project/sample metadata, and publish to ENA, all within the iMicrobe interface. iMicrobe validates the data/metadata and suggests additions or corrections but does not enforce any standards. This feature is available on request. A future version of the site will include this and conform to community standards.

#### Provenance and reproducibility

iMicrobe data and analysis provenance: primary data, derived files, and analyses are tracked in CyVerse by keeping all files in the analysis directory along with data products and a log file to maintain information about the job and parameters that were run. CyVerse also maintains a job history to allow researchers to track and reproduce experiments. In iMicrobe, data provenance is imperative given that data are derived from diverse data stores that have varied levels of curation and versioning.

### General search

The upper right corner of every page on iMicrobe has a search box that will perform a simple query over text in our databases related to projects, investigators, samples, taxonomy, and proteins. For instance, a search for “obese” finds 28 hits including a publication, 2 projects, and the 25 samples from those 2 projects.

### Metadata search

The samples in iMicrobe can be searched by their metadata, or “data about the data.” Samples are described in detail by >200 attributes such as type (artificial metagenome, isolate, metagenome, metatranscriptome, transcriptome), biome (sewage, soil, deep chlorophyll max, acid mine drainage), depth, dissolved oxygen, latitude/longitude, salinity, host organism, chlorophyll, and more (Fig. 2). The values for these can be numeric or character values such as a measurement of depth in meters or “Synechococcus” for the host organism. In total, there are >140,000 descriptors for our samples, and users can specify an unlimited number of terms using the sample metadata search tool [18]. Datasets included in the metadata search were inherited from the CAMERA project and were not thoroughly curated by CAMERA or the original data providers and do not conform to current community standards. As such, discrepancies may exist and users can contact original data providers and the iMicrobe developers via the website to address problems.

Each time a user selects a new attribute such as “phosphate,” the interface determines whether the attribute is character or numeric data. For character data, the user is presented with a multi-select list if the number of choices is reasonable to aid in selecting the correct strings. For numbers, minimum and maximum fields are shown with placeholders indicating the current min/max values from the database for the current subset of records shown. For example, if “phosphate” is selected as the first attribute, the min/max values are 0.01/3070, respectively, but if the user first selects “Longhurst Province” of “ARAB” (NW Arabian Upwelling Province), then the “phosphate” values will be only for those samples found in that province, which range from 36 to 50. In this way, users may quickly winnow sample searches to those matching their exact criteria and place the results into the cart.

From the sample details page (e.g., [19]), users can view a sample's location on Google Maps (if applicable), add the sample to the cart, view and download the associated data products, and examine the sample's attributes, predicted proteins, and predicted taxonomic classifications.

### Data cart

Throughout the iMicrobe site, users can add an unlimited number of samples of interest to the data cart (Fig. 3). Samples can be easily removed, or the cart can be cleared entirely. Using the cart, users can download the associated data products. Users can filter data products (files) by their associated types (e.g., reads, gene calls, predicted proteins, taxonomy classifications). Users can download data either from the CyVerse Data Store or the iMicrobe FTP site [10]. Contents of the cart can also be used as the input to several of iMicrobe's apps. Carts can be saved and shared with other users.

### Apps

**Figure 4:**
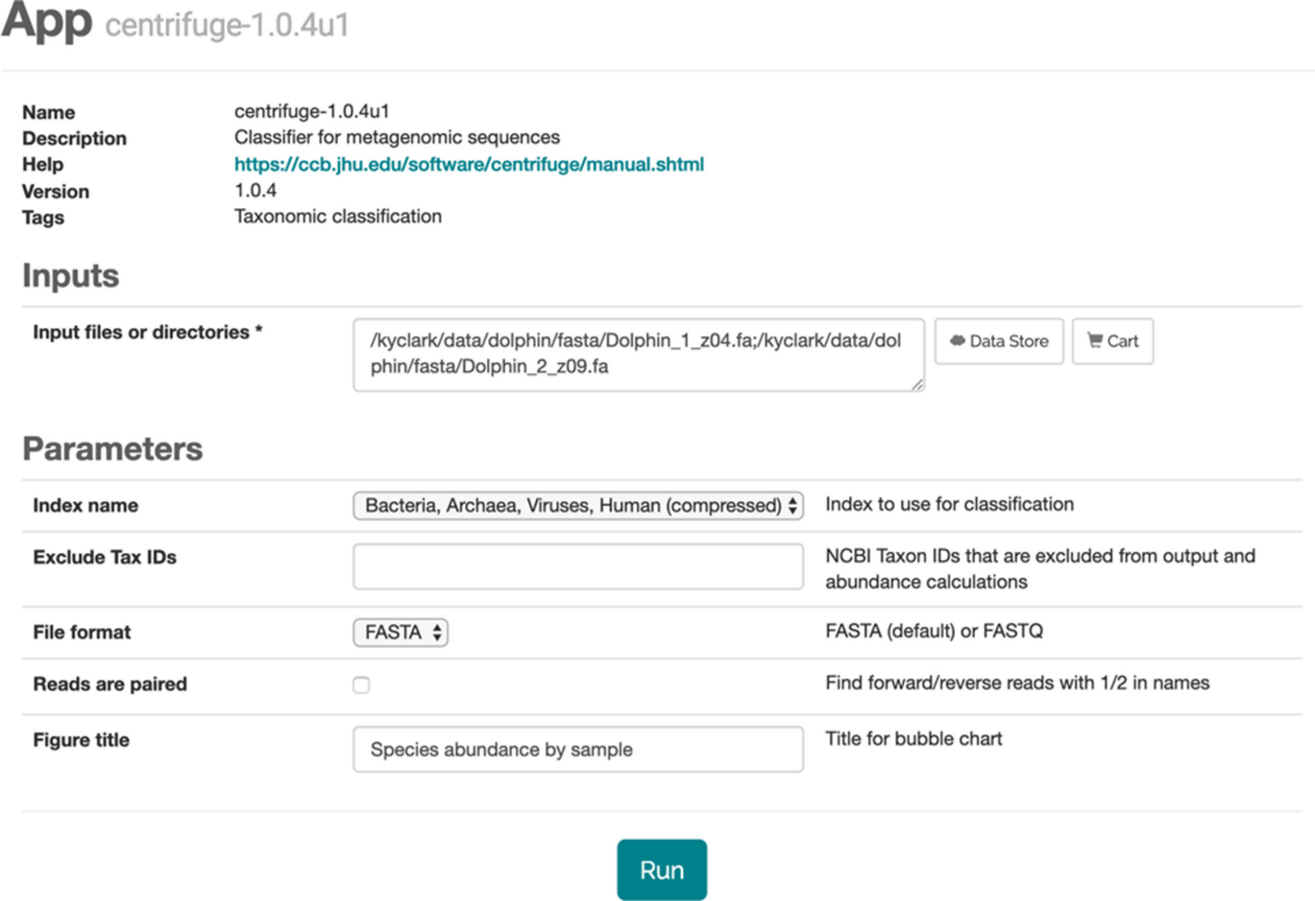
The app launch interface allows users to select input files and set parameters for the app. The input files may come from the user's own Data Store, any publicly available data in the Data Store such as files associated with their data cart or iMicrobe sample files, or any other file available over FTP or HTTP. The Agave API will copy the input files to the compute node when the job is run.

iMicrobe currently hosts >30 self-contained apps (Table 1) or analysis pipelines that take some input files and program parameters (Fig. 4), run to completion, and deposit the results into the user's CyVerse Data Store home directory. Apps allow users to easily do quality control (QC) (Trim Galore, Trimmomatic), predict proteins (UProc) and genes (Prodigal, MetaGeneAnnotator, FragGeneScan), assemble contigs (Megahit, SOAPdenovo), assign taxonomy (Centrifuge), and cluster genomes (Mash, Libra, Fizkin) (Table 1). The current selection of apps provide additional read-based analytics not currently available on other platforms and specialized pipelines from collaborators. Future development will also include community-defined pipelines that are made available by major data repositories, such as ENA, to allow users to harmonize their data with other public data sources in a consistent manner. Currently users can find tools by using standardized tags or app names and descriptions. As the app lists grows, we plan to offer additional ways to organize and search for apps, including by app versions.

**Table 1:** Current list of available apps in iMicrobe

App Name	Purpose
16s_cluster-0.0.1u2	Cluster 16S sequences [20]
c-microbial-map-0.0.1u1	Visualize geographic distribution of 16S sequences in the ocean [21]
centrifuge-1.0.4u1	Short-read taxonomic classification [22]
centrifuge-bubble-0.0.5u1	Visualization of Centrifuge analysis [23]
ClusterGenomes-1.1.3u2	Clusters genomes based on all-versus-all alignments
DIAMOND-0.9.10u1	Fast read alignment of DNA or proteins [24]
fizkin-0.0.3u1	Pairwise sample comparison via *k-*mers [25]
FragGeneScan-1.30.0u1	Short-read ORF prediction [26]
graftm-0.11.1u3	Rapid community profiles from metagenomes [27]
imicrobe-demultiplexer-0.0.1u1	Demultiplexing pipeline for single and paired-end data [28]
imicrobe-megahit-0.0.2u1	Metagenomics read assembler [29]
imicrobe-prokka-0.0.2u1	Prokaryotic genome annotation [30]
imicrobe-soapdenovo2–0.0.3u1	Short-read assembler [31]
libra-1.0	Pairwise sample comparison via *k-*mers [32]
MArVD-1.0.0u1	Metagenomic Archaeal Virus Detector [33]
mash-all-vs-all-0.0.5u1	Pairwise sample comparison via Mash [34]
MetaGeneAnnotator-1.1.0u1	Prokaryotic and phage gene prediction [35]
ohana-blast-0.0.9u2	BLAST search to Ohana gene catalog [36]
prodigal-2.6.3u3	Gene prediction [37]
Prokka-1.12.0u2	Prokaryotic genome annotation [30]
puma-0.3.0u1	Annotation of human papillomavirus genomes [38]
Read2RefMapper-1.1.0u2	Filtering coverage of BAM files to a reference dataset [39]
sra-fastq-dump-0.0.1u1	Save sequences from SRA in CyVerse Data Store [40]
trim-galore-0.4.5u1	QC tool for trimming reads [41]
Trimmomatic-0.36.0u2	QC tool for trimming reads [42]
uproc_dna-1.2.0u3	Protein sequence classification [43]
vContact-0.1.60u2	Viral Contig Automatic Cluster Taxonomy [44]
vContact_PCs-0.1.60u2	Viral Contig Automatic Cluster Taxonomy [44]
WIsH-Build-1.0.0u2	Identify bacterial hosts from metagenomic data [45]
WIsH-Predict-1.0.0u2	Identify bacterial hosts from metagenomic data [45]

To view and run apps, the user must first create an account with CyVerse [46]. Accounts are free to create and allow iMicrobe to connect in a user's data in CyVerse including novel datasets they may wish to analyze with an app or the results of running an analysis tool. Currently workflows are implemented inside an app. Future development plans include chaining apps together to create ad hoc workflows. To ensure the long-term availability of the apps, we provide recipes to build the Singularity containers in the GitHub repository for each tool as well as the containers themselves on the iMicrobe FTP site and direct access to the containers in a public directory on the Stampede2 cluster.

Apps can allow for new tools to be more quickly disseminated to the community. Often the discovery of a newly published bioinformatics tool leads to the frustration of downloading and compiling source code, resolving dependencies, and fighting with conflicting versions of tools (e.g., gcc or Python/conda). When tools are released as self-contained packages, others can more quickly and easily test the tools on their own datasets. If community developers release tools as containers, they can more easily be made available as apps through Agave and iMicrobe.

### Making data and tools findable, accessible, interoperable, and reusable (FAIR)

iMicrobe is pursuing FAIR [47] (findable, accessible, interoperable, and reusable) principles as they relate to both data and computation. We strive to make data findable via searches; accessible via the CyVerse Data Store, iRODS or FTP; interoperable via common file formats; and reusable via open access. Likewise, we aim to make compute findable via our website, accessible via the Agave API, interoperable via Singularity containers, and reusable via open access. Just as common file formats like FASTA or GFF make data exchange simple, containers like Docker [48] and Singularity allow computational methods to be run on any system and promote reuse.

### Reusable

#### Making data reusable via the dynamic metadata search in iMicrobe

An acute need in the microbiome community is to discover and integrate data in disparate data repositories to facilitate analyses. Specifically, primary and associated contextual metadata, as well as other data products (and their provenance), exist in diverse microbiome data repositories. To make data more discoverable, iMicrobe provides a dynamic search to find datasets based on structured and curated metadata. Data can be added to a shopping cart and analyzed using a variety of tools.

#### Making tools reusable by creating containers

As for computing resources, users can find iMicrobe apps on the iMicrobe website [49]. Most iMicrobe apps use Singularity containers that can be accessed directly from the Stampede2 file system under a shared iMicrobe directory. The recipes to build iMicrobe's Singularity containers are stored in GitHub repositories (see Table 1), making it possible for users to build and use containers locally on their own datasets.

### Accessible

#### Analyzing remote web-accessible datasets in iMicrobe

Because much ‘omics data lives in diverse repositories globally, moving the data to a central location for processing can be problematic and inefficient. Still, scientists need to bring together diverse datasets to enable population-level or planetary-scale analyses that drive new knowledge and discovery. iMicrobe delivers a virtual framework for connecting web-accessible remote microbiome ‘omics data with private user data using the Agave API. Reasonably sized datasets that are available via a web-link (FTP or HTTP) are accessible and computable in iMicrobe if the analysis can be completed within a standard 24–48-hour window. iMicrobe provides ready access to the CAMERA data collection including reads, peptides, CDS, contigs, assemblies, and annotations, as well as related projects derived from the sample and environmental data for ≥120 microbiome projects, representing 1 TB of data. These data are hosted in the CyVerse Data Store (“/iplant/shared/imicrobe”) and are integrated into iMicrobe under "Community Data.” A user can analyze CAMERA data alongside their own personal datasets and also any other dataset that the Agave API can retrieve from a public web address.

### Interoperable

#### Containerizing tools and pipelines for making analyses interoperable on diverse compute platforms

Microbiome science methods and bioinformatics code are constantly evolving and can be cumbersome to install [50]. Moreover, users may not have access to the computing resources required to run the tool on their data. To this end, iMicrobe converts tools into Singularity containers, packaged virtual machines that encapsulate the operating system, dependencies, and tool's code to ensure reproducibility and allow the code to run on any computational architecture including the Stampede2 HPC. Community developers can contribute tools by releasing containers to Biocontainers or Docker Hub. Currently, most iMicrobe apps are deployed at TACC Stampede2 using the Agave API; however, the containerized tools can theoretically be run on any computer resource including cloud resources such as Amazon Web Services or Google Cloud Platform. The iMicrobe platform automatically creates a user interface to launch a container by using a JSON (JavaScript Object Notation) description of the app that encodes its inputs and parameters. The JSON also specifies hardware requirements (CPU and memory) to run the tool, e.g., on the default queue or a high-memory node at Stampede2. iMicrobe streamlines community-driven tool development and accessibility to a variety of tools in a simple web-based platform.

#### Running Analyses through the iMicrobe web-based platform

To run an analysis, users select an app from the apps listing; select data “inputs” from their own CyVerse Data Store, publicly available data in their shopping cart, or some publicly accessible URL; select parameters; and launch the tool with a click of the “Run” button. Users can track the status of their jobs directly on the site and view results and interactive data visualizations. As with all files, users can share analysis results with collaborators. Provenance of primary data-derived files, and analyses are tracked in CyVerse by keeping all files in the analysis directory, along with data products and a log file record about the job, including data sources, app versioning, and the parameters selected for that run. CyVerse also maintains the job history to allow researchers to track and reproduce other researcher's experiments.

### Reusable

#### Virtual communities, protocols, and documentation for iMicrobe

Given the experimental nature of methods in microbiome research, iMicrobe fosters discussions about both molecular and computational protocols with an eye towards improving methods. iMicrobe partners with protocols.io [51], a method-centered collaborative platform, to provide guides and sample datasets for popular use cases in microbiome research. These methods are available through the iMicrobe virtual community [52]. Through protocols.io, scientists can also create and share their own protocols and/or groups in the microbiome sciences as well as engage in discussions. Users can also access documentation on using the iMicrobe website and protocols through the iMicrobe Gitbook [53]. Users are also encouraged to submit feedback directly to the site via the Feedback button. All bug fixes and feature suggestions are discussed within the development team and prioritized.

## Comparison to Web-based Metagenomics Platforms

There are many tools for metagenomic analysis, many of which are native desktop applications for Windows, Apple, or Linux operating systems. Because iMicrobe is a web application focused on connecting remotely hosted datasets to large compute capacity, we have chosen several similar systems to which we compare it. See Tables 2 and 3 for a comparison of metagenomic platforms’ cyberinfrastructure capabilities and app capabilities, respectively.

**Table 2:** Comparison of metagenomic platforms’ cyberinfrastructure capabilities

Capability	KBase	MGnify	MG-RAST	IMG/M	QIITA	iMicrobe
Create apps	✓					✓
Run apps at will	✓		✓		✓	✓
Upload private data	✓		✓	✓*	✓	✓
Share private data						✓
Search public data	✓	✓		✓	✓	✓

^*^For users of JGI sequencing services.

**Table 3:** Comparison of metagenomics platforms’ app capabilities

Category	Capability	KBase	MGnify	MG-RAST	IMG/M	QIITA	iMicrobe
General	QC	✓	✓	✓	✓	✓	✓
Genomics	Assembly	✓					✓
	Gene calling	✓					✓
	Gene annotation	✓					✓
	Metabolic modeling	✓					
	Sequence analysis	✓					✓
	Comparative genomics	✓					
Metagenomics (assembly-based analysis)	Assembly	✓			✓		✓
	Gene calling			✓	✓		✓
	Gene annotation			✓	✓		✓
	Taxonomic classification of contigs			✓	✓		✓
	Protein clustering			✓			✓
	Read mapping to contigs or other reference	✓					
Metagenomics (read-based analysis)	Read taxonomic classification	✓	✓	✓			✓
	Read ORF prediction		✓	✓			✓
	Read functional annotation		✓	✓			✓
	Read clustering						✓
Amplicon	Operational taxonomic units and taxonomic lineage		✓	✓	✓	✓	✓

### KBase

The Department of Energy's (DOE) Systems Biology Knowledgebase [54] is “an open-access bioinformatics software and data platform for analyzing plants, microbes, and their communities.” KBase offers several dozen apps, which can be organized into workflows called “narratives.” To create a novel app, users must apply for a KBase developer account and install the KBase SDK and dependencies (Java 1.7, Python 2.7, NodeJS, Bower, Docker), and work with the KBase staff to integrate the app. Apps run on computer resources at Lawrence Berkeley National Laboratory or Argonne National Laboratory, with plans to expand to other DOE HPC or cloud resources. In contrast, developers who wish to create a CyVerse or iMicrobe app are simply required to put that app into a Singularity container and describe the inputs and parameters using a JSON document. As described below, web-based interfaces can be used to create the necessary JSON app definitions.

### MGnify

MGnify [55] is metagenomics platform from the European Bioinformatics Institute (EBI). Users can publicly archive their data and receive a permanent accession, which can be used to retrieve sequences and metadata via EBI's European Nucleotide Archive (ENA). After releasing data to ENA, MGnify integrates metagenomic datasets and may run 1 or more versions of their standard pipeline on datasets [56]. Metadata searches in MGnify include temperature, depth, biome, sequencing method, and a few other fields. In contrast, all metadata fields are available in iMicrobe via the sample/metadata search. Users are not able to run MGnify analysis pipelines directly but may submit a request to analyze private or public datasets. In contrast, iMicrobe users can run apps (which include analysis pipelines) directly from the integrated app and job submission interface as described above.

### MG-RAST

MG-RAST [57], or the Metagenomic Rapid Annotations using Subsystems Technology server, makes it possible for users to upload raw metagenomic sequence data in FASTQ or FASTA format. Assessments of sequence quality and annotation with respect to multiple reference databases are performed automatically with minimal input from the user. Post-annotation analysis and visualization are also possible, directly through the web interface, or with tools like matR (metagenomic analysis tools for R) that use the MG-RAST API [58] to download. Similar to MGnify, a single comprehensive analysis pipeline is applied to all user datasets. By contrast, iMicrobe users can upload any data type relevant to any app and run them in a modular fashion and adjust the parameters to be more specific to their data and research question.

### IMG/M

The Integrated Microbial Genomes and Microbiomes (IMG/M) [59] is a service that supports “the annotation, analysis, and distribution of microbial genome and microbiome datasets sequenced at DOE's Joint Genome Institute (JGI).” Users can use IMG/M for “annotation, analysis, and distribution of their own genome and microbiome datasets” but cannot create and distribute novel tools for community access. Like MGnify and MG-RAST, IMG/M provides uses with a single comprehensive pipeline for analysis. As described previously, iMicrobe provides users with apps that are self-contained and can be run in any order, with user-defined parameters.

### QIITA

QIITA [60] allows users to upload and analyze maker gene datasets (16S ribosomal RNA [rRNA], Internal Transcribed Spacer (ITS), and 18S rRNA), metagenomic, and metabolomic datasets using QIIME2 and Global Natural Product Social Molecular Networking (GNPS) tools. Using these tools, users can examine the taxonomic composition of marker gene and whole-genome shotgun (WGS) metagenomic datasets, and perform automated molecular network analysis and crowdsourced tandem mass spectroscopy spectrum curation for metabolomic datasets. However, QIIME2 does not currently support upstream analyses for WGS datasets or analyses beyond taxonomic annotation and analysis. iMicrobe currently hosts a 16S rRNA pipeline and clustering tool [20], in addition to many other general purpose tools for QC, trimming, read assembly, pairwise sequence alignment, and gene functional annotation, among others.

## Methods

### Architecture

**Figure 5:**
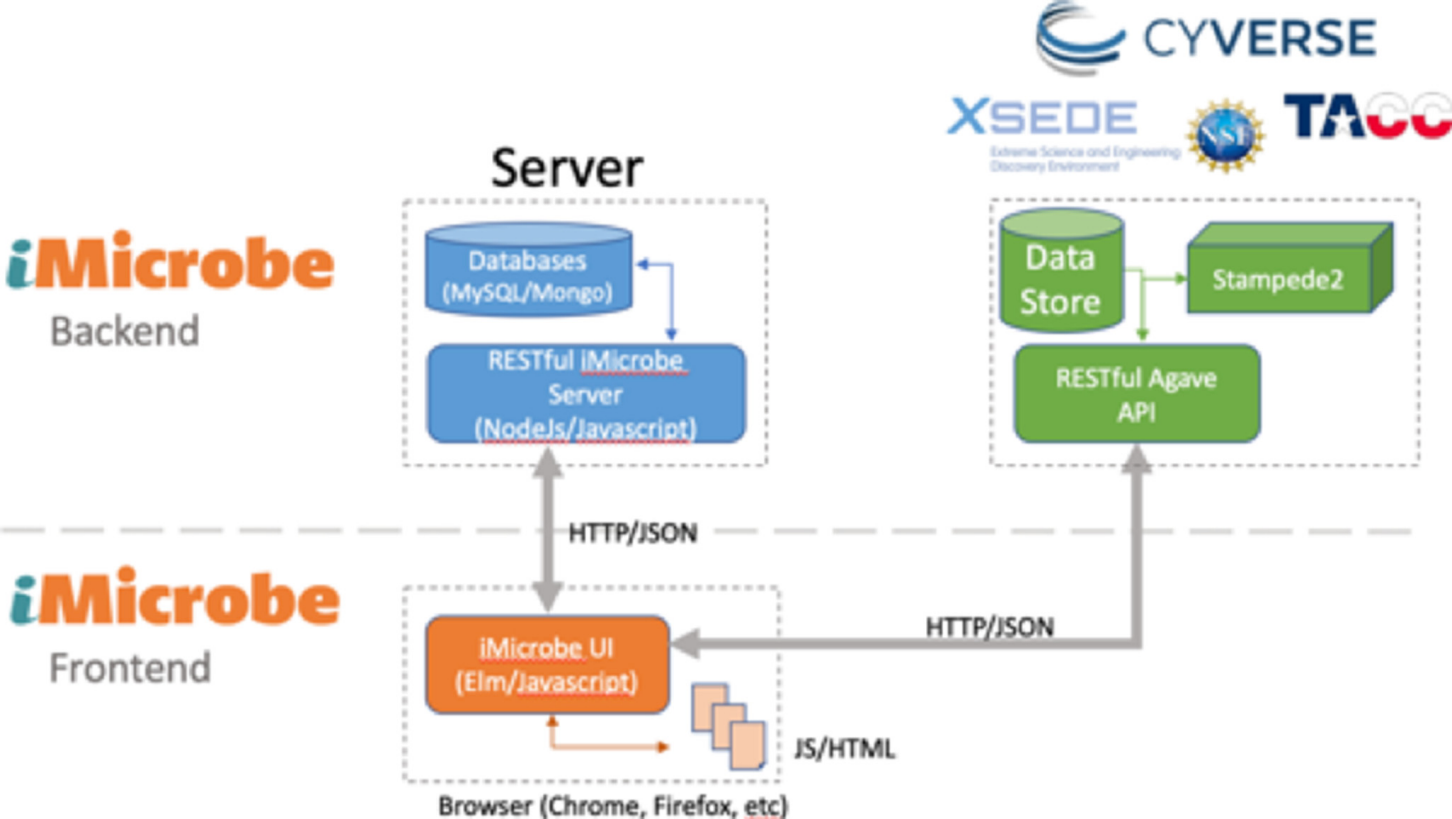
The iMicrobe user interface (UI) comprises a back end written in Node/JS that talks to MySQL and MongoDB databases to deliver JSON to a front end written in Elm, which also communicates with the Agave API for the computing resources of CyVerse Data Store and TACC's Stampede2 HPC cluster.

iMicrobe employs a common web architecture of dividing the “front-end” user interface from a “back-end” API (Fig. 5 ). The front end is written in Elm [61], a purely functional language similar to Haskell [62]. Elm code compiles to the JavaScript that the browser runs to fetch data from the API and format it for the user. The API, written in Node/JS [63], handles requests for data from MySQL [64] and MongoDB [65] and returns data in JSON [66] format. For instance, the projects listing [67] loads in the browser, then makes a request to the API [68] for its data and dynamically creates the table listing. This architecture, while more complicated, leads to better user experience as pages load quickly and then perform longer-running tasks such as requesting and formatting the data. In addition, the data becomes available to third parties who may prefer to use the API to get structured, machine-readable data (JSON) rather than HTML.

**Figure 6:**
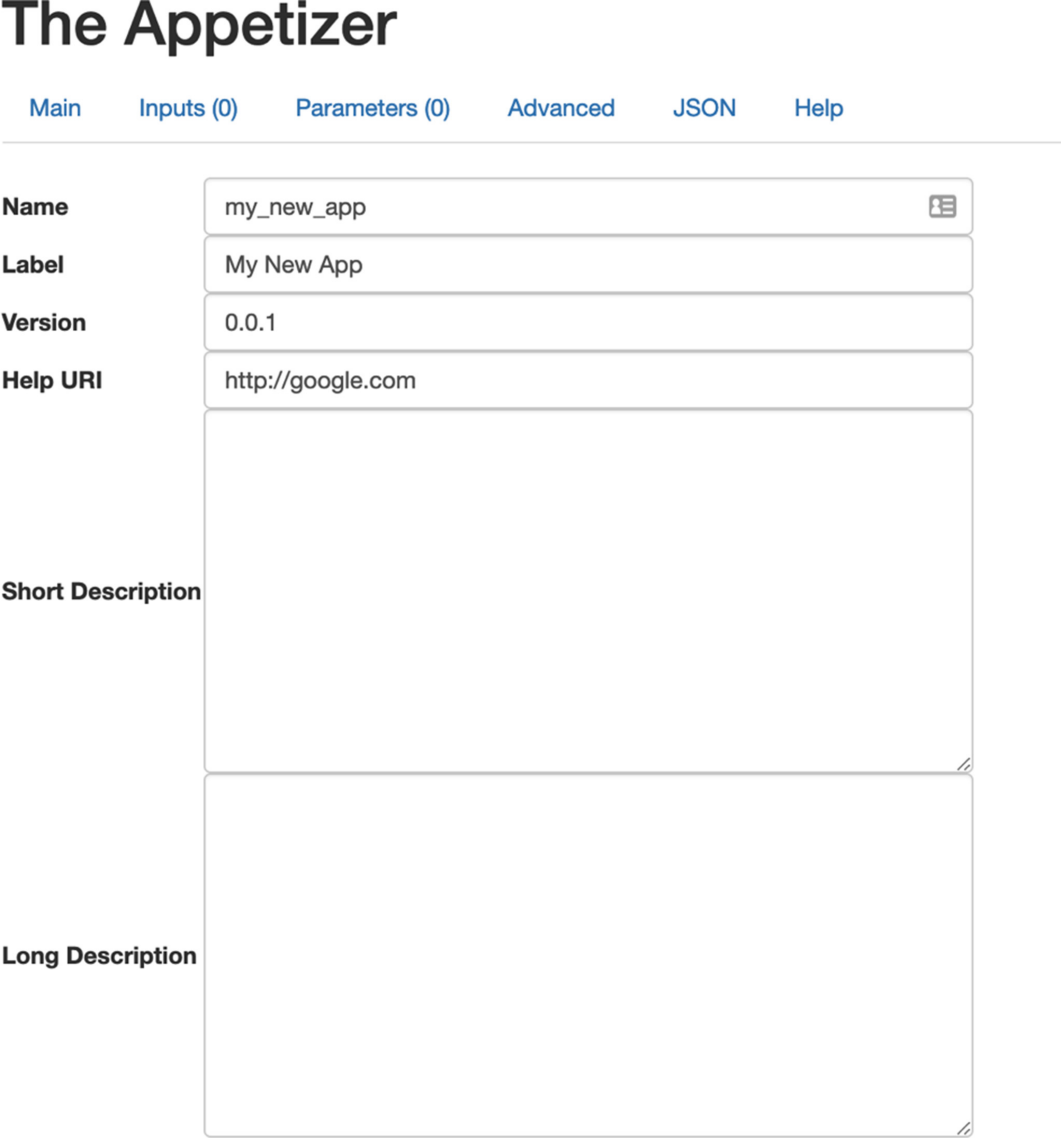
The Appetizer is a web interface to assist developers in creating the JSON file needed to describe an app's inputs and parameters to the Agave API. “Inputs” are data files that need to be copied to the compute node to run. “Parameters” are program settings such as integer or strings values that need to be indicated by the end user. The “Advanced” tab allows the app developer to indicate the requirements of the Stampede2 compute nodes such as RAM, CPU, execution queue, and time. The “JSON” tab allows the user access to the JSON that is generated by the app.

### Databases

All metadata from investigators to projects to samples and sample attributes are stored in a MySQL relational database primarily using the InnoDB table engine to maintain referential integrity of the data. The MyISAM engine is used for the “quick search” table because that employs a “FULLTEXT” index to handle text searching [69]. Sample attributes are a mix of both text and numeric data, e.g., a Longhurst code like “GUIN” or a chlorophyll measurement like “0.82.” One disadvantage of a traditional relational database management system (RDBMS) is that these values must be stored as text. In order to support the mix of numeric and string searching required for the sample metadata search [18], the sample metadata is denormalized and mirrored into a MongoDB in order to take advantage of a much richer search engine. In MongoDB, those values that appear to be numeric are coerced as such enabling range-based queries such as samples with chlorophyll between 5 and 10 in addition to text-based searches. Users may provide lower and/or upper bounds for numeric queries and additionally mix restrictions on textual values until a suitable subset of samples has been found.

### Stampede2

iMicrobe provides access to both large datasets and the tools and computing resources to analyze them through apps that are run on the Stampede2 cluster. iMicrobe uses the Agave API to launch the app and copy input data files from the CyVerse Data Store or any other web-accessible location (e.g., FTP or HTTP) to Stampede2. Users can also request a personal account on Stampede2 in order to directly use the system and all of iMicrobe's databases (e.g., iMicrobe/Ohana BLAST, Centrifuge, UProc) and app containers.

### Docker/Singularity

Almost every app on iMicrobe [49] is deployed in a Singularity container in order to encapsulate a base operating system and all the dependencies of the included tools. Because of security issues when running Docker containers, TACC only allows Singularity containers. If a tool is available as a Docker container, e.g., through Docker Hub [70], it is a simple matter to build a Singularity container. By using containers, users are never required to install any software locally with the exception of Singularity itself. Every Singularity-based app in iMicrobe has a GitHub repository with the definitions and instructions to create the Singularity containers. Users may choose to extend these definitions, as well as modify the code to customize iMicrobe tools.

### Biocontainers

Much work has been done in various scientific communities to package and distribute tools using Docker using Biocontainers [71]. Stampede2 has a directory (/work/projects/singularity/TACC/biocontainers) containing >7,000 such containers that can be leveraged by researchers if they have the resources to run these. For instance, iMicrobe has incorporated the Trim Galore Biocontainer (for quality checking of reads) [72]. A push by other community developers to help create more Docker/Singularity containers and describe them to Agave could greatly increase the number of tools available via Agave and platforms like iMicrobe.

### The Appetizer

If an app is written such that it has a Singularity container and can be run in a batch mode (that is, given arguments and run to completion unattended by a user), it can be made to run on the Stampede2 system via the Agave API by describing the app's required resources, inputs, and parameters in a JSON file [73]. Both Agave and iMicrobe offer web-based interfaces to create this JSON definition. iMicrobe's is called “The Appetizer” [74] (Fig. 6). In this way, the creation of apps is not limited to the developers of iMicrobe. Any developer can package their code, integrate it into Agave, and deploy it to Stampede2. Once an app is made publicly available, it is a simple matter of adding it to the iMicrobe app table to make it available via the app listing. The user interface to launch an app is dynamically generated at runtime from the same JSON that was used to integrate the app and so requires no additional work on the part of iMicrobe developers to make it available to users.

## Conclusions

Understanding complex biological systems requires integration of biological (particularly microbial) processes with characteristics associated with the environment. These complex systems can only be understood in context with other datasets and sampling time points; however, compiling data on microbial diversity and function in a consistent manner where the data can be interlinked, accessible in a single platform, and analyzed using HPC architectures remains challenging despite major innovations in the semantic web and cloud-based computer architectures. The iMicrobe architecture that we describe here moves away from a standard data repository approach to a model where data are housed in diverse data repositories and integrated as needed. We use the CyVerse Agave API to retrieve and compute on diverse microbiome datasets that are potentially massive, requiring more disk space and computing power than the typical microbial ecologist would have. Furthermore, iMicrobe offers developers a framework for deploying tools to compute on these data using XSEDE HPC resources at Stampede2. By crowdsourcing app development, we enable the community to integrate novel tools that are timely and relevant to their research. We also encourage the development of dynamic documentation at protocols.io. The iMicrobe platform allows users to manage their data through the complete data life cycle. Provenance tracking of both primary data and derived files as well as analyses is performed in CyVerse by keeping all files in the analysis directory, along with data products and a log file to maintain information about the job and parameters that were run. By combining all these features, we believe iMicrobe presents a capable platform for large-scale data searching and analysis for the microbiome science community.

## Availability of supporting source code and requirements

Project name: iMicrobe

Project home page: https://imicrobe.us

Documentation: https://hurwitzlab.gitbook.io/imicrobe/

Source code: https://github.com/hurwitzlab/elm-imicrobe-spa, https://github.com/hurwitzlab/node-imicrobe

Operating system(s): Platform independent

Programming language: NA

Other requirements: CyVerse user account (free)

License: MIT

## Availability of supporting data and materials

Data further supporting this work including snapshots of our code are openly available in the GigaScience repositiory, GigaDB [75].

## Abbreviations

API: Application Programming Interface; BAM: Binary Alignment Map; BLAST: Basic Local Alignment Search Tool; CAMERA: Community Cyberinfrastructure for Advanced Microbial Ecology Research and Analysis; CPU: central processing unit; DOE: Department of Energy; EBI: European Bioinformatics Institute; ENA: European Nucleotide Archive; FAIR: Findable Accessible Interoperable Reusable; FTP: File Transfer Protocol; HPC: high-performance computing; HTTP: HyperText Transfer Protocol; IMG: Integrated Microbial Genomes; iRODS: Integrated Rule-Oriented Data System; JGI: Joint Genome Institute; JS: JavaScript; JSON: JavaScript Object Notation; MG-RAST: Metagenomic Rapid Annotations using Subsystems Technology; ORF: open reading frame; QC: quality control; RAM: random access memory; REST: representational state transfer; rRNA: ribosomal RNA; SDK: Software Development Kit; SOAP: Short Oligonucleotide Analysis Package; SRA: Sequence Read Archive; TACC: Texas Advanced Computing Center; WGS: whole-genome shotgun; XSEDE: Extreme Science and Engineering Discovery Environment.

## Competing interests

The authors declare that they have no competing interests.

## Funding

Initial funding for iMicrobe was provided by the Gordon and Betty Moore Foundation's Marine Microbial Initiativegrant 4491. Further development was supported by the National Science Foundation grant 1639588 and the Simons Foundation's SCOPE project (Simons Collaboration on Ocean Processes and Ecology).

## Authors’ contributions

Supervision, Funding acquisition: B.H.; Conceptualization, Writing: B.H., K.Y.C.; Software, Project administration: K.Y.C., M.B., J.L., I.C.; Data curation: K.Y.C., A.P.; Validation: J.H., A.P., E.W.C.

## Supplementary Material

giz083_GIGA-D-19-00107_Original_Submission

giz083_GIGA-D-19-00107_Revision_1

giz083_Response_to_Reviewer_Comments_Original_Submission

giz083_Reviewer_1_Report_Original_Submission Alex Mitchell -- 4/21/2019 Reviewed

giz083_Reviewer_2_Report_Original_Submission Fredrik Boulund -- 5/27/2019 Reviewed

giz083_Reviewer_2_Report_Revision_1 Fredrik Boulund -- 5/31/2019 Reviewed
