# Erratum to: iMicrobe: Tools and data-driven discovery platform for the microbiome sciences

**DOI:** 10.1093/gigascience/giz097

**Published:** 2019-08-01

**Authors:** Ken Youens-Clark, Matt Bomhoff, Alise J Ponsero, Elisha M Wood-Charlson, Joshua Lynch, Illyoung Choi, John H Hartman, Bonnie L Hurwitz

**Affiliations:** 1 Department of Biosystems Engineering, University of Arizona, 1177 E. 4th St, Shantz Building, Room 403, Tucson, AZ, USA 85721-0038; 2 Environmental Genomics and Systems Biology Division, E.O. Lawrence Berkeley National Laboratory, Berkeley, CA, USA; 3 Department of Computer Science, University of Arizona, Tucson, AZ, USA; 4 BIO5 Institute, University of Arizona, Tucson, AZ, USA

## Correction

In the final publication of “iMicrobe: Tools and data-driven discovery platform for the microbiome sciences” by Ken Youens-Clark et al., there was an erroneous typo in the title of the article. The typo has been corrected and the title appears correctly online.

The Publisher regrets this error.

