# Supplementary material for: iMicrobe: Tools and data-driven discovery platform for the microbiome sciences
Source: Gigascience. 2019 Jul 9;8(7):giz083. doi: 10.1093/gigascience/giz083 (PMC6615980; doi:10.1093/gigascience/giz083)

GigaScience

iMicrobe: Tools and data-driven discovery platform for the microbiome sciences

--Manuscript Draft--

**Manuscript Number:**	GIGA-D-19-00107
**Full Title:**	iMicrobe: Tools and data-driven discovery platform for the microbiome sciences
**Article Type:**	Technical Note
**Funding Information:**	Gordon and Betty Moore Foundation (4491) — Dr Bonnie Hurwitz<br>National Science Foundation (1639588) — Mr Ken Youens-Clark<br>Simons Foundation (SCOPE) — Dr Bonnie Hurwitz
**Abstract:**	Background: Scientists have amassed a wealth of microbiome datasets making it possible to study microbes in living systems on a population- or planetary-scale; however, this potential hasn't been fully realized given that the tools, data sets, and computation are available in diverse repositories and locations. To address this challenge, we developed iMicrobe.us, a community-driven microbiome data marketplace and tool exchange for users to integrate their own data and tools with those from the broader community. Findings: The iMicrobe platform brings together analysis tools and microbiome data sets by leveraging National Science Foundation-supported cyberinfrastructure and computing resources from CyVerse, Agave, and XSEDE. The primary purpose of iMicrobe is to provide users with a freely available, web-based platform to (1) maintain and share project data, metadata, and analysis products, (2) search for related public datasets, and (3) use and publish bioinformatics tools that run on highly-scalable computing resources. Analysis tools are implemented in containers that encapsulate complex software dependencies and run on freely available XSEDE resources via the Agave API which can retrieve datasets from the CyVerse Data Store or any web-accessible location (e.g., FTP, HTTP). Conclusions: iMicrobe promotes data integration, sharing, and community-driven tool development by making open source data and tools accessible to the research community in a web-based platform.
**Corresponding Author:**	Bonnie Hurwitz<br><br>UNITED STATES
**Corresponding Author Secondary Information:**	
**Corresponding Author's Institution:**	
**Corresponding Author's Secondary Institution:**	
**First Author:**	Ken Youens-Clark, BA
**First Author Secondary Information:**	
**Order of Authors:**	Ken Youens-Clark, BA<br>Matt Bomhoff, BS<br>Alise Ponsero, PhD<br>Elisha Wood-Charlson, PhD<br>Joshua Lynch, MS<br>Illyoung Choi, PhD<br>John H Hartman, PhD<br>Bonnie Hurwitz, PhD
**Order of Authors Secondary Information:**	

Powered by Editorial Manager® and ProduXion Manager® from Aries Systems Corporation

Additional Information:	
**Question**	**Response**
Are you submitting this manuscript to a special series or article collection?	No
**Experimental design and statistics**<br><br>Full details of the experimental design and statistical methods used should be given in the Methods section, as detailed in our Minimum Standards Reporting Checklist. Information essential to interpreting the data presented should be made available in the figure legends.<br><br>Have you included all the information requested in your manuscript?	Yes
**Resources**<br><br>A description of all resources used, including antibodies, cell lines, animals and software tools, with enough information to allow them to be uniquely identified, should be included in the Methods section. Authors are strongly encouraged to cite Research Resource Identifiers (RRIDs) for antibodies, model organisms and tools, where possible.<br><br>Have you included the information requested as detailed in our Minimum Standards Reporting Checklist?	Yes
**Availability of data and materials**<br><br>All datasets and code on which the conclusions of the paper rely must be either included in your submission or deposited in publicly available repositories (where available and ethically appropriate), referencing such data using a unique identifier in the references and in the "Availability of Data and Materials" section of your manuscript.	Yes

Have you have met the above requirement as detailed in our Minimum Standards Reporting Checklist?	

Powered by Editorial Manager® and ProduXion Manager® from Aries Systems Corporation

Click here to download Manuscript imicrobe_gigascience_technical_note.docx

Title: iMicrobe: Tools and data-driven discovery platform for the microbiome sciences

Authors: Ken Youens-Clark[1], Matt Bomhoff[1], Alise Ponsero[1], Elisha M. Wood-Charlson[2], Joshua Lynch[1], Illyoung Choi[3], John H. Hartman[3], and Bonnie L. Hurwitz[1,4]

[1] Department of Biosystems Engineering, University of Arizona, Tucson, AZ, USA.

[2] Environmental Genomics and Systems Biology Division, E.O. Lawrence Berkeley National Laboratory, Berkeley, California, USA.

[3] Department of Computer Science, University of Arizona, Tucson, Arizona, USA.

[4] BIO5 Institute, University of Arizona, Tucson, Arizona, USA.

Abstract

Background: Scientists have amassed a wealth of microbiome datasets making it possible to study microbes in living systems on a population- or planetary-scale; however, this potential hasn't been fully realized given that the tools, data sets, and computation are available in diverse repositories and locations. To address this challenge, we developed iMicrobe.us, a community-

driven microbiome data marketplace and tool exchange for users to integrate their own data and tools with those from the broader community. **Findings:** The iMicrobe platform brings together analysis tools and microbiome data sets by leveraging National Science Foundation-supported cyberinfrastructure and computing resources from CyVerse, Agave, and XSEDE. The primary purpose of iMicrobe is to provide users with a freely available, web-based platform to (1) maintain and share project data, metadata, and analysis products, (2) search for related public datasets, and (3) use and publish bioinformatics tools that run on highly-scalable computing resources. Analysis tools are implemented in containers that encapsulate complex software dependencies and run on freely available XSEDE resources via the Agave API which can retrieve datasets from the CyVerse Data Store or any web-accessible location (e.g., FTP, HTTP). **Conclusions:** iMicrobe promotes data integration, sharing, and community-driven tool development by making open source data and tools accessible to the research community in a web-based platform.

Keywords

Cyberinfrastructure, cloud computing, bioinformatics, metagenomics.

Introduction

iMicrobe is a platform that connects researchers' own data to published, curated, microbial metagenomic datasets and high-performance computing methods for their analysis [1]. In the last decade, the cost of sequencing has decreased at a rate far outpacing Moore's law, leading to a rapid increase in the number and size biological datasets [2]. Researchers now have access to an

unprecedented scale and variety of data ranging from large-scale ‘omics data to streaming data from sensors. Biologists increasingly need the power and storage of high-performance computing (HPC) clusters to perform analyses; however, most biologists have limited or no access to these resources and often have to run analyses on their own personal computers.

To address the growing need for HPC in computational biology, the National Science Foundation (NSF) has funded shared cyberinfrastructure resources like XSEDE [3] and Stampede2 [4], an 18-petaflop supercomputer at the Texas Advanced Computing Center (TACC) at the University of Texas at Austin. Developers at TACC have created Agave [5], a REST [6] Application Program Interface (API), to interact with Stampede2’s resources including creating and editing apps, scheduling, and monitoring jobs, and viewing and retrieving the results of analysis jobs. The iMicrobe website makes use of the Agave API [7] to create a web-based portal to the CyVerse [8] DataStore [9] and HPC resources such as the Stampede2 HPC via a free CyVerse account.

iMicrobe users can use their web browser to search public metagenomics datasets like CAMERA [10], save data to a cart, upload their own personal data sets, and run over 30 analysis tools on both private and public data sets using free compute on Stampede2 (Fig. 1). All data in iMicrobe is also available via FTP [11] or from the CyVerse Data Store via iRODS [12] (command line) or the CyVerse Data Commons [13] (web browser). The Agave API also allows for direct command-line access to data and pipelines via the CyVerse SDK [14]. Users can login directly to Stampede2 and use iMicrobe’s analysis tools in their Singularity [15] containers or build containers from source to use on their own computing resources. Finally, developers can use the Agave API to create novel tools that can be integrated into iMicrobe.

Figure 1: *iMicrobe's architecture allows for the integration of datasets hosted by iMicrobe which can be placed into the data cart, those private to the user, and others publicly accessible on the Internet. Analyses created by iMicrobe or other developers run on Stampede2, and the results go into the users' home directory in the CyVerse Data Store.*

Findings

iMicrobe leverages CyVerse cyberinfrastructure and XSEDE supercomputing resources

Security & Trustworthiness. iMicrobe leverages the CyVerse cyberinfrastructure (CI) to provide services to users including the OAuth2 authentication system [16] for secure single sign-on between iMicrobe and all CyVerse services as well as the CyVerse Data Store for storing, sharing, and distributing large amounts of data and analyses. Users can also access high-performance computing systems such as XSEDE's Stampede2 cluster to execute analyses that originate from iMicrobe apps.

Usability & Data Storage. iMicrobe uses the CyVerse Data Store for data sets and analysis results. CyVerse Data Store offers solutions to many contemporary data storage needs in the age of large, distributed, digital data. CyVerse's cloud-based data storage is optimized for large data, is free to most scientific researchers, is accessible through multiple interfaces, and leaves access control in the hands of the data owners. The Data Store provides research scientists, research groups, and research organizations with private, shared, or public storage allocations primarily for use within the CyVerse CI. The CyVerse Data Commons houses public data within the Data Store for use by the research community either within or outside CyVerse CI. The Data Store

offers reliable, secure storage for datasets of any size that are actively being used for both research and/or education purposes. Data and metadata in the Data Store are stored in a high-performance storage resource that has built-in redundancy and is continuously monitored for security and failure. The Data Store is synchronously backed up at both the University of Arizona in Tucson, Arizona, and at the Texas Advanced Computing Center in Austin, Texas. All users initially get 100GB of storage but can request additional allocations through CyVerse [17]. Private user data is stored in a user's home folder ("/iplant/home/$user," where $user is a CyVerse username), and will be accessible through a view in iMicrobe where files can be uploaded, deleted, shared, or modified. Users can share data that they own with other registered CyVerse users. Project leaders can request larger allocations for collaborative projects. Project leaders can also request a Community Data Folder that will be made public [18]. Community Data folders are housed in the "iplant/homes/shared" directory and are visible via iMicrobe under "Community Data". Data policies associated with iMicrobe are in sync with CyVerse policies.

Provenance & Reproducibility. iMicrobe data and analysis provenance both primary data, derived files, and analyses are tracked in CyVerse by keeping all files in the analysis directory along with data products and a log file to maintain information about the job and parameters that were run. CyVerse also maintains a job history to allow researchers to track and reproduce experiments. In iMicrobe, data provenance is imperative given that data are derived from diverse data stores that have varied levels of curation and versioning.

General Search

The upper-right corner of every page on iMicrobe has a search box that will perform a simple query over text in our databases related to projects, investigators, samples, taxonomy, and proteins. For instance, a search for “obese” finds 28 hits including a publication, two projects, and the 25 samples from those two projects.

Metadata search

The samples in iMicrobe can be searched by their metadata or “data about the data”. Samples are described in detail by over 200 attributes such as type (artificial metagenome, isolate, metagenome, metatranscriptome, transcriptome), biome (sewage, soil, deep chlorophyll max, acid mine drainage), depth, dissolved oxygen, latitude/longitude, salinity, host organism, chlorophyll, and more. The values for these can be numeric or character values such as a measurement of depth in meters or “Synechococcus” for the host organism. In total, there are over 140K descriptors for our samples, and users can specify an unlimited number of terms using the sample metadata search tool [19].

Figure 2: *The sample metadata search allows users to search over both text and numeric values in any combination. Each additional search attribute updates the discovered samples immediately and restricts the next attributes the user can use to those found in the sample subset. Valid search values are displayed as multi-select boxes for strings and min/max values for numbers.*

Each time a user selects a new attribute such as “phosphate,” the interface determines if the attribute is a character or numeric data. For character data, the user is presented with a multi-

select list if the number of choices is reasonable to aid in selecting the correct strings. For numbers, minimum and maximum fields are shown with placeholders indicating the current min/max values from the database for the current subset of records shown. For example, if “phosphate” is selected as the first attribute, the min/max values are 0.01/3070, respectively, but if the user first selects a “Longhurst Province” of “ARAB” (NW Arabian Upwelling Province) then the “phosphate” values will be only for those samples found in that province which range from 36 to 50. In this way, users may quickly winnow sample searches to those matching their exact criteria and place the results into the cart.

From the sample details page (e.g., https://www.imicrobe.us/#/samples/5189), users can view a sample’s location on Google Maps (if applicable), add the sample to the cart, view and download the associated data products, and examine the sample’s attributes, predicted proteins, and predicted taxonomic classifications.

Data Cart

Throughout the iMicrobe site, users can add an unlimited number of samples of interest to the data cart. Samples can be easily removed, or the cart can be cleared entirely. Using the cart, users can download the associated data products. Users can filter data products (files) by their associated types (e.g., reads, gene calls, predicted proteins, taxonomy classifications). Users can download data either from the CyVerse Data Store or the iMicrobe FTP site [11]. Contents of the cart can also be used as the input to several of iMicrobe’s applications or “apps.” Carts can be saved and shared with other users.

Figure 3: *The data cart holds any number of samples selected by the user. Using the cart view, users can select the associated data files or analysis products to download. Cart contents can also be used as input values for apps.*

Apps

iMicrobe currently hosts more than 30 self-contained applications (“apps”) (Table 1) or analysis pipelines that take some input files and program parameters (Fig. 4), run to completion, and deposit the results into the users CyVerse Data Store home directory. Apps allow users to easily do quality control “QC” (Trim Galore, Trimmomatic), predict proteins (UProc) and genes (Prodigal, MetaGeneAnnotator, FragGeneScan), assemble contigs (Megahit, SOAPdenovo), assign taxonomy (Centrifuge), and cluster genomes (Mash, Libra, Fizkin) (Table 1). In order to view and run apps, the user must first create an account with CyVerse (https://user.cyverse.org). Accounts are free to create and allow iMicrobe to connect in a user’s data in CyVerse including novel datasets they may wish to analyze with an app or the results of running an analysis tool.

Table 1

Apps can allow for new tools to be more quickly disseminated to the community. Often the discovery of a newly published bioinformatics tool leads to the frustration of downloading and compiling source code, resolving dependencies, and fighting with conflicting versions of tools (e.g., gcc or Python/conda). When tools are released as self-contained packages, others can more quickly and easily test the tools on their own datasets. If community developers release tools as containers, they can more easily be made available as apps through Agave and iMicrobe.

Figure 4: *The app launch interface allows users to select input files and set parameters for the app. The input files may come from the users' own Data Store, any publicly available data in the Data Store such as files associated with their data cart or iMicrobe sample files, or any other file available over FTP or HTTP. The Agave API will copy the input files to the compute node when the job is run.*

Making data and tools Findable, Accessible, Interoperable, and Reusable (FAIR)

iMicrobe is pursuing FAIR [20] (Findable, Accessible, Interoperable, and Reusable) principles as it relates to both data and computation. We strive to make data findable via searches; accessible via the CyVerse Data Store, iRODS or FTP; interoperable via common file formats; and reusable via open access. Likewise, we aim to make compute findable via our website, accessible via the Agave API, interoperable via Singularity containers, and reusable via open access. Just as common file formats like FASTA or GFF make data exchange simple, containers like Docker [21] and Singularity allow computational methods to be run on any system and promote reuse.

Reusable

Making data reusable via the dynamic metadata search in iMicrobe. An acute need in the microbiome community is to discover and integrate data in disparate data repositories to facilitate analyses. Specifically, primary and associated contextual metadata, as well as other data products (and their provenance), exist in diverse microbiome data repositories. To make data more discoverable, iMicrobe provides a dynamic search to find data sets based on structured and curated metadata. Data can be added to a shopping cart and analyzed using a variety of tools.

Making tools reusable by creating containers. As for computing resources, users can find iMicrobe apps on the iMicrobe website [22]. Most iMicrobe apps use Singularity containers which can be accessed directly from the Stampede2 file system under a shared iMicrobe directory. The recipes to build iMicrobe's Singularity containers are stored in GitHub repositories (see Table 1), making it possible for users to build and use containers locally on their own datasets.

Accessible

Analyzing remote web-accessible datasets in iMicrobe. Because a much 'omics data lives in diverse repositories globally, moving the data to a central location for processing can be problematic and inefficient. Still, scientists need to bring together diverse data sets to enable population-level or planetary-scale analyses that drive new knowledge and discovery. iMicrobe delivers a virtual framework for connecting web-accessible remote microbiome 'omics data with private user data using the Agave API. Any data sets that are available via a web-link (FTP or HTTP) are accessible and computable in iMicrobe. iMicrobe provides ready access to the CAMERA data collection including reads, peptides, CDS, contigs, assemblies, annotations, as well as related projects derived from the sample and environmental data for 120+ microbiome projects, representing 1 TB of data. These data are hosted in the CyVerse Data Store ("/iplant/shared/imicrobe") and are integrated into iMicrobe under "Community Data". A user can analyze CAMERA data alongside their own personal data sets and also any other data set which the Agave API can retrieve from a public web address.

Interoperable

Containerizing tools and pipelines for making analyses interoperable on diverse compute platforms. Microbiome science methods and bioinformatics code are constantly evolving and can be cumbersome to install [23]. Moreover, users may not have access to the computing resources required to run the tool on their data. To this end, iMicrobe converts tools into Singularity containers, packaged virtual machines that encapsulate the operating system, dependencies, and tool's code to ensure reproducibility and allow the code to run on any computational architecture including the Stampede2 HPC. Community developers can contribute tools by releasing containers to Biocontainers or Docker Hub. Currently, most iMicrobe apps are deployed at TACC Stampede2 using the Agave API; however, the containerized tools can theoretically be run on any computer resource including cloud resources such as Amazon Web Services (AWS) or Google Cloud Platform. The iMicrobe platform automatically creates a user interface to launch a container by using a JSON (JavaScript Object Notation) description of the app that encodes its inputs and parameters. The JSON also specifies hardware requirements (CPU and memory) to run the tool, e.g., on the default queue or a high-memory node at Stampede2. iMicrobe streamlines community-driven tool development and accessibility to a variety of tools in a simple web-based platform.

Running Analyses through the iMicrobe web-based platform. To run an analysis, users select an app from the "apps" listing; select data "inputs" from their own CyVerse Data Store, publicly available data in their shopping cart, or some publicly-accessible URL; select parameters; and launch the tool with the click of the "Run" button. Users can track the status of their jobs directly on the site and view results and interactive data visualizations. As with all files, users can share analysis results with collaborators. Provenance of primary data derived files, and analyses are

tracked in CyVerse by keeping all files in the analysis directory, along with data products and a log file record about the job, including data sources, app versioning, and the parameters selected for that run. CyVerse also maintains the job history to allow researchers to track and reproduce other researcher's experiments.

Reusable

Virtual communities, protocols, and documentation for iMicrobe. Given the experimental nature of methods in microbiome research, iMicrobe fosters discussions about both molecular and computational protocols with an eye towards improving methods. iMicrobe partners with protocols.io [24], a method-centered collaborative platform, to provide guides and sample data sets for popular use cases in microbiome research. These methods are available through the iMicrobe virtual community [25]. Through protocols.io, scientists can also create and share their own protocols and/or groups in the microbiome sciences. Users can also access documentation on using the iMicrobe website and protocols through the iMicrobe Gitbook [26].

Comparison to Web-based Metagenomics Platforms

There are many tools for metagenomic analysis many of which are native desktop applications for Windows, Apple, or Linux operating systems. As iMicrobe is a web application focused on connecting remotely hosted data sets to large compute capacity, we have chosen several similar systems to which we compare.

KBase

The Department of Energy's (DOE) Systems Biology Knowledgebase [27] is "an open-access bioinformatics software and data platform for analyzing plants, microbes, and their communities." KBase offers several dozen "apps" which can be organized into workflows called "narratives." To create a novel app, users must apply to for a KBase developer account and install the KBase Software Development Kit (SDK) and dependencies (Java 1.7, Python 2.7, NodeJS, Bower, Docker), and work with the KBase staff to integrate the app. Apps run on computer resources at Lawrence Berkeley National Lab (LBNL) or Argonne National Lab (ANL) with plans to expand to other DOE HPC or cloud resources. In contrast, developers who wish to create a CyVerse or iMicrobe app are simply required to put that app into a Singularity container and describe the inputs and parameters using a JSON document. As described below, web-based interfaces can be used to create the necessary JSON app definitions.

MGnify

MGnify [28] is metagenomics platform from the European Bioinformatics Institute (EBI). Users can publicly archive their data and receive a permanent accession which can be used to retrieve sequences and metadata via EBI's European Nucleotide Archive (ENA). After releasing data to ENA, MGnify integrates metagenomic datasets and may run one or more versions of their standard pipeline on datasets [29]. Metadata searches in MGnify include temperature, depth, biome, sequencing method, and a few other fields. In contrast, all metadata fields are available in iMicrobe via the sample/metadata search. Users are not able to run MGnify analysis pipelines directly but may submit a request to analyze private or public datasets. In contrast, iMicrobe

users can run apps (which include analysis pipelines) directly from the integrated app and job submission interface as described above.

MG-RAST

MG-RAST [30], or the metagenomics rapid annotation using subsystems technology server, makes it possible for users to upload raw metagenomic sequence data in FASTQ or FASTA format. Assessments of sequence quality and annotation with respect to multiple reference databases are performed automatically with minimal input from the user. Post-annotation analysis and visualization are also possible, directly through the web interface, or with tools like matR (metagenomic analysis tools for R) that use the MG-RAST API [31] to download. Similar to MGnify, a single comprehensive analysis pipeline is applied to all user datasets. By contrast, iMicrobe users can upload any data type relevant to any app and run them in a modular fashion and adjust the parameters to be more specific to their data and research question.

IMG/M

The Integrated Microbial Genomes & Microbiomes “IMG/M” [32] is a service that supports “the annotation, analysis, and distribution of microbial genome and microbiome datasets sequenced at DOE's Joint Genome Institute (JGI).” Users can use IMG/M for “annotation, analysis, and distribution of their own genome and microbiome datasets” but cannot create and distribute novel tools for community access. Like MGnify and MG-RAST, IMG/M provides uses with a single comprehensive pipeline for analysis. As described previously, iMicrobe provides users with apps that are self-contained and can be run in any order, with user-defined parameters.

QIITA

QIITA [33] allows users to upload and analyze data sets using QIIME2 and GNPS, tools necessarily focused on bacterial species identification via the 16S rRNA gene. While iMicrobe hosts a 16S clustering tool [34], many other general purpose tools exist for QC, trimming, read assembly, pairwise sequence alignment, and gene functional annotation, among others, and users are not limited to analysis tools which use this bacterial marker gene.

Methods

Architecture

iMicrobe employs a common web architecture of dividing the “front-end” user interface from a “back-end” API (application programming interface; Fig. 5). The front-end is written in Elm [35], a purely functional language similar to Haskell [36]. Elm code compiles to the JavaScript that the browser runs to fetch data from the API and format it for the user. The API, written in Node/JS [37], handles requests for data from MySQL [38] and MongoDB [39] and returns data in JSON [40] format. For instance, the projects listing [41] loads in the browser then makes a request to the API [42] for its data and dynamically creates the table listing. This architecture, while more complicated, leads to better user experience as pages load quickly and then perform longer-running tasks such as requesting and formatting the data. In addition, the data becomes available to third parties who may prefer to use the API to get structured, machine-readable data (JSON) rather than HTML.

Figure 5: *The iMicrobe user interface is comprised of a back-end written in Node/JS that talks to MySQL and MongoDB databases to deliver JSON to a front-end written in Elm which also communicates with the Agave API for the computing resources of CyVerse Data Store and TACC's Stampede2 HPC cluster.*

Databases

All metadata from investigators to projects to samples and sample attributes are stored in a MySQL relational database primarily using the InnoDB table engine to maintain referential integrity of the data. The MyISAM engine is used for the "quick search" table as that employs a "FULLTEXT" index to handle text searching [43]. Sample attributes are a mix of both text and numeric data, e.g., a Longhurst code like "GUIN" or a chlorophyll measurement like "0.82." One disadvantage of a traditional relational database management systems (RDBMS) is that these values must be stored as text. In order to support the mix of numeric and string searching required for the sample metadata search [19], the sample metadata is denormalized and mirrored into a MongoDB in order to take advantage of a much richer search engine. In MongoDB, those values that appear to be numeric are coerced as such enabling range-based queries such as samples with chlorophyll between 5 and 10 in addition to text-based searches. Users may provide lower and/or upper bounds for numeric queries and additionally mix restrictions on textual values until a suitable subset of samples has been found.

Stampede2

iMicrobe provides access to both large datasets and the tools and computing resources to analyze them through apps which are run on the Stampede2 cluster. iMicrobe uses the Agave API to

launch the app and copy input data files from the CyVerse Data Store or any other web-accessible location (e.g., FTP or HTTP) to Stampede2. Users can also request a personal account on Stampede2 in order to directly use the system and all iMicrobe's databases (e.g., iMicrobe/Ohana BLAST, Centrifuge, UProc) and app containers.

Docker/Singularity

Almost every app on iMicrobe [22] is deployed in a Singularity container in order to encapsulate a base operating system and all the dependencies of the included tools. Because of security issues when running Docker containers, TACC only allows Singularity containers. If a tool is available as a Docker container, e.g., through Docker Hub [44], it is a simple matter to build a Singularity container. By using containers, users are never required to install any software locally with the exception of Singularity itself. Every Singularity-based app in iMicrobe has a GitHub repository with the definitions and instructions to create the Singularity containers. Users may choose to extend these definitions as well as modify the code to customize iMicrobe tools.

Biocontainers

Much work has been done in various scientific communities to package and distribute tools using Docker using Biocontainers [45]. Stampede2 has a directory (/work/projects/singularity/TACC/biocontainers) containing over 7,000 such containers which can be leveraged by researchers if they have the resources to run these. For instance, iMicrobe has incorporated the Trim Galore Biocontainer (for quality checking of reads) [46]. A push by other community developers to help create more Docker/Singularity containers and describe

them to Agave could greatly increase the number of tools available via Agave and platforms like iMicrobe.

The Appetizer

If an app is written such that it has a Singularity container and can be run in a batch mode (that is, given arguments and run to completion unattended by a user), it can be made to run on the Stampede2 system via the Agave API by describing the app's required resources, inputs, and parameters in a JSON file [47]. Both Agave and iMicrobe offer web-based interfaces to create this JSON definition. iMicrobe's is called "The Appetizer" [48]. In this way, the creation of apps is not limited to the developers of iMicrobe. Any developer can package their code, integrate it into Agave, and deploy it to Stampede2. Once an app is made publicly available, it is a simple matter of adding it to the iMicrobe "app" table to make it available via the app listing. The user interface to launch an app is dynamically generated at runtime from the same JSON that was used to integrate the app and so requires no additional work on the part of iMicrobe developers to make it available to users.

Figure 6: *The Appetizer is a web interface to assist developers in creating the JSON file needed to describe an app's inputs and parameters to the Agave API. "Inputs" are data files that need to be copied to the compute node to run. "Parameters" are program settings such as integer or strings values that need to be indicated by the end user. The "Advanced" tab allows the app developer to indicate the requirements of the Stampede2 compute nodes such as RAM, CPU, execution queue and time. The "JSON" tab allows the user access to the JSON that is generated by the app.*

Conclusions

Understanding complex biological systems require integration of biological (particularly microbial) processes with characteristics associated with the environment. These complex systems can only be understood in context with other datasets and sampling time points; however, compiling data on microbial diversity and function in a consistent manner where the data can be interlinked, accessible in a single platform, and analyzed using high-performance computer architectures remains challenging despite major innovations in the semantic web and cloud-based computer architectures. The iMicrobe architecture we describe here moves away from a standard data repository approach to a model where data are housed in diverse data repositories and integrated as needed. We use the CyVerse Agave API to retrieve and compute on diverse microbiome datasets that are potentially massive, requiring more disk space and computing power than the average microbial ecologist would have. Further, iMicrobe offers developers a framework for deploying tools to compute on these data using XSEDE HPC resources at Stampede2. By crowdsourcing app development, we enable the community to integrate novel tools that are timely and relevant to their research. We also encourage the development of dynamic documentation at protocols.io. The iMicrobe platform allows users to manage their data through the complete data lifecycle. Provenance tracking of both primary data, derived files, and analyses are tracked in CyVerse by keeping all files in the analysis directory, along with data products and a log file to maintain information about the job and parameters that were run. By combining all these features, we believe iMicrobe presents a capable platform for large-scale data searching and analysis for the microbiome science community.

Availability of supporting source code and requirements

- Project name: iMicrobe
- Project home page: https://imicrobe.us
- Documentation: https://hurwitzlab.gitbook.io/imicrobe/
- Source code: https://github.com/hurwitzlab/elm-imicrobe-spa, https://github.com/hurwitzlab/node-imicrobe
- Operating system(s): e.g. Platform independent
- Programming language: NA
- Other requirements: CyVerse user account (free)
- License: e.g., MIT
- RRID: NA

Funding

Initial funding for iMicrobe was provided by the Gordon and Betty Moore Foundation's Marine Microbial Initiative grant #4491. Further development was supported by the National Science Foundation grant #1639588 and the Simons Foundation's SCOPE project (Simons Collaboration on Ocean Processes and Ecology).

Acknowledgements

We would like to thank Jon Kaye at the Gordon Betty Moore Foundation; our sysadmin, Adam Michel, at UA; Lenny Teytelman and Alexie Stoliartchouk at protocols.io; Nirav Merchant and Ramona Walls at CyVerse; and Matt Vaughn and John Fonner at TACC. This work used the Extreme Science and Engineering Discovery Environment (XSEDE), which is supported by National Science Foundation grant number ACI-1548562.

Table 1. *Current list of available apps in iMicrobe.*

**App Name**	**Purpose**
16s_cluster-0.0.1u2	Cluster 16S sequences [34]
c-microbial-map-0.0.1u1	Visualize geographic distribution of 16S sequences in the ocean [49]
centrifuge-1.0.4u1	Short-read taxonomic classification [50]
centrifuge-bubble-0.0.5u1	Visualization of Centrifuge analysis [51]
ClusterGenomes-1.1.3u2	Clusters genomes based on all-verses-all alignments
DIAMOND-0.9.10u1	Fast read alignment of DNA or proteins [52]
fizkin-0.0.3u1	Pairwise sample comparison via kmers [53]
FragGeneScan-1.30.0u1	Short-read ORF prediction [54]
graftm-0.11.1u3	Rapid community profiles from metagenomes [55]
imicrobe-demultiplexer-0.0.1u1	Demultiplexing pipeline for single and paired-end data [56]
imicrobe-megahit-0.0.2u1	Metagenomics read assembler [57]
imicrobe-prokka-0.0.2u1	Prokaryotic genome annotation [58]
imicrobe-soapdenovo2-0.0.3u1	Short-read assembler [59]
libra-1.0	Pairwise sample comparison via kmers [60]

MArVD-1.0.0u1	Metagenomic Archaeal Virus Detector [61]
mash-all-vs-all-0.0.5u1	Pairwise sample comparison via Mash [62]
MetaGeneAnnotator-1.1.0u1	Prokaryotic and phage gene prediction [63]
ohana-blast-0.0.9u2	BLAST search to Ohana gene catalog [64]
prodigal-2.6.3u3	Gene prediction [65]
Prokka-1.12.0u2	Prokaryotic genome annotation [58]
puma-0.3.0u1	Annotation of HPV genomes [66]
Read2RefMapper-1.1.0u2	Filtering coverage of BAM files to a reference dataset [67]
sra-fastq-dump-0.0.1u1	Save sequences from SRA in CyVerse DataStore [68]
trim-galore-0.4.5u1	Quality control tool for trimming reads [69]
Trimmomatic-0.36.0u2	Quality control tool for trimming reads [70]
uproc_dna-1.2.0u3	Protein sequence classification [71]
vContact-0.1.60u2	Viral Contig Automatic Cluster Taxonomy [72]
vContact_PCs-0.1.60u2	Viral Contig Automatic Cluster Taxonomy [72]
WIsH-Build-1.0.0u2	Identify bacterial hosts from metagenomic data [73]
WIsH-Predict-1.0.0u2	Identify bacterial hosts from metagenomic data [73]

References

1. Youens-Clark K, Bomhoff M, Hurwitz BL. iMicrobe [Internet]. Available from: https://imicrobe.us

2. Wetterstrand KA. DNA Sequencing Costs: Data from the NHGRI Genome Sequencing Program (GSP) [Internet]. Available from: https://www.genome.gov/27541954/dna-sequencing-costs-data/

3. Towns J, Cockerill T, Dahan M, Foster I, Gaither K, Grimshaw A, et al. XSEDE: Accelerating Scientific Discovery. Computing in Science Engineering. 2014;16:62–74.

4. Texas Advanced Computing Center (TACC), The University of Texas at Austin.

5. Dooley R, Brandt SR, Fonner J. The Agave Platform: An Open, Science-as-a-Service Platform for Digital Science. Proceedings of the Practice and Experience on Advanced Research Computing. ACM; 2018. p. 28.

6. Fielding R. Representational state transfer. Architectural Styles and the Design of Netowork-based Software Architecture. 2000;76–85.

7. Dooley R. Agave API [Internet]. Available from: http://agaveapi.co/

8. Merchant N, Lyons E, Goff S, Vaughn M, Ware D, Micklos D, et al. The iPlant Collaborative: Cyberinfrastructure for Enabling Data to Discovery for the Life Sciences. PLoS Biol. 2016;14:e1002342.

9. CyVerse Data Store [Internet]. Available from: https://www.cyverse.org/data-store

10. Seshadri R, Kravitz SA, Smarr L, Gilna P, Frazier M. CAMERA: a community resource for metagenomics. PLoS Biol. 2007;5:e75.

11. Youens-Clark K, Bomhoff M, Hurwitz BL. iMicrobe FTP [Internet]. Available from: ftp.imicrobe.us

12. Rajasekar A, Moore R, Hou C-Y, Lee CA, Marciano R, de Torcy A, et al. iRODS Primer: Integrated Rule-Oriented Data System. Synthesis Lectures on Information Concepts, Retrieval, and Services. Morgan & Claypool Publishers; 2010;2:1–143.

13. CyVerse Data Commons [Internet]. Available from: http://datacommons.cyverse.org/

14. Vaughn M, Carson J. CyVerse Software Development Kit [Internet]. Available from: https://github.com/cyverse/cyverse-sdk

15. Kurtzer GM, Sochat V, Bauer MW. Singularity: Scientific containers for mobility of compute. PLoS One. 2017;12:e0177459.

16. Hammer-Lahav DE, Hardt D. The oauth2. 0 authorization protocol. 2011. Technical report, IETF Internet Draft. 2011;

17. CyVerse Data Policy [Internet]. Available from: https://www.cyverse.org/data-policy

18. Publishing Data Through The Data Commons [Internet]. Available from: https://wiki.cyverse.org/wiki/display/DC/Publishing+Data+through+the+Data+Commons

19. Youens-Clark K, Bomhoff M. iMicrobe Samples Metadata Search [Internet]. Available from: https://www.imicrobe.us/#/samples

20. Wilkinson MD, Dumontier M, Aalbersberg IJJ, Appleton G, Axton M, Baak A, et al. The FAIR Guiding Principles for scientific data management and stewardship. Sci Data. 2016;3:160018.

21. Merkel D. Docker: lightweight Linux containers for consistent development and deployment. Linux J. Belltown Media; 2014;2014:2.

22. Youens-Clark K, Bomhoff M. iMicrobe Apps [Internet]. Available from: https://www.imicrobe.us/#/apps

23. Mangul S, Martin LS, Eskin E, Blekhman R. Improving the usability and archival stability of bioinformatics software. Genome Biol. 2019;20:47.

24. Teytelman L, Stoliartchouk A, Kindler L, Hurwitz BL. Protocols.io: Virtual Communities for Protocol Development and Discussion. PLoS Biol. 2016;14:e1002538.

25. Hurwitz BL, Ponsero AJ, Youens-Clark K, Bomhoff M. Protocols.io iMicrobe Group [Internet]. Available from: http://www.protocols.io/groups/imicrobe

26. Hurwitz BL, Ponsero A, Youens-Clark K, Bomhoff M. iMicrobe Documentation [Internet]. Available from: https://hurwitzlab.gitbook.io/imicrobe/

27. Arkin AP, Stevens RL, Cottingham RW, Maslov S, Henry CS, Dehal P, et al. The DOE Systems Biology Knowledgebase (KBase) [Internet]. bioRxiv. 2016 [cited 2019 Apr 1]. p. 096354. Available from: https://www.biorxiv.org/content/10.1101/096354v1.abstract

28. MGnify [Internet]. Available from: https://www.ebi.ac.uk/metagenomics

29. MGnify Pipelines [Internet]. Available from: https://www.ebi.ac.uk/metagenomics/pipelines/

30. Wilke A, Bischof J, Gerlach W, Glass E, Harrison T, Keegan KP, et al. The MG-RAST metagenomics database and portal in 2015. Nucleic Acids Res. 2016;44:D590–4.

31. Meyer F. MG-RAST API [Internet]. Available from: http://api.metagenomics.anl.gov/api.html

32. Markowitz VM, Chen I-MA, Palaniappan K, Chu K, Szeto E, Pillay M, et al. IMG 4 version of the integrated microbial genomes comparative analysis system. Nucleic Acids Res. 2014;42:D560–7.

33. Gonzalez A, Navas-Molina JA, Kosciolek T, McDonald D, Vázquez-Baeza Y, Ackermann G, et al. Qiita: rapid, web-enabled microbiome meta-analysis. Nat Methods. 2018;15:796–8.

34. Miller M. 16s_cluster-0.0.1u2 [Internet]. Available from: https://github.com/hurwitzlab/imicrobe-16SrDNA-OTU-Clustering

35. Czaplicki E. Elm: Concurrent frp for functional guis. Senior thesis, Harvard University. 2012;

36. Jones SP. Haskell 98 Language and Libraries: The Revised Report. Cambridge University Press; 2003.

37. Surhone LM, Tennoe MT, Henssonow SF. Node.Js. Mauritius: Betascript Publishing; 2010.

38. MySQL Relational Database Management System [Internet]. Available from: https://dev.mysql.com/

39. Chodorow K, Dirolf M. MongoDB: The Definitive Guide. O’Reilly Media; 2010.

40. Crockford D. JSON RFC [Internet]. Available from: https://www.ietf.org/rfc/rfc4627.txt

41. Youens-Clark K, Bomhoff M. iMicrobe Projects [Internet]. Available from: https://www.imicrobe.us/#/projects

42. Youens-Clark K, Bomhoff M. iMicrobe Projects API [Internet]. Available from: https://www.imicrobe.us/api/v1/projects

43. MySQL FULLTEXT Indexes [Internet]. Available from: https://dev.mysql.com/doc/refman/5.5/en/fulltext-search.html

44. Docker Hub [Internet]. Available from: https://hub.docker.com/

45. da Veiga Leprevost F, Grüning BA, Alves Aflitos S, Röst HL, Uszkoreit J, Barsnes H, et al. BioContainers: an open-source and community-driven framework for software standardization. Bioinformatics. academic.oup.com; 2017;33:2580–2.

46. Youens-Clark K. Trim Galore Biocontainer/Singularity [Internet]. Available from: https://github.com/hurwitzlab/trim-galore

47. Dooley R. Agave App Inputs and Parameters [Internet]. Available from: http://developer.agaveapi.co/#inputs-and-parameters

48. Youens-Clark K. The Appetizer [Internet]. Available from: http://appetizer.hurwitzlab.org/

49. Youens-Clark K, McNichol J. C-Microbial-Map [Internet]. Available from: https://github.com/simonscmap/c-microbial-map

50. Kim D, Song L, Breitwieser FP, Salzberg SL. Centrifuge: rapid and sensitive classification of metagenomic sequences. Genome Res. 2016;26:1721–9.

51. Thornton J, Youens-Clark K. Centrifuge Bubble Plot [Internet]. Available from:

https://github.com/hurwitzlab/centrifuge-bubble

52. Buchfink B, Xie C, Huson DH. Fast and sensitive protein alignment using DIAMOND. Nat Methods. 2015;12:59–60.

53. Youens-Clark K, Hurwitz B. Fizkin [Internet]. Available from: https://github.com/hurwitzlab/fizkin

54. Rho M, Tang H, Ye Y. FragGeneScan: predicting genes in short and error-prone reads. Nucleic Acids Res. 2010;38:e191.

55. Boyd JA, Woodcroft BJ, Tyson GW. GraftM: a tool for scalable, phylogenetically informed classification of genes within metagenomes. Nucleic Acids Res. 2018;46:e59.

56. Miller M. iMicrobe Demultiplexer [Internet]. Available from: https://github.com/mattmiller899/demultiplex_app

57. Li D, Liu C-M, Luo R, Sadakane K, Lam T-W. MEGAHIT: an ultra-fast single-node solution for large and complex metagenomics assembly via succinct de Bruijn graph. Bioinformatics. 2015;31:1674–6.

58. Seemann T. Prokka: rapid prokaryotic genome annotation. Bioinformatics. 2014;30:2068–9.

59. Xie Y, Wu G, Tang J, Luo R, Patterson J, Liu S, et al. SOAPdenovo-Trans: de novo transcriptome assembly with short RNA-Seq reads. Bioinformatics. 2014;30:1660–6.

60. Choi I, Ponsero AJ, Bomhoff M, Youens-Clark K, Hartman JH, Hurwitz BL. Libra: scalable k-mer-based tool for massive all-vs-all metagenome comparisons. Gigascience [Internet]. 2019;8. Available from: http://dx.doi.org/10.1093/gigascience/giy165

61. Vik DR, Roux S, Brum JR, Bolduc B, Emerson JB, Padilla CC, et al. Putative archaeal viruses from the mesopelagic ocean. PeerJ. 2017;5:e3428.

62. Ondov BD, Treangen TJ, Melsted P, Mallonee AB, Bergman NH, Koren S, et al. Mash: fast genome and metagenome distance estimation using MinHash. Genome Biol. 2016;17:132.

63. Noguchi H, Taniguchi T, Itoh T. MetaGeneAnnotator: detecting species-specific patterns of ribosomal binding site for precise gene prediction in anonymous prokaryotic and phage genomes. DNA Res. 2008;15:387–96.

64. Mende DR, Bryant JA, Aylward FO, Eppley JM. Environmental drivers of a microbial genomic transition zone in the ocean's interior. Nature [Internet]. nature.com; 2017; Available from: https://www.nature.com/articles/s41564-017-0008-3

65. Hyatt D, Chen G-L, Locascio PF, Land ML, Larimer FW, Hauser LJ. Prodigal: prokaryotic gene recognition and translation initiation site identification. BMC Bioinformatics. 2010;11:119.

66. Van Doorslaer K, Pace J, Youens-Clark K, Freeman C. Puma [Internet]. Available from: https://github.com/KVD-lab/puma

67. Bolduc B, Roux S. Ref2ReadMapper [Internet]. Available from: https://bitbucket.org/bolduc/docker-read2refmapper/

68. Bomhoff M. SRA FASTQ Dump [Internet]. Available from: https://github.com/hurwitzlab/fastq-dump

69. Krueger F. Trim galore. A wrapper tool around Cutadapt and FastQC to consistently apply quality and adapter trimming to FastQ files. 2015;

70. Bolger AM, Lohse M, Usadel B. Trimmomatic: a flexible trimmer for Illumina sequence data. Bioinformatics. 2014;30:2114–20.

71. Meinicke P. UProC: tools for ultra-fast protein domain classification. Bioinformatics. 2015;31:1382–8.

72. Bolduc B, Jang HB, Doulcier G, You Z-Q, Roux S, Sullivan MB. vConTACT: an iVirus tool to classify double-stranded DNA viruses that infect Archaea and Bacteria. PeerJ. 2017;5:e3243.

73. Galiez C, Siebert M, Enault F, Vincent J, Söding J. WIsH: who is the host? Predicting prokaryotic hosts from metagenomic phage contigs. Bioinformatics. 2017;33:3113–4.

Click here to download Figure figure_1.png

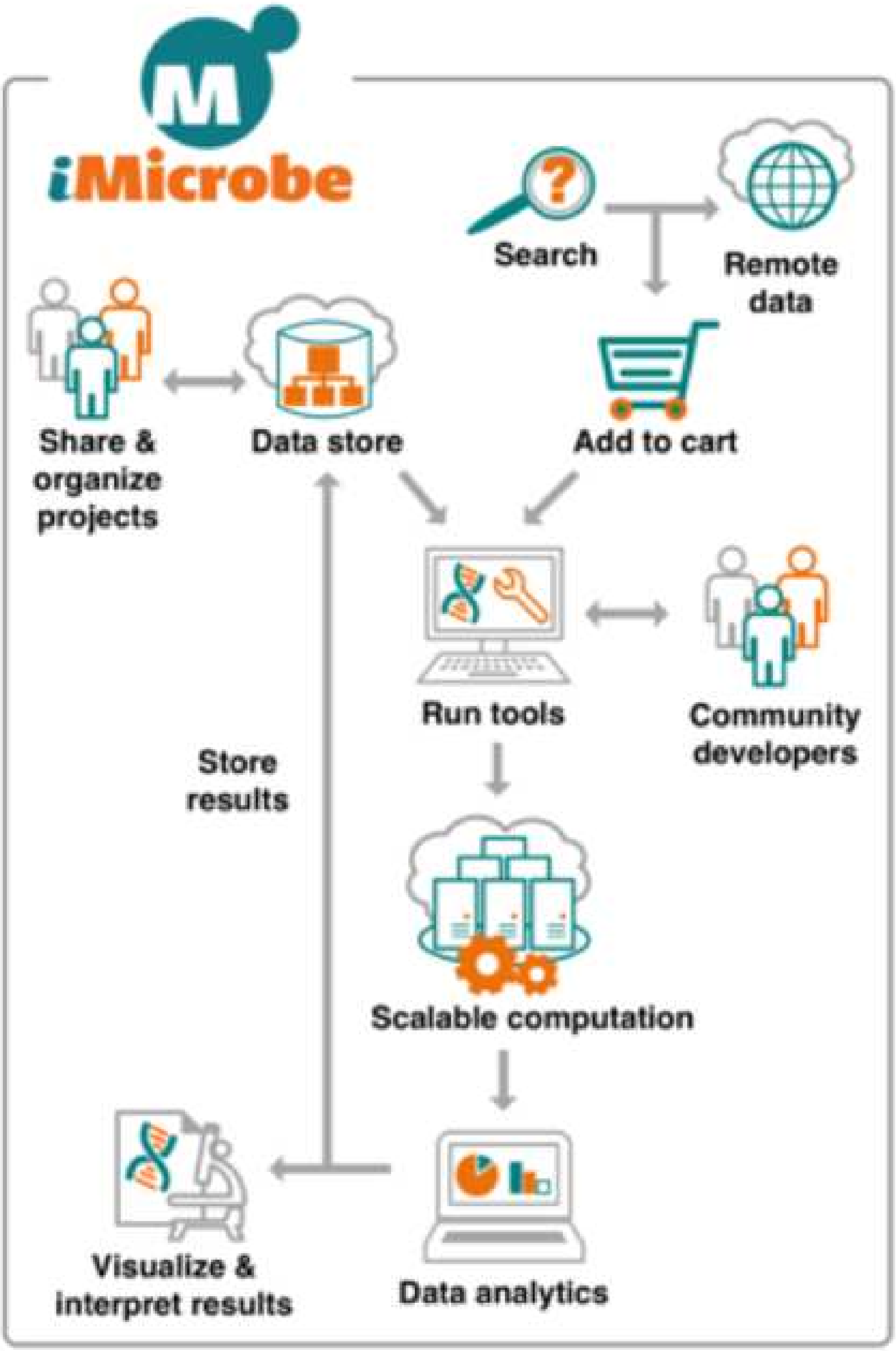

Click here to download Figure figure_2.png

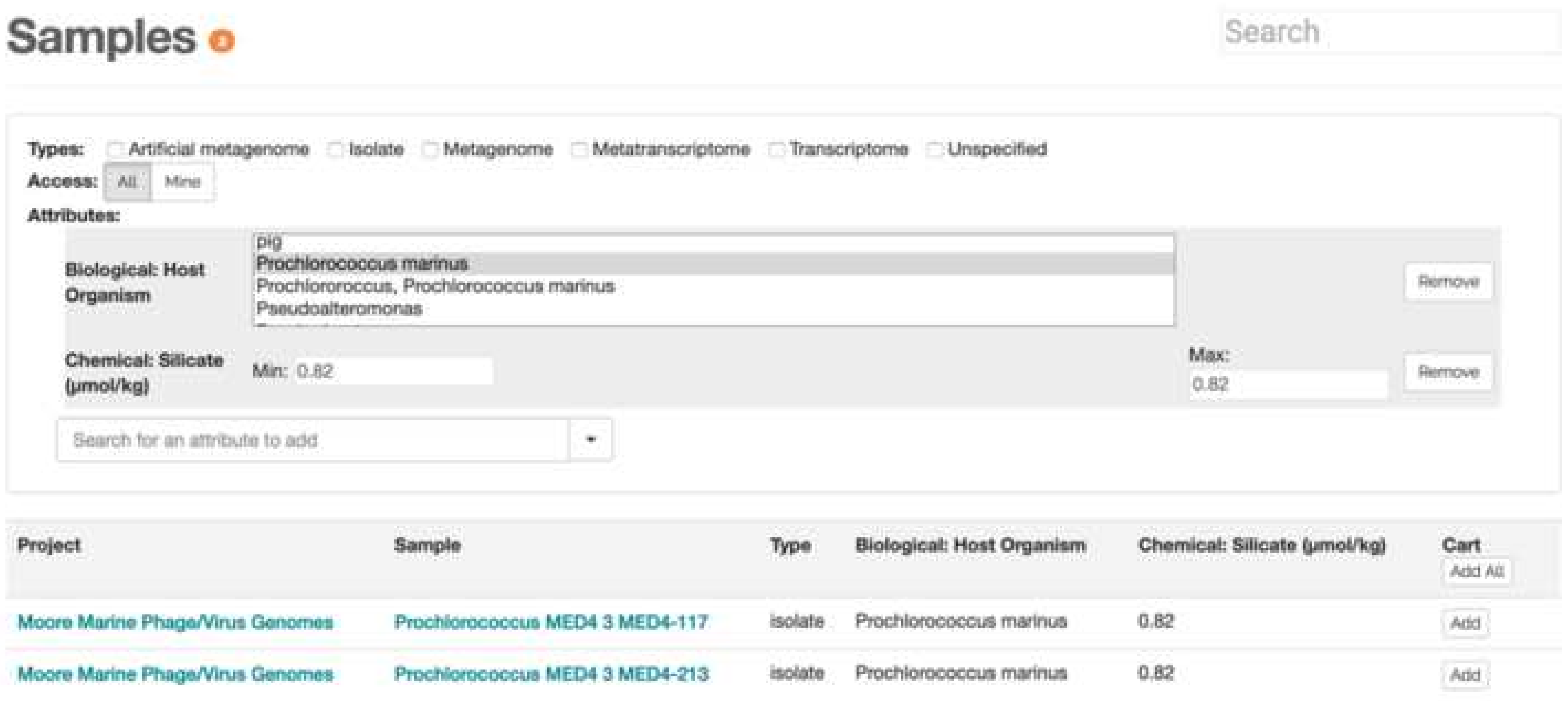

Click here to download Figure figure_3.png

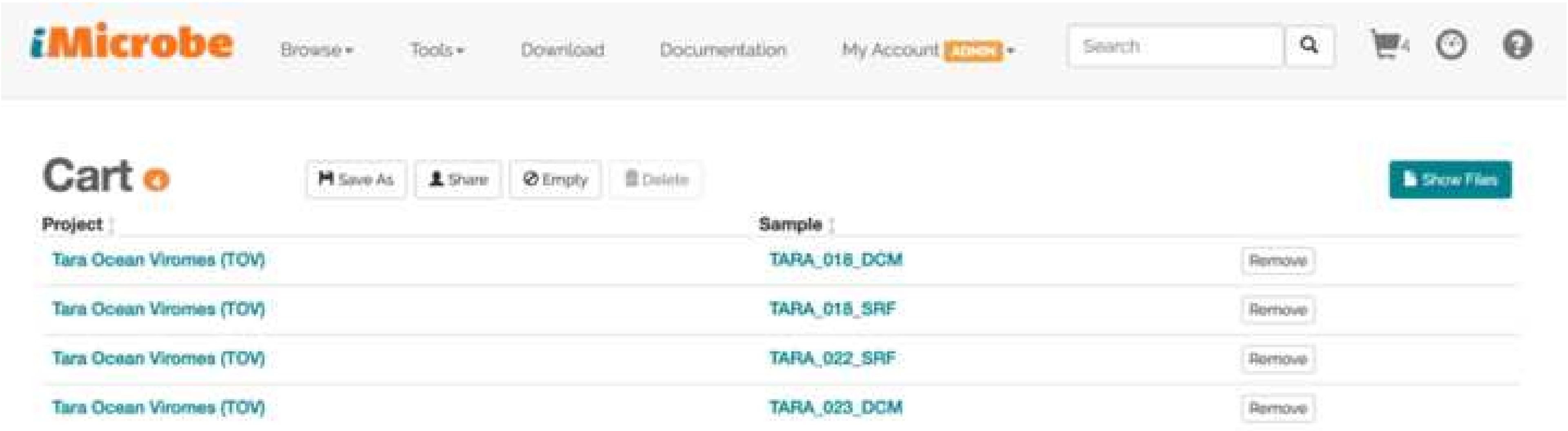

Click here to download Figure figure_4.png

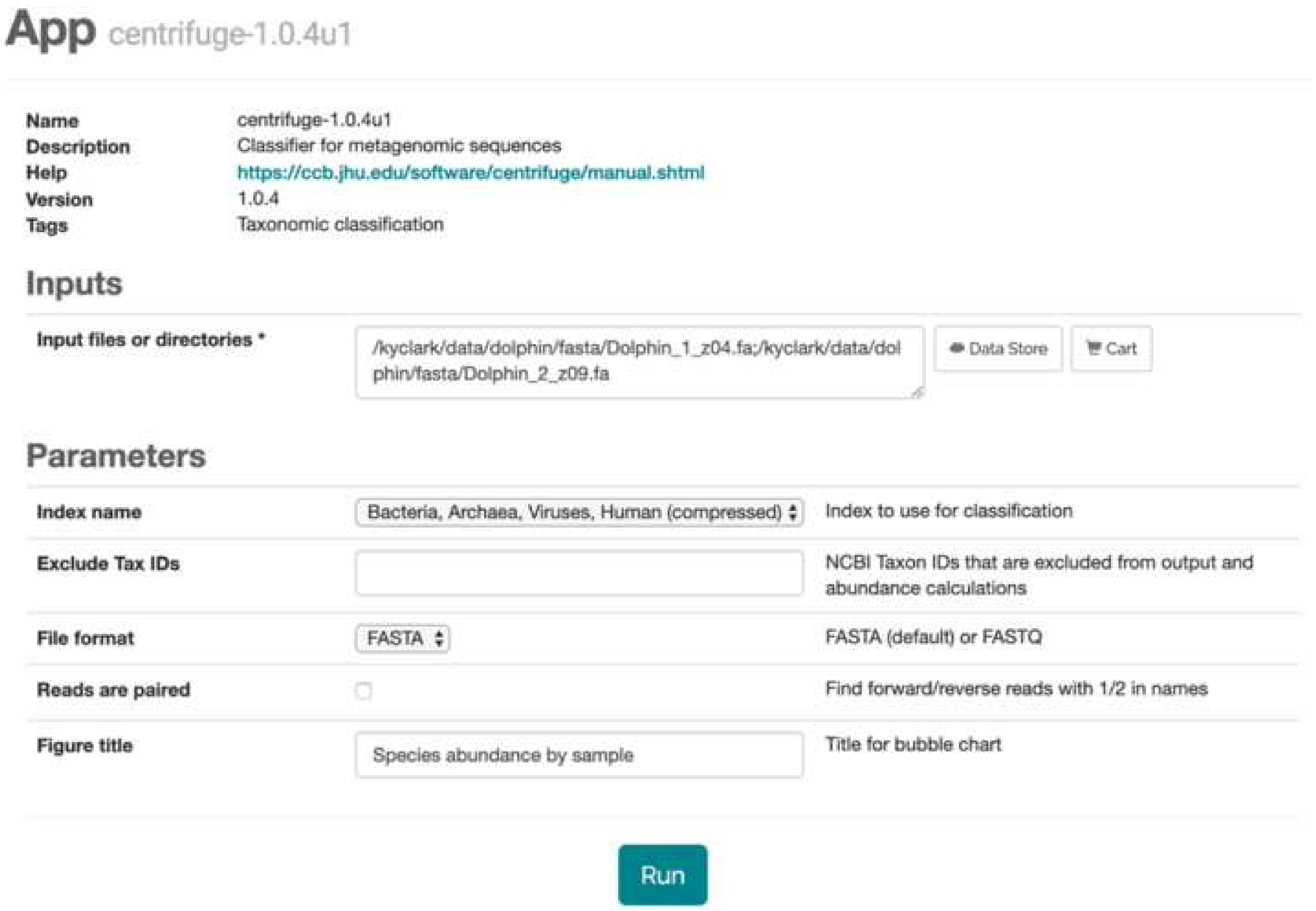

Click here to download Figure figure_5.png

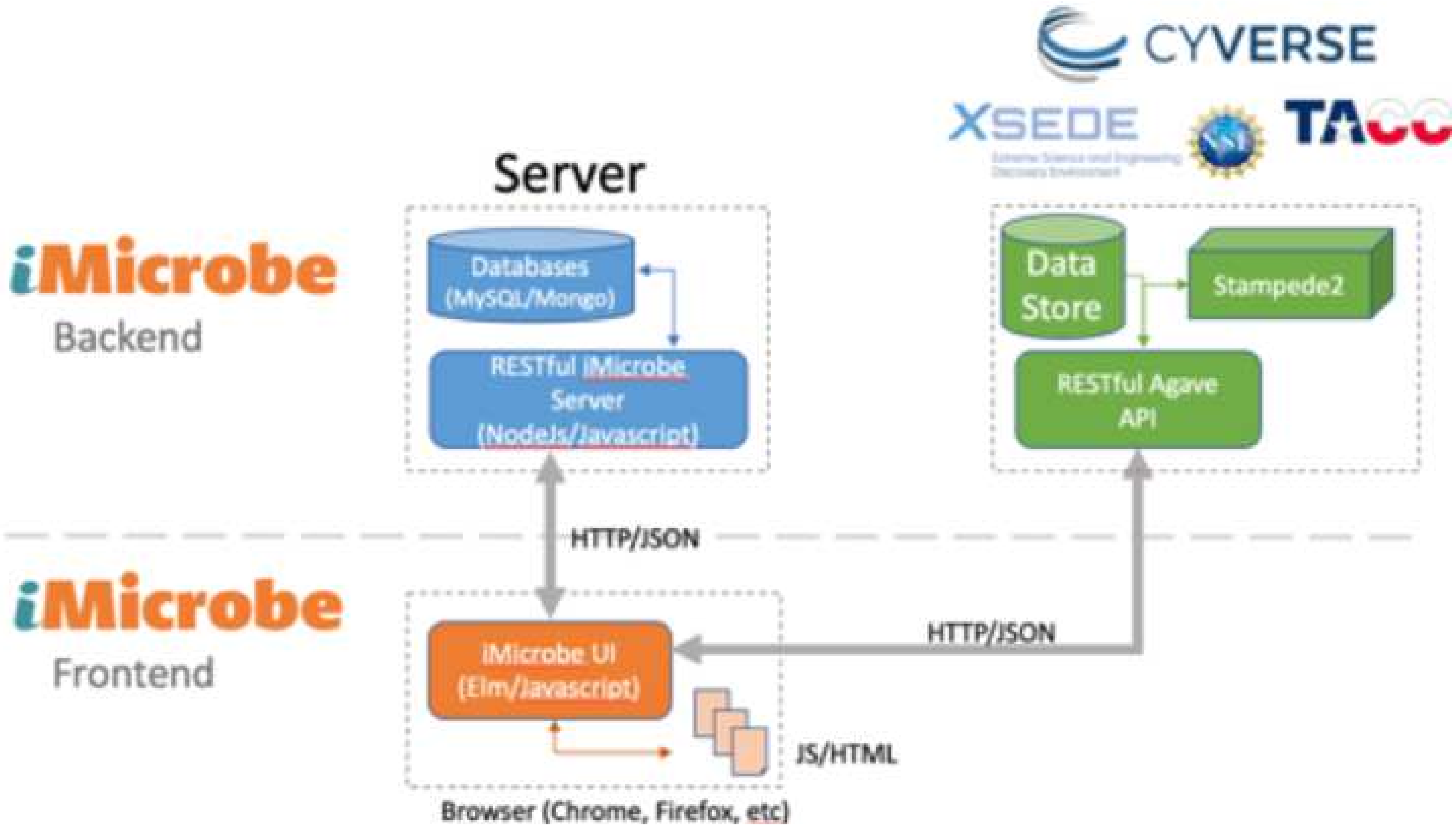

Click here to download Figure figure_6.png

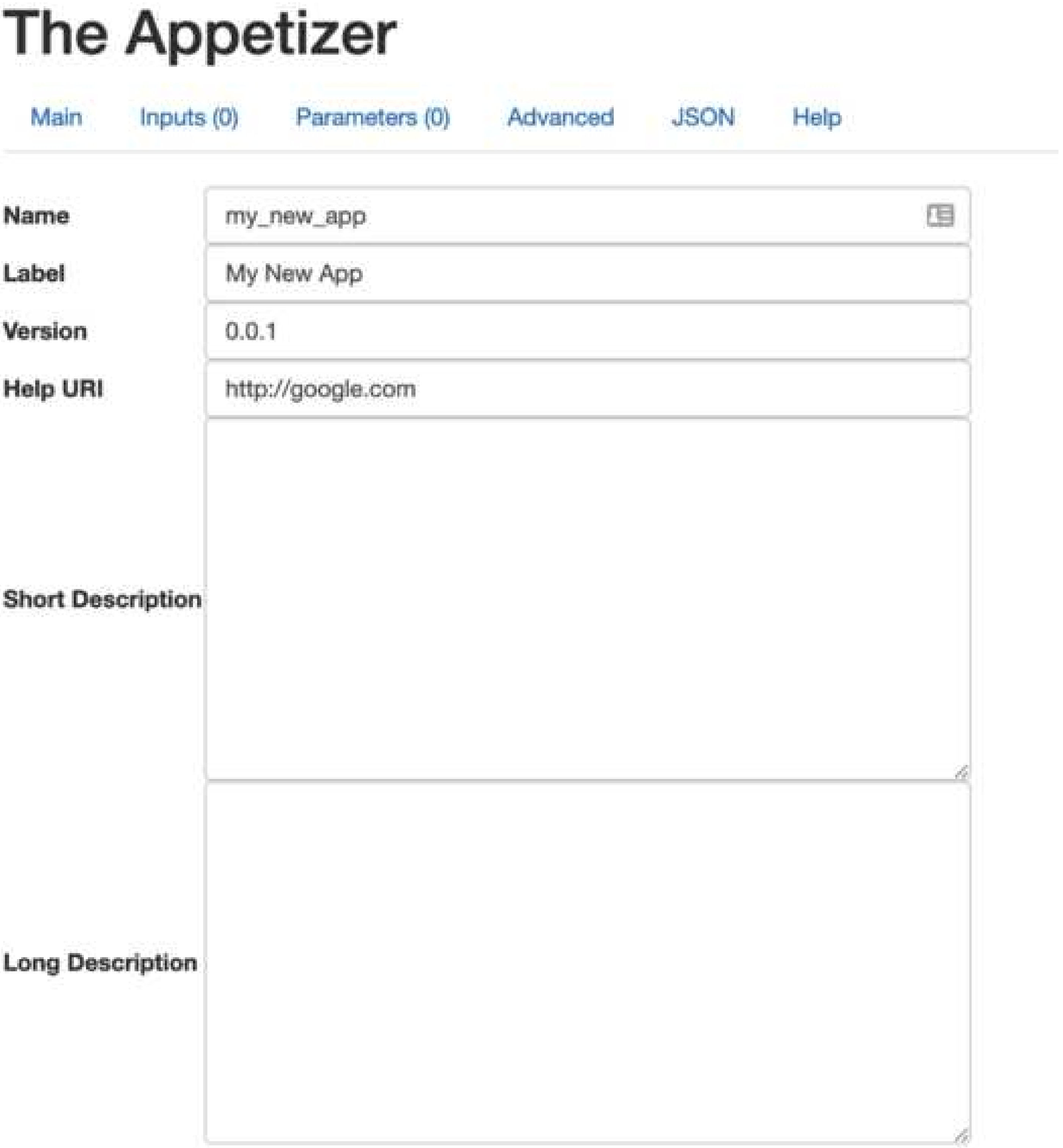

Supplement: giz083_GIGA-D-19-00107_Original_Submission [file giz083_giga-d-19-00107_original_submission.pdf]
