# Supplementary material for: iMicrobe: Tools and data-driven discovery platform for the microbiome sciences
Source: Gigascience. 2019 Jul 9;8(7):giz083. doi: 10.1093/gigascience/giz083 (PMC6615980; doi:10.1093/gigascience/giz083)
Supplement: giz083_GIGA-D-19-00107_Revision_1 [file giz083_giga-d-19-00107_revision_1.pdf]

GigaScience
iMicrobe: Tools and data-driven discovery platform for the microbiome sciences
--Manuscript Draft--

**Manuscript Number:**	GIGA-D-19-00107R1
**Full Title:**	iMicrobe: Tools and data-driven discovery platform for the microbiome sciences
**Article Type:**	Technical Note
**Funding Information:**	Gordon and Betty Moore Foundation (4491) — Dr Bonnie Hurwitz<br>National Science Foundation (1639588) — Mr Ken Youens-Clark<br>Simons Foundation (SCOPE) — Dr Bonnie Hurwitz
**Abstract:**	Background<br><br>Scientists have amassed a wealth of microbiome datasets making it possible to study microbes in biotic and abiotic systems on a population- or planetary-scale; however, this potential hasn't been fully realized given that the tools, data sets, and computation are available in diverse repositories and locations. To address this challenge, we developed iMicrobe.us, a community-driven microbiome data marketplace and tool exchange for users to integrate their own data and tools with those from the broader community.<br><br>Findings<br><br>The iMicrobe platform brings together analysis tools and microbiome data sets by leveraging National Science Foundation-supported cyberinfrastructure and computing resources from CyVerse, Agave, and XSEDE. The primary purpose of iMicrobe is to provide users with a freely available, web-based platform to (1) maintain and share project data, metadata, and analysis products, (2) search for related public datasets, and (3) use and publish bioinformatics tools that run on highly-scalable computing resources. Analysis tools are implemented in containers that encapsulate complex software dependencies and run on freely available XSEDE resources via the Agave API which can retrieve datasets from the CyVerse Data Store or any web-accessible location (e.g., FTP, HTTP).<br><br>Conclusions<br><br>iMicrobe promotes data integration, sharing, and community-driven tool development by making open source data and tools accessible to the research community in a web-based platform.
**Corresponding Author:**	Bonnie Hurwitz<br><br>UNITED STATES
**Corresponding Author Secondary Information:**	
**Corresponding Author's Institution:**	
**Corresponding Author's Secondary Institution:**	
**First Author:**	Ken Youens-Clark, MS
**First Author Secondary Information:**	
**Order of Authors:**	Ken Youens-Clark, MS<br>Matt Bomhoff, BS<br>Alise Ponsero, PhD

Powered by Editorial Manager® and ProduXion Manager® from Aries Systems Corporation

	Elisha Wood-Charlson, PhD
	Joshua Lynch, MS
	Illyoung Choi, MS
	John H Hartman, PhD
	Bonnie Hurwitz, PhD
**Order of Authors Secondary Information:**	
**Response to Reviewers:**	Response to Reviewers<br><br>Reviewer #1:<br>This manuscript describes iMicrobe, which is an online discovery and analysis platform for microbiome data. The manuscript is well written and easy to follow, and the resource is described in detail. The iMicrobe platform addresses an important need, allowing researchers to analyse their data and other publicly available datasets without the overhead of designing and implementing their own analysis workflows or pipelines. While other platforms already exist for this purpose, they tend to be relatively inflexible in terms of workflows provided, whereas iMicrobe offers a range of user-selectable analysis components. Provision of an additional microbiome analysis platform is also very positive for the research community, as microbiome research is a broad and complex field requiring a diversity of approaches, with no single tool or platform able to address every analysis need.<br><br>The iMicrobe platform is commendable in a number of its aims. These include i) emphasis on contextual sample metadata, ii) recording of analysis provenance, iii) containerisation of tools / analysis components to make them interoperable and to run on diverse compute environments, and iv) in providing users with access to super-compute resources by leveraging CyVerse and XSEDE. Nevertheless, I have some reservations about some of the finer details described in the manuscript that should be clarified or resolved prior to publication.<br><br>Response: We are grateful for your careful review and kind words about the iMicrobe platform. The points you make on the functionality and utility are exactly in line with what we hope to provide to the community.<br><br>In terms of data sets that can be analysed using the resource, there does not seem to be any mechanism for users to submit their sequencing data into a publicly available archive. Access to this data is vital so that data sets can be re-used, re-analysed and results compared for the benefit of the scientific community. Several of the major microbiome analysis platforms, including QIITA, MGnify and MG-RAST already offer this facility, brokering data into ENA. The iMicrobe developers should consider adding this option.<br><br>Response: We agree with the reviewer about the importance of being able to not only manage data in iMicrobe, but also submit to data repositories. We recently became a data broker for ENA and added functionality to the iMicrobe Data Dashboard to submit data to the European Nucleotide Archive (ENA). We added the description of this new feature in the manuscript as such:<br><br>“Additionally, iMicrobe is a data broker for the European Nucleotide Archive (ENA). Users can upload sequence files, enter project/sample metadata, and publish to ENA, all within the iMicrobe interface. iMicrobe validates the data/metadata and suggests additions or corrections but does not enforce any standards. This feature is available on request. A future version of the site will include this and conform to community standards.”<br><br>On page 6, the authors highlight the ability to search data sets by metadata. This is a very useful facility. However, the web site would benefit greatly from pagination. At present, searching for a widely populated attribute such as 'location:depth' returns a single page with thousands of samples listed, which is unwieldy.<br><br>Response: We appreciate and agree with this suggestion. We added this suggestion to our list of planned site improvements. We also added in a sentence in the manuscript

Powered by Editorial Manager® and ProduXion Manager® from Aries Systems Corporation

describing the feedback button on our site. All feedback is sent to a Basecamp project site, where bug fixes and new feature suggestions are discussed and prioritized.

Also on page 6, the authors state 'Samples are described in detail', but it is not clear where these descriptions are coming from. Are these provided by the original data submitters or are they curated by iMicrobe? Do projects comply with the MIxS community standards in terms of associated information; are the annotation fields and units used in line with the checklists for metagenome and metatranscriptome data?

Response: We thank the reviewer for raising these important questions. Many of the datasets included in iMicrobe to date were inherited from the CAMERA project. Unfortunately, these historical data were not curated by CAMERA or the original data providers and do not conform to MiXS standards. We are currently working on a project called Planet Microbe that will provide a new model for interoperable and standardized data for large-scale ocean expeditions (e.g. Tara, Malaspina, GOS, and Ocean Sampling Day). Moreover, we will add tools that allow the user to conform to these community-defined standards and submit data to ENA based on these standards. The idea is to harmonize historical datasets, and then provide a utility for users to continue to submit “gold-standard” data to promote data interoperability and re-use. This resource will be available for the ocean science community, but will be a model for other communities going forward.

We added the following sentence under the Metadata Search Section:

“Data sets included in the metadata search were inherited from the CAMERA project and were not thoroughly curated by CAMERA or the original data providers and do not conform to current community standards. As such, discrepancies may exist and users can contact original data providers and the iMicrobe developers via the website to address problems.”

Furthermore, who is the owner of the annotation (iMicrobe or the original data submitter?) and who should be contacted or is authorised to make changes or updates (for example, to correct an attribute that has been misannotated)?

Response: The data and the annotations belong to the original data submitter. To date, discrepancies have been reported directly to iMicrobe via our feedback page (https://www.imicrobe.us/#/contact), and our developers have corrected the mistakes. We clarified this point in the paper in the above point about data inherited from CAMERA.

On page 10, the authors state that 'any data set with a web link are accessible and computable in iMicrobe'. How realistic is this statement and does it need some kind of caveat for excessively large data sets? For example, if a user was to provide a link to the Tara Oceans project, which represents many Tb of sequencing effort, how feasible would it be to assemble and annotate the complete dataset, and what would be the kind of timescale?

Response: We thank the reviewer for this very important comment. Indeed, the Agave API can only retrieve reasonably sized data sets available over the Internet, and may fail for very large scale datasets such as the complete Tara ocean dataset. Moreover, iMicrobe uses resources available via TACC where compute jobs are typically limited to 24-48 hours. This is indeed a limit in term of computation power available through iMicrobe. We updated the main text to make this point clearer and added warning messages to the interface to inform the user on this limitation as such:

“Reasonably sized data sets that are available via a web-link (FTP or HTTP) are accessible and computable in iMicrobe if the analysis can be completed within a standard 24-48-hour window.”

On pages 12-15, the authors compare iMicrobe to other major publicly available microbiome analysis platforms and highlight the modular and configurable nature of the analyses available in iMicrobe. However, the manuscript does not mention the volume of data available via these resources (eg, 64,000 publicly available datasets in MG-RAST, 160,000 in MGnify, 250,000 in QIITA) and how this compares to iMicrobe (~6k).

	I think this is important to mention for fairness, since one of the benefits of using a standardised analysis pipeline is that it allows high-throughput, thus there tends to be a configurable analysis component/throughput trade-off. The authors might also want to discuss analysis of different kingdoms by the different platforms (for example, IMG/MG provide viral taxonomic analysis; MGnify, QIITE and MG-RAST do not). The statement 'Metadata searches in MGnify include temperature, depth and a few other fields', is also incorrect, as MGnify has over a hundred searchable metadata fields, which can be returned via the API (although metadata completeness depends on user submission and hence varies between projects).<br><br>Response: We thank the reviewer for these very important points. We agree with the reviewer on these points and updated the main text to provide a fairer comparison of the platforms:<br><br>“”<br><br>In Table 1, the authors list the apps that are currently available in iMicrobe, and this list will hopefully grow over time. The authors should comment on why (at least some) of these components were chosen - are they the best in breed in terms or performance, or were they chosen for more prosaic reasons, such as ease of containerisation?<br><br>Response: We thank the reviewer for this question. We do hope to increase the number of apps available through iMicrobe, especially by involving the research community for the inclusion of their most used tools and pipelines and by leveraging a tool we developed called The Appetizer.<br>The initial App development focused on tools and pipelines for read-based analytics, or community-specific tools or pipelines (such as the iVirus apps) that were not available in other platforms. We updated the manuscript to include this description:<br><br>“The current selection of apps provide additional read-based analytics not currently available on other platforms and specialized pipelines from collaborators. Future development will also include community-defined pipelines that are made available by major data repositories, such as ENA, to allow users to harmonize their data with other public data sources in a consistent manner.”<br><br>Along these lines, I have concerns relating to some of the Centrifuge taxonomic analysis results available on the site, in that many samples appear to have Homo sapiens as the most abundant source of sequencing reads. In some cases, such as human host-associated samples, this could represent host contamination and may therefore be reasonable. However, reads classified as human are also the majority in various environmental samples (Viral Stromatolite Metagenome; Microbial community gene expression across a productivity gradient of the Amazon River plume; Botany Bay Metagenomes, etc). Is this really contamination or is it a spurious result from Centrifuge?<br><br>Response: Thank you for this comment. We have also noticed this issue with the Centrifuge taxonomic annotation tool and are currently investigating it. For another paper, we are developing apps (and containers) for all k-mer based taxonomic classifiers and comparing them side-by-side using synthetic and biological datasets of known composition. This work will help to define issues in classification that can be propagated back to the site to correct any issues. The paper is currently in review and edits are due back by the end of this month, so this work will be complete in the next month.<br><br>Finally, for a number of datasets (for example Canine fecal metagenome, CAM_S_443), I was not able to see any downloadable files, protein analysis results or taxonomic analysis results on the site. Some kind of QC visualisation should be added to the site to help understand what lies behind this type of result (for example did the sequences not pass validation or did the analysis pipeline fail for some reason?)<br><br>We are aware of issues with the CAMERA data sets on iMicrobe. There is currently an effort by the National Microbiome Data Collaborative (NMDC) to curate these among many other historical data sets using community-defined standards. We hope to leverage these improved data sets on our site in the future.

Powered by Editorial Manager® and ProduXion Manager® from Aries Systems Corporation

Reviewer #2:
The authors present what appears to be a potent platform for analysis of microbiome sequencing data. The platform offers a some free compute and storage resources that appear to be enough for smaller studies. Within the platform, comparisons can be made to already publicly available data, or additional data provided by the user. Flexibility appears high, and the authors appear to expect most of the workflow design will be made by the end-users.

Online analysis platforms similar to iMicrobe have existed for several years, often using similar selling points, which typically include e.g. scaling to large analyses utilizing powerful compute resources, using or integrating with large datasets produced by others, etc. Some of the recurrent issues with online analysis platforms include the need to upload substantial amounts of data to an online server, the reliability and expected lifetime of the platform, and often the limited amount of flexibility in analyses. The first item is hard to work around, so I won't spend any time on that. It appears iMicrobe deals with the latter by essentially offering a web-based interface to standard command-line bioinformatics applications packaged in Singularity containers, leaving the workflow planning and execution to the end-users. I feel there is too little space in the article devoted to explaining how to connect the output from one app to the input of another and generally how to implement complete analysis workflows on the platform. There are some references to protocols.io, but I think it would benefit the article to have some more detail about this topic included.

Response: We thank the reviewer for a careful review of our work. We agree with the reviewer and think that the implementation of more complex workflows where the output of one app could be connected to a second app is an important feature. This constitutes our main future direction for the platform. We added this point in the discussion as such:

“Currently workflows are implemented inside an app. Future development plans include chaining apps together to create ad hoc workflows.”

The idea of an online analysis platform where data can be easily compared to other data from other groups is appealing, but for it to be truly useful it requires a large and active user base and reliable long-term funding. It is unclear to me from reading the paper what I can expect as an end-user about the long-term accessibility to the platform and data. Is there a designated place where end-users should direct questions, discussions, etc (other than maybe protocols.io)?

Response: We completely agree with the reviewer. Long-term usage and availability of the platform is one of the most critical aspect of this kind of project. Given lessons learned from the CAMERA project, we are highly aware of the consequences of the long-term availability of the ressources. We have therefore made several steps to allow reusability of this project by other communities. Specifically, we are currently working on submitting all CAMERA datasets to ENA that are not currently available in NCBI/ENA. Moreover, the tools developed for iMicrobe are available as containers for the community to reuse either by building locally from the recipes available in the public Hurwitz Lab GitHub repos (https://github.com/hurwitzlab), downloading them from ftp.imicrobe.us, or using directly on the Stampede2 cluster. We updated manuscript to make this critical point clear as follows:

“To ensure the long-term availability of the apps, we provide recipes to build the Singularity containers in the GitHub repository for each tool as well as the containers themselves on the iMicrobe FTP site and direct access to the containers in a public directory on the Stampede2 cluster.”

We agree with the reviewer that a place for user discussion and comments is needed. To this end, we added an additional sentence in the manuscript to say that this feature is available in the iMicrobe group at protocols.io.

“Through protocols.io, scientists can also create and share their own protocols and/or groups in the microbiome sciences as well as engage in discussions.”

Powered by Editorial Manager® and ProduXion Manager® from Aries Systems Corporation

To me, one of the weaker points of the article is the motivation for the existence of another online analysis platform. Thus, the section with comparisons to other online platforms for microbiome dataset analysis is very important. The idea of a comparison section is so that users familiar with any of the mentioned services can compare the basic similarities/differences more easily. However, I feel the descriptions are fairly thin and doesn't provide enough detail to produce any meaningful comparisons. Maybe the information be summarized in a feature table that compares all tools simultaneously rather than just providing plain text paragraphs doing pairwise comparisons to iMicrobe?

Response: We thank the reviewer for this comment, we agree with these points and added a two tables summarizing the comparison as such:

Table 2: Comparison of metagenomic platforms' cyberinfrastructure capabilities.
KBaseMGnifyMG-RASTIMG/MQIITAiMicrobe
Create apps✓ ✓
Run apps at will✓ ✓ ✓✓
Upload private data✓ ✓✓*✓✓
Share private data ✓
Search public data✓✓ ✓✓✓
* for users of JGI sequencing services

Table 3: Comparison of metagenomics platforms' app capabilities.
KBaseMGnifyMG-RASTIMG/MQIITAiMicrobe
GeneralQC✓✓✓✓✓✓
GenomicsAssembly✓ ✓
Gene calling✓ ✓
Gene annotation✓ ✓
Metabolic modeling✓
Sequence analysis✓ ✓
Comparative genomics✓
Metagenomics (assembly-based analysis)Assembly✓ ✓ ✓
Gene calling ✓✓ ✓
Gene annotation ✓✓ ✓
Taxonomic classification of contigs ✓✓ ✓
Protein clustering ✓ ✓
Read mapping to contigs or other reference✓
Metagenomics (read-based analysis)Read taxonomic classification✓✓✓ ✓
Read ORF prediction ✓✓ ✓
Read functional annotation ✓✓ ✓
Read clustering ✓
AmpliconOTUs & taxonomic lineage ✓✓✓✓✓

The mention of limitations of QIIME2 to 16S data in the section on QIITA appears incorrect. As far as I can tell from QIIME2's homepage there exists plugins (MetaPhlAn2 and SHOGUN) to also analyze shotgun data.

Response: We thank the reviewer for this correction, we updated the text accordingly as such:

"QIITA [33] allows users to upload and analyze maker gene data sets (16S rRNA, ITS, and 18S rRNA), metagenomic, and metabolomic datasets using QIIME2 and Global Natural Product Social Molecular Networking (GNPS) tools. Using these tools, users can examine the taxonomic composition of marker gene and whole genome shotgun (WGS) metagenomic datasets, and perform automated molecular network analysis and crowdsourced MS/MS spectrum curation for metabolomic datasets. However, QIIME2 does not currently support upstream analyses for WGS datasets or analyses beyond taxonomic annotation and analysis. iMicorbe currently hosts a 16S rRNA pipeline and clustering tool [34], in addition to many other general purpose tools for QC, trimming, read assembly, pairwise sequence alignment, and gene functional annotation, among others."

I would like to see a more in depth explanation of the long-term plan to handle app

Powered by Editorial Manager® and ProduXion Manager® from Aries Systems Corporation

	versioning. In a couple of years, there will have been a multitude of different tools and a large amount of newer versions of the same tools. How will that be managed in the interface? For now, it appears it is mostly a long list of all available applications, which will most likely become cumbersome or hard to browse over time.<br><br>Response: We agree with the reviewer, this is a very important point. We started implementing terms to the tools describing their operation, data input and output according to the EDAM ontology of bioinformatic operations. In the future, when the number of apps grow larger, this aims will allow the user to retrieve apps by their function, input data type or output. We added this text to the manuscript:<br><br>"Currently users can find tools by using standardized tags or app names and descriptions. As the app lists grows, we plan to offer additional ways to organize and search for apps, including by application versions."
Additional Information:	
Question	Response
Are you submitting this manuscript to a special series or article collection?	No
**Experimental design and statistics**<br><br>Full details of the experimental design and statistical methods used should be given in the Methods section, as detailed in our Minimum Standards Reporting Checklist. Information essential to interpreting the data presented should be made available in the figure legends.<br><br>Have you included all the information requested in your manuscript?	Yes
**Resources**<br><br>A description of all resources used, including antibodies, cell lines, animals and software tools, with enough information to allow them to be uniquely identified, should be included in the Methods section. Authors are strongly encouraged to cite Research Resource Identifiers (RRIDs) for antibodies, model organisms and tools, where possible.<br><br>Have you included the information requested as detailed in our Minimum Standards Reporting Checklist?	Yes
**Availability of data and materials**	Yes

All datasets and code on which the conclusions of the paper rely must be either included in your submission or deposited in publicly available repositories (where available and ethically appropriate), referencing such data using a unique identifier in the references and in the “Availability of Data and Materials” section of your manuscript.<br><br>Have you have met the above requirement as detailed in our Minimum Standards Reporting Checklist?	

Powered by Editorial Manager® and ProduXion Manager® from Aries Systems Corporation

Manuscript with DOI

Click here to view linked References

Click here to access/download;Manuscript;imicrobe_gigascience_technical_n

Title: iMicrobe: Tools and data-driven discovery platform for the microbiome sciences

Authors: Ken Youens-Clark[1], Matt Bomhoff[1], Alise Ponsero[1], Elisha M. Wood-Charlson[2], Joshua Lynch[1], Illyoung Choi[3], John H. Hartman[3], and Bonnie L. Hurwitz[1,4]

[1] Department of Biosystems Engineering, University of Arizona, Tucson, AZ, USA.

[2] Environmental Genomics and Systems Biology Division, E.O. Lawrence Berkeley National Laboratory, Berkeley, California, USA.

[3] Department of Computer Science, University of Arizona, Tucson, Arizona, USA.

[4] BIO5 Institute, University of Arizona, Tucson, Arizona, USA.

Abstract

Background: Scientists have amassed a wealth of microbiome datasets making it possible to study microbes in biotic and abiotic systems on a population- or planetary-scale; however, this potential hasn’t been fully realized given that the tools, data sets, and computation are available in diverse repositories and locations. To address this challenge, we developed iMicrobe.us, a

community-driven microbiome data marketplace and tool exchange for users to integrate their own data and tools with those from the broader community. **Findings:** The iMicrobe platform brings together analysis tools and microbiome data sets by leveraging National Science Foundation-supported cyberinfrastructure and computing resources from CyVerse, Agave, and XSEDE. The primary purpose of iMicrobe is to provide users with a freely available, web-based platform to (1) maintain and share project data, metadata, and analysis products, (2) search for related public datasets, and (3) use and publish bioinformatics tools that run on highly-scalable computing resources. Analysis tools are implemented in containers that encapsulate complex software dependencies and run on freely available XSEDE resources via the Agave API which can retrieve datasets from the CyVerse Data Store or any web-accessible location (e.g., FTP, HTTP). **Conclusions:** iMicrobe promotes data integration, sharing, and community-driven tool development by making open source data and tools accessible to the research community in a web-based platform.

Keywords

Cyberinfrastructure, cloud computing, bioinformatics, metagenomics.

Introduction

iMicrobe is a platform that connects researchers' own data to published, curated, microbial metagenomic datasets and high-performance computing methods for their analysis [1]. In the last decade, the cost of sequencing has decreased at a rate far outpacing Moore's law, leading to a rapid increase in the number and size biological datasets [2]. Researchers now have access to an

unprecedented scale and variety of data ranging from large-scale ‘omics data to streaming data from sensors. Biologists increasingly need the power and storage of high-performance computing (HPC) clusters to perform analyses; however, most biologists have limited or no access to these resources and often have to run analyses on their own personal computers.

To address the growing need for HPC in computational biology, the National Science Foundation (NSF) has funded shared cyberinfrastructure resources like XSEDE [3] and Stampede2 [4], an 18-petaflop supercomputer at the Texas Advanced Computing Center (TACC) at the University of Texas at Austin. Developers at TACC have created Agave [5], a REST [6] Application Program Interface (API), to interact with Stampede2’s resources including creating and editing apps, scheduling, and monitoring jobs, and viewing and retrieving the results of analysis jobs. The iMicrobe website makes use of the Agave API [7] to create a web-based portal to the CyVerse [8] DataStore [9] and HPC resources such as the Stampede2 HPC via a free CyVerse account.

iMicrobe users can use their web browser to search public metagenomics datasets like CAMERA [10], save data to a cart, upload their own personal data sets, and run over 30 analysis tools on both private and public data sets using free compute on Stampede2 (Fig. 1). All data in iMicrobe is also available via FTP [11] or from the CyVerse Data Store via iRODS [12] (command line) or the CyVerse Data Commons [13] (web browser). The Agave API also allows for direct command-line access to data and pipelines via the CyVerse SDK [14]. Users can login directly to Stampede2 and use iMicrobe’s analysis tools in their Singularity [15] containers or build containers from source to use on their own computing resources. Finally, developers can use the Agave API to create novel tools that can be integrated into iMicrobe.

Figure 1: *iMicrobe's architecture allows for the integration of datasets hosted by iMicrobe which can be placed into the data cart, those private to the user, and others publicly accessible on the Internet. Analyses created by iMicrobe or other developers run on Stampede2, and the results go into the users' home directory in the CyVerse Data Store.*

Findings

iMicrobe leverages CyVerse cyberinfrastructure and XSEDE supercomputing resources

Security & Trustworthiness. iMicrobe leverages the CyVerse cyberinfrastructure (CI) to provide services to users including the OAuth2 authentication system [16] for secure single sign-on between iMicrobe and all CyVerse services as well as the CyVerse Data Store for storing, sharing, and distributing large amounts of data and analyses. Users can also access high-performance computing systems such as XSEDE's Stampede2 cluster to execute analyses that originate from iMicrobe apps.

Usability & Data Storage. iMicrobe uses the CyVerse Data Store for data sets and analysis results. CyVerse Data Store offers solutions to many contemporary data storage needs in the age of large, distributed, digital data. CyVerse's cloud-based data storage is optimized for large data, is free to most scientific researchers, is accessible through multiple interfaces, and leaves access control in the hands of the data owners. The Data Store provides research scientists, research groups, and research organizations with private, shared, or public storage allocations primarily for use within the CyVerse CI. The CyVerse Data Commons houses public data within the Data Store for use by the research community either within or outside CyVerse CI. The Data Store

offers reliable, secure storage for datasets of any size that are actively being used for both research and/or education purposes. Data and metadata in the Data Store are stored in a high-performance storage resource that has built-in redundancy and is continuously monitored for security and failure. The Data Store is synchronously backed up at both the University of Arizona in Tucson, Arizona, and at the Texas Advanced Computing Center in Austin, Texas. All users initially get 100GB of storage but can request additional allocations through CyVerse [17]. Private user data is stored in a user's home folder ("/iplant/home/$user," where $user is a CyVerse username), and will be accessible through a view in iMicrobe where files can be uploaded, deleted, shared, or modified. Users can share data that they own with other registered CyVerse users. Project leaders can request larger allocations for collaborative projects. Project leaders can also request a Community Data Folder that will be made public [18]. Community Data folders are housed in the "iplant/homes/shared" directory and are visible via iMicrobe under "Community Data". Data policies associated with iMicrobe are in sync with CyVerse policies. Additionally, iMicrobe is a data broker for the European Nucleotide Archive (ENA). Users can upload sequence files, enter project/sample metadata, and publish to ENA, all within the iMicrobe interface. iMicrobe validates the data/metadata and suggests additions or corrections but does not enforce any standards. This feature is available on request. A future version of the site will include this and conform to community standards.

Provenance & Reproducibility. iMicrobe data and analysis provenance both primary data, derived files, and analyses are tracked in CyVerse by keeping all files in the analysis directory along with data products and a log file to maintain information about the job and parameters that were run. CyVerse also maintains a job history to allow researchers to track and reproduce

experiments. In iMicrobe, data provenance is imperative given that data are derived from diverse data stores that have varied levels of curation and versioning.

General Search

The upper-right corner of every page on iMicrobe has a search box that will perform a simple query over text in our databases related to projects, investigators, samples, taxonomy, and proteins. For instance, a search for "obese" finds 28 hits including a publication, two projects, and the 25 samples from those two projects.

Metadata search

The samples in iMicrobe can be searched by their metadata or "data about the data". Samples are described in detail by over 200 attributes such as type (artificial metagenome, isolate, metagenome, metatranscriptome, transcriptome), biome (sewage, soil, deep chlorophyll max, acid mine drainage), depth, dissolved oxygen, latitude/longitude, salinity, host organism, chlorophyll, and more. The values for these can be numeric or character values such as a measurement of depth in meters or "Synechococcus" for the host organism. In total, there are over 140K descriptors for our samples, and users can specify an unlimited number of terms using the sample metadata search tool [19]. Data sets included in the metadata search were inherited from the CAMERA project and were not thoroughly curated by CAMERA or the original data providers and do not conform to current community standards. As such, discrepancies may exist and users can contact original data providers and the iMicrobe developers via the website to address problems.

Figure 2: *The sample metadata search allows users to search over both text and numeric values in any combination. Each additional search attribute updates the discovered samples immediately and restricts the next attributes the user can use to those found in the sample subset. Valid search values are displayed as multi-select boxes for strings and min/max values for numbers.*

Each time a user selects a new attribute such as “phosphate,” the interface determines if the attribute is a character or numeric data. For character data, the user is presented with a multi-select list if the number of choices is reasonable to aid in selecting the correct strings. For numbers, minimum and maximum fields are shown with placeholders indicating the current min/max values from the database for the current subset of records shown. For example, if “phosphate” is selected as the first attribute, the min/max values are 0.01/3070, respectively, but if the user first selects a “Longhurst Province” of “ARAB” (NW Arabian Upwelling Province) then the “phosphate” values will be only for those samples found in that province which range from 36 to 50. In this way, users may quickly winnow sample searches to those matching their exact criteria and place the results into the cart.

From the sample details page (e.g., https://www.imicrobe.us/#/samples/5189), users can view a sample’s location on Google Maps (if applicable), add the sample to the cart, view and download the associated data products, and examine the sample’s attributes, predicted proteins, and predicted taxonomic classifications.

Data Cart

Throughout the iMicrobe site, users can add an unlimited number of samples of interest to the data cart. Samples can be easily removed, or the cart can be cleared entirely. Using the cart, users

can download the associated data products. Users can filter data products (files) by their associated types (e.g., reads, gene calls, predicted proteins, taxonomy classifications). Users can download data either from the CyVerse Data Store or the iMicrobe FTP site [11]. Contents of the cart can also be used as the input to several of iMicrobe's applications or "apps." Carts can be saved and shared with other users.

Figure 3: *The data cart holds any number of samples selected by the user. Using the cart view, users can select the associated data files or analysis products to download. Cart contents can also be used as input values for apps.*

Apps

iMicrobe currently hosts more than 30 self-contained applications ("apps") (Table 1) or analysis pipelines that take some input files and program parameters (Fig. 4), run to completion, and deposit the results into the users CyVerse Data Store home directory. Apps allow users to easily do quality control "QC" (Trim Galore, Trimmomatic), predict proteins (UProc) and genes (Prodigal, MetaGeneAnnotator, FragGeneScan), assemble contigs (Megahit, SOAPdenovo), assign taxonomy (Centrifuge), and cluster genomes (Mash, Libra, Fizkin) (Table 1). The current selection of apps provide additional read-based analytics not currently available on other platforms and specialized pipelines from collaborators. Future development will also include community-defined pipelines that are made available by major data repositories, such as ENA, to allow users to harmonize their data with other public data sources in a consistent manner. Currently users can find tools by using standardized tags or app names and descriptions. As the app lists grows, we plan to offer additional ways to organize and search for apps, including by application versions.

In order to view and run apps, the user must first create an account with CyVerse (https://user.cyverse.org). Accounts are free to create and allow iMicrobe to connect in a user’s data in CyVerse including novel datasets they may wish to analyze with an app or the results of running an analysis tool. Currently workflows are implemented inside an app. Future development plans include chaining apps together to create *ad hoc* workflows. To ensure the long-term availability of the apps, we provide recipes to build the Singularity containers in the GitHub repository for each tool as well as the containers themselves on the iMicrobe FTP site and direct access to the containers in a public directory on the Stampede2 cluster.

Table 1

Apps can allow for new tools to be more quickly disseminated to the community. Often the discovery of a newly published bioinformatics tool leads to the frustration of downloading and compiling source code, resolving dependencies, and fighting with conflicting versions of tools (e.g., gcc or Python/conda). When tools are released as self-contained packages, others can more quickly and easily test the tools on their own datasets. If community developers release tools as containers, they can more easily be made available as apps through Agave and iMicrobe.

Figure 4: *The app launch interface allows users to select input files and set parameters for the app. The input files may come from the users’ own Data Store, any publicly available data in the Data Store such as files associated with their data cart or iMicrobe sample files, or any other file available over FTP or HTTP. The Agave API will copy the input files to the compute node when the job is run.*

Making data and tools Findable, Accessible, Interoperable, and Reusable (FAIR)

iMicrobe is pursuing FAIR [20] (Findable, Accessible, Interoperable, and Reusable) principles as it relates to both data and computation. We strive to make data findable via searches; accessible via the CyVerse Data Store, iRODS or FTP; interoperable via common file formats; and reusable via open access. Likewise, we aim to make compute findable via our website, accessible via the Agave API, interoperable via Singularity containers, and reusable via open access. Just as common file formats like FASTA or GFF make data exchange simple, containers like Docker [21] and Singularity allow computational methods to be run on any system and promote reuse.

Reusable

Making data reusable via the dynamic metadata search in iMicrobe. An acute need in the microbiome community is to discover and integrate data in disparate data repositories to facilitate analyses. Specifically, primary and associated contextual metadata, as well as other data products (and their provenance), exist in diverse microbiome data repositories. To make data more discoverable, iMicrobe provides a dynamic search to find data sets based on structured and curated metadata. Data can be added to a shopping cart and analyzed using a variety of tools.

Making tools reusable by creating containers. As for computing resources, users can find iMicrobe apps on the iMicrobe website [22]. Most iMicrobe apps use Singularity containers which can be accessed directly from the Stampede2 file system under a shared iMicrobe directory. The recipes to build iMicrobe's Singularity containers are stored in GitHub

repositories (see Table 1), making it possible for users to build and use containers locally on their own datasets.

Accessible

Analyzing remote web-accessible datasets in iMicrobe. Because a much ‘omics data lives in diverse repositories globally, moving the data to a central location for processing can be problematic and inefficient. Still, scientists need to bring together diverse data sets to enable population-level or planetary-scale analyses that drive new knowledge and discovery. iMicrobe delivers a virtual framework for connecting web-accessible remote microbiome ‘omics data with private user data using the Agave API. Reasonably sized data sets that are available via a web-link (FTP or HTTP) are accessible and computable in iMicrobe if the analysis can be completed within a standard 24-48-hour window. iMicrobe provides ready access to the CAMERA data collection including reads, peptides, CDS, contigs, assemblies, annotations, as well as related projects derived from the sample and environmental data for 120+ microbiome projects, representing 1 TB of data. These data are hosted in the CyVerse Data Store (“/iplant/shared/imicrobe”) and are integrated into iMicrobe under "Community Data”. A user can analyze CAMERA data alongside their own personal data sets and also any other data set which the Agave API can retrieve from a public web address.

Interoperable

Containerizing tools and pipelines for making analyses interoperable on diverse compute platforms. Microbiome science methods and bioinformatics code are constantly evolving and can be cumbersome to install [23]. Moreover, users may not have access to the computing

resources required to run the tool on their data. To this end, iMicrobe converts tools into Singularity containers, packaged virtual machines that encapsulate the operating system, dependencies, and tool's code to ensure reproducibility and allow the code to run on any computational architecture including the Stampede2 HPC. Community developers can contribute tools by releasing containers to Biocontainers or Docker Hub. Currently, most iMicrobe apps are deployed at TACC Stampede2 using the Agave API; however, the containerized tools can theoretically be run on any computer resource including cloud resources such as Amazon Web Services (AWS) or Google Cloud Platform. The iMicrobe platform automatically creates a user interface to launch a container by using a JSON (JavaScript Object Notation) description of the app that encodes its inputs and parameters. The JSON also specifies hardware requirements (CPU and memory) to run the tool, e.g., on the default queue or a high-memory node at Stampede2. iMicrobe streamlines community-driven tool development and accessibility to a variety of tools in a simple web-based platform.

Running Analyses through the iMicrobe web-based platform. To run an analysis, users select an app from the "apps" listing; select data "inputs" from their own CyVerse Data Store, publicly available data in their shopping cart, or some publicly-accessible URL; select parameters; and launch the tool with the click of the "Run" button. Users can track the status of their jobs directly on the site and view results and interactive data visualizations. As with all files, users can share analysis results with collaborators. Provenance of primary data derived files, and analyses are tracked in CyVerse by keeping all files in the analysis directory, along with data products and a log file record about the job, including data sources, app versioning, and the parameters selected for that run. CyVerse also maintains the job history to allow researchers to track and reproduce other researcher's experiments.

Reusable

Virtual communities, protocols, and documentation for iMicrobe. Given the experimental nature of methods in microbiome research, iMicrobe fosters discussions about both molecular and computational protocols with an eye towards improving methods. iMicrobe partners with protocols.io [24], a method-centered collaborative platform, to provide guides and sample data sets for popular use cases in microbiome research. These methods are available through the iMicrobe virtual community [25]. Through protocols.io, scientists can also create and share their own protocols and/or groups in the microbiome sciences as well as engage in discussions. Users can also access documentation on using the iMicrobe website and protocols through the iMicrobe Gitbook [26]. Users are also encouraged to submit feedback directly to the site via the Feedback button. All bug fixes and feature suggestions are discussed within the development team and prioritized.

Comparison to Web-based Metagenomics Platforms

There are many tools for metagenomic analysis many of which are native desktop applications for Windows, Apple, or Linux operating systems. As iMicrobe is a web application focused on connecting remotely hosted data sets to large compute capacity, we have chosen several similar systems to which we compare. See tables 2 and 3 for a comparison of metagenomic platforms' cyberinfrastructure capabilities and app capabilities, respectively.

KBase

The Department of Energy's (DOE) Systems Biology Knowledgebase [27] is "an open-access bioinformatics software and data platform for analyzing plants, microbes, and their communities." KBase offers several dozen "apps" which can be organized into workflows called "narratives." To create a novel app, users must apply to for a KBase developer account and install the KBase Software Development Kit (SDK) and dependencies (Java 1.7, Python 2.7, NodeJS, Bower, Docker), and work with the KBase staff to integrate the app. Apps run on computer resources at Lawrence Berkeley National Lab (LBNL) or Argonne National Lab (ANL) with plans to expand to other DOE HPC or cloud resources. In contrast, developers who wish to create a CyVerse or iMicrobe app are simply required to put that app into a Singularity container and describe the inputs and parameters using a JSON document. As described below, web-based interfaces can be used to create the necessary JSON app definitions.

MGnify

MGnify [28] is metagenomics platform from the European Bioinformatics Institute (EBI). Users can publicly archive their data and receive a permanent accession which can be used to retrieve sequences and metadata via EBI's European Nucleotide Archive (ENA). After releasing data to ENA, MGnify integrates metagenomic datasets and may run one or more versions of their standard pipeline on datasets [29]. Metadata searches in MGnify include temperature, depth, biome, sequencing method, and a few other fields. In contrast, all metadata fields are available in iMicrobe via the sample/metadata search. Users are not able to run MGnify analysis pipelines directly but may submit a request to analyze private or public datasets. In contrast, iMicrobe

users can run apps (which include analysis pipelines) directly from the integrated app and job submission interface as described above.

MG-RAST

MG-RAST [30], or the metagenomics rapid annotation using subsystems technology server, makes it possible for users to upload raw metagenomic sequence data in FASTQ or FASTA format. Assessments of sequence quality and annotation with respect to multiple reference databases are performed automatically with minimal input from the user. Post-annotation analysis and visualization are also possible, directly through the web interface, or with tools like matR (metagenomic analysis tools for R) that use the MG-RAST API [31] to download. Similar to MGnify, a single comprehensive analysis pipeline is applied to all user datasets. By contrast, iMicrobe users can upload any data type relevant to any app and run them in a modular fashion and adjust the parameters to be more specific to their data and research question.

IMG/M

The Integrated Microbial Genomes & Microbiomes “IMG/M” [32] is a service that supports “the annotation, analysis, and distribution of microbial genome and microbiome datasets sequenced at DOE's Joint Genome Institute (JGI).” Users can use IMG/M for “annotation, analysis, and distribution of their own genome and microbiome datasets” but cannot create and distribute novel tools for community access. Like MGnify and MG-RAST, IMG/M provides uses with a single comprehensive pipeline for analysis. As described previously, iMicrobe provides users with apps that are self-contained and can be run in any order, with user-defined parameters.

QIITA

QIITA [33] allows users to upload and analyze maker gene data sets (16S rRNA, ITS, and 18S rRNA), metagenomic, and metabolomic datasets using QIIME2 and Global Natural Product Social Molecular Networking (GNPS) tools. Using these tools, users can examine the taxonomic composition of marker gene and whole genome shotgun (WGS) metagenomic datasets, and perform automated molecular network analysis and crowdsourced MS/MS spectrum curation for metabolomic datasets. However, QIIME2 does not currently support upstream analyses for WGS datasets or analyses beyond taxonomic annotation and analysis. iMicrobe currently hosts a 16S rRNA pipeline and clustering tool [34], in addition to many other general purpose tools for QC, trimming, read assembly, pairwise sequence alignment, and gene functional annotation, among others.

Table 2

Table 3

Methods

Architecture

iMicrobe employs a common web architecture of dividing the “front-end” user interface from a “back-end” API (application programming interface; Fig. 5). The front-end is written in Elm [35], a purely functional language similar to Haskell [36]. Elm code compiles to the JavaScript that the browser runs to fetch data from the API and format it for the user. The API, written in Node/JS [37], handles requests for data from MySQL [38] and MongoDB [39] and returns data

in JSON [40] format. For instance, the projects listing [41] loads in the browser then makes a request to the API [42] for its data and dynamically creates the table listing. This architecture, while more complicated, leads to better user experience as pages load quickly and then perform longer-running tasks such as requesting and formatting the data. In addition, the data becomes available to third parties who may prefer to use the API to get structured, machine-readable data (JSON) rather than HTML.

Figure 5: *The iMicrobe user interface is comprised of a back-end written in Node/JS that talks to MySQL and MongoDB databases to deliver JSON to a front-end written in Elm which also communicates with the Agave API for the computing resources of CyVerse Data Store and TACC's Stampede2 HPC cluster.*

Databases

All metadata from investigators to projects to samples and sample attributes are stored in a MySQL relational database primarily using the InnoDB table engine to maintain referential integrity of the data. The MyISAM engine is used for the "quick search" table as that employs a "FULLTEXT" index to handle text searching [43]. Sample attributes are a mix of both text and numeric data, e.g., a Longhurst code like "GUIN" or a chlorophyll measurement like "0.82." One disadvantage of a traditional relational database management systems (RDBMS) is that these values must be stored as text. In order to support the mix of numeric and string searching required for the sample metadata search [19], the sample metadata is denormalized and mirrored into a MongoDB in order to take advantage of a much richer search engine. In MongoDB, those values that appear to be numeric are coerced as such enabling range-based queries such as samples with chlorophyll between 5 and 10 in addition to text-based searches. Users may

provide lower and/or upper bounds for numeric queries and additionally mix restrictions on textual values until a suitable subset of samples has been found.

Stampede2

iMicrobe provides access to both large datasets and the tools and computing resources to analyze them through apps which are run on the Stampede2 cluster. iMicrobe uses the Agave API to launch the app and copy input data files from the CyVerse Data Store or any other web-accessible location (e.g., FTP or HTTP) to Stampede2. Users can also request a personal account on Stampede2 in order to directly use the system and all iMicrobe's databases (e.g., iMicrobe/Ohana BLAST, Centrifuge, UProc) and app containers.

Docker/Singularity

Almost every app on iMicrobe [22] is deployed in a Singularity container in order to encapsulate a base operating system and all the dependencies of the included tools. Because of security issues when running Docker containers, TACC only allows Singularity containers. If a tool is available as a Docker container, e.g., through Docker Hub [44], it is a simple matter to build a Singularity container. By using containers, users are never required to install any software locally with the exception of Singularity itself. Every Singularity-based app in iMicrobe has a GitHub repository with the definitions and instructions to create the Singularity containers. Users may choose to extend these definitions as well as modify the code to customize iMicrobe tools.

Biocontainers

Much work has been done in various scientific communities to package and distribute tools using Docker using Biocontainers [45]. Stampede2 has a directory (/work/projects/singularity/TACC/biocontainers) containing over 7,000 such containers which can be leveraged by researchers if they have the resources to run these. For instance, iMicrobe has incorporated the Trim Galore Biocontainer (for quality checking of reads) [46]. A push by other community developers to help create more Docker/Singularity containers and describe them to Agave could greatly increase the number of tools available via Agave and platforms like iMicrobe.

The Appetizer

If an app is written such that it has a Singularity container and can be run in a batch mode (that is, given arguments and run to completion unattended by a user), it can be made to run on the Stampede2 system via the Agave API by describing the app's required resources, inputs, and parameters in a JSON file [47]. Both Agave and iMicrobe offer web-based interfaces to create this JSON definition. iMicrobe's is called "The Appetizer" [48]. In this way, the creation of apps is not limited to the developers of iMicrobe. Any developer can package their code, integrate it into Agave, and deploy it to Stampede2. Once an app is made publicly available, it is a simple matter of adding it to the iMicrobe "app" table to make it available via the app listing. The user interface to launch an app is dynamically generated at runtime from the same JSON that was used to integrate the app and so requires no additional work on the part of iMicrobe developers to make it available to users.

Figure 6: *The Appetizer is a web interface to assist developers in creating the JSON file needed to describe an app's inputs and parameters to the Agave API. "Inputs" are data files that need to be copied to the compute node to run. "Parameters" are program settings such as integer or strings values that need to be indicated by the end user. The "Advanced" tab allows the app developer to indicate the requirements of the Stampede2 compute nodes such as RAM, CPU, execution queue and time. The "JSON" tab allows the user access to the JSON that is generated by the app.*

Conclusions

Understanding complex biological systems require integration of biological (particularly microbial) processes with characteristics associated with the environment. These complex systems can only be understood in context with other datasets and sampling time points; however, compiling data on microbial diversity and function in a consistent manner where the data can be interlinked, accessible in a single platform, and analyzed using high-performance computer architectures remains challenging despite major innovations in the semantic web and cloud-based computer architectures. The iMicrobe architecture we describe here moves away from a standard data repository approach to a model where data are housed in diverse data repositories and integrated as needed. We use the CyVerse Agave API to retrieve and compute on diverse microbiome datasets that are potentially massive, requiring more disk space and computing power than the average microbial ecologist would have. Further, iMicrobe offers developers a framework for deploying tools to compute on these data using XSEDE HPC resources at Stampede2. By crowdsourcing app development, we enable the community to integrate novel tools that are timely and relevant to their research. We also encourage the development of dynamic documentation at protocols.io. The iMicrobe platform allows users to

manage their data through the complete data lifecycle. Provenance tracking of both primary data, derived files, and analyses are tracked in CyVerse by keeping all files in the analysis directory, along with data products and a log file to maintain information about the job and parameters that were run. By combining all these features, we believe iMicrobe presents a capable platform for large-scale data searching and analysis for the microbiome science community.

Availability of supporting source code and requirements

- Project name: iMicrobe
- Project home page: https://imicrobe.us
- Documentation: https://hurwitzlab.gitbook.io/imicrobe/
- Source code: https://github.com/hurwitzlab/elm-imicrobe-spa, https://github.com/hurwitzlab/node-imicrobe
- Operating system(s): e.g. Platform independent
- Programming language: NA
- Other requirements: CyVerse user account (free)
- License: e.g., MIT

Availability of Supporting Data

Data further supporting this work including snapshots of our code are openly available in the GigaScience repositiory, GigaDB [49].

Abbreviations

API: Application Programming Interface

CAMERA: Community Cyberinfrastructure for Advanced Microbial Ecology Research and Analysis

CI: Cyberinfrastructure

CPU: Central Processing Unit

DOE: Department of Energy

EBI: European Bioinformatics Institute

ENA: European Nucleotide Archive

FAIR: Findable Accessible Interoperable Reusable

FTP: File Transfer Protocol

HPC: High-Performance Computer

HTTP: HyperText Transfer Protocol

IMG: Integrated Microbial Genomes

JS: JavaScript

JSON: JavaScript Object Notation

MS: Mass Spectometry

QC: Quality Control

SDK: Software Development Kit

TACC: Texas Advanced Computing Center

WGS: Whole Genome Shotgun

iRODS: Integrated Rule-Oriented Data System

rRNA: ribosomal ribonucleic acid

Ethics approval and consent to participate

Not applicable.

Consent for publication

Not applicable.

Competing interests

The authors declare that they have no competing interests.

Funding

Initial funding for iMicrobe was provided by the Gordon and Betty Moore Foundation's Marine Microbial Initiative grant #4491. Further development was supported by the National Science

Foundation grant #1639588 and the Simons Foundation’s SCOPE project (Simons Collaboration on Ocean Processes and Ecology).

Authors' contributions

Supervision, Funding acquisition: BH

Conceptualization, Writing: BH, KYC

Software, Project administration: KYC, MB, JL, IC

Data curation: KYC, AP

Validation: JH, AP, EWC

Acknowledgements

We would like to thank Jon Kaye at the Gordon Betty Moore Foundation; our sysadmin, Adam Michel, at UA; Lenny Teytelman and Alexie Stoliartchouk at protocols.io; Nirav Merchant and Ramona Walls at CyVerse; and Matt Vaughn and John Fonner at TACC. This work used the Extreme Science and Engineering Discovery Environment (XSEDE), which is supported by National Science Foundation grant number ACI-1548562.

Table 1. *Current list of available apps in iMicrobe.*

**App Name**	**Purpose**
16s_cluster-0.0.1u2	Cluster 16S sequences [34]
c-microbial-map-0.0.1u1	Visualize geographic distribution of 16S sequences in the

	ocean [50]
centrifuge-1.0.4u1	Short-read taxonomic classification [51]
centrifuge-bubble-0.0.5u1	Visualization of Centrifuge analysis [52]
ClusterGenomes-1.1.3u2	Clusters genomes based on all-verses-all alignments
DIAMOND-0.9.10u1	Fast read alignment of DNA or proteins [53]
fizkin-0.0.3u1	Pairwise sample comparison via kmers [54]
FragGeneScan-1.30.0u1	Short-read ORF prediction [55]
graftm-0.11.1u3	Rapid community profiles from metagenomes [56]
imicrobe-demultiplexer-0.0.1u1	Demultiplexing pipeline for single and paired-end data [57]
imicrobe-megahit-0.0.2u1	Metagenomics read assembler [58]
imicrobe-prokka-0.0.2u1	Prokaryotic genome annotation [59]
imicrobe-soapdenovo2-0.0.3u1	Short-read assembler [60]
libra-1.0	Pairwise sample comparison via kmers [61]
MArVD-1.0.0u1	Metagenomic Archaeal Virus Detector [62]
mash-all-vs-all-0.0.5u1	Pairwise sample comparison via Mash [63]
MetaGeneAnnotator-1.1.0u1	Prokaryotic and phage gene prediction [64]
ohana-blast-0.0.9u2	BLAST search to Ohana gene catalog [65]
prodigal-2.6.3u3	Gene prediction [66]
Prokka-1.12.0u2	Prokaryotic genome annotation [59]
puma-0.3.0u1	Annotation of HPV genomes [67]
Read2RefMapper-1.1.0u2	Filtering coverage of BAM files to a reference dataset [68]
sra-fastq-dump-0.0.1u1	Save sequences from SRA in CyVerse DataStore [69]
trim-galore-0.4.5u1	Quality control tool for trimming reads [70]
Trimmomatic-0.36.0u2	Quality control tool for trimming reads [71]
uproc_dna-1.2.0u3	Protein sequence classification [72]
vContact-0.1.60u2	Viral Contig Automatic Cluster Taxonomy [73]

vContact_PCs-0.1.60u2	Viral Contig Automatic Cluster Taxonomy [73]
WIsH-Build-1.0.0u2	Identify bacterial hosts from metagenomic data [74]
WIsH-Predict-1.0.0u2	Identify bacterial hosts from metagenomic data [74]

Table 2: *Comparison of metagenomic platforms' cyberinfrastructure capabilities.*

	KBase	MGnify	MG-RAST	IMG/M	QIITA	iMicrobe
Create apps	✓					✓
Run apps at will	✓		✓		✓	✓
Upload private data	✓		✓	✓*	✓	✓
Share private data						✓
Search public data	✓	✓		✓	✓	✓

* for users of JGI sequencing services

Table 3: *Comparison of metagenomics platforms' app capabilities.*

		KBase	MGnify	MG-RAST	IMG/M	QIITA	iMicrobe
General	QC	✓	✓	✓	✓	✓	✓
Genomics	Assembly	✓					✓
	Gene calling	✓					✓
	Gene annotation	✓					✓
	Metabolic modeling	✓					
	Sequence analysis	✓					✓

	Comparative genomics	✓					
Metagenomics (assembly-based analysis)	Assembly	✓			✓		✓
	Gene calling			✓	✓		✓
	Gene annotation			✓	✓		✓
	Taxonomic classification of contigs			✓	✓		✓
	Protein clustering			✓			✓
	Read mapping to contigs or other reference	✓					
Metagenomics (read-based analysis)	Read taxonomic classification	✓	✓	✓			✓
	Read ORF prediction		✓	✓			✓
	Read functional annotation		✓	✓			✓
	Read clustering						✓
Amplicon	OTUs & taxonomic lineage		✓	✓	✓	✓	✓

References

1. Youens-Clark K, Bomhoff M, Hurwitz BL. iMicrobe [Internet]. Available from: https://imicrobe.us

2. Wetterstrand KA. DNA Sequencing Costs: Data from the NHGRI Genome Sequencing Program (GSP) [Internet]. Available from: https://www.genome.gov/27541954/dna-sequencing-costs-data/

3. Towns J, Cockerill T, Dahan M, Foster I, Gaither K, Grimshaw A, et al. XSEDE: Accelerating Scientific Discovery. Computing in Science Engineering. 2014;16:62–74.

4. Texas Advanced Computing Center (TACC), The University of Texas at Austin.

5. Dooley R, Brandt SR, Fonner J. The Agave Platform: An Open, Science-as-a-Service Platform for Digital Science. Proceedings of the Practice and Experience on Advanced Research Computing. ACM; 2018. p. 28.

6. Fielding R. Representational state transfer. Architectural Styles and the Design of Netowork-based Software Architecture. 2000;76–85.

7. Dooley R. Agave API [Internet]. Available from: http://agaveapi.co/

8. Merchant N, Lyons E, Goff S, Vaughn M, Ware D, Micklos D, et al. The iPlant Collaborative: Cyberinfrastructure for Enabling Data to Discovery for the Life Sciences. PLoS Biol. 2016;14:e1002342.

9. CyVerse Data Store [Internet]. Available from: https://www.cyverse.org/data-store

10. Seshadri R, Kravitz SA, Smarr L, Gilna P, Frazier M. CAMERA: a community resource for metagenomics. PLoS Biol. 2007;5:e75.

11. Youens-Clark K, Bomhoff M, Hurwitz BL. iMicrobe FTP [Internet]. Available from: ftp.imicrobe.us

12. Rajasekar A, Moore R, Hou C-Y, Lee CA, Marciano R, de Torcy A, et al. iRODS Primer: Integrated Rule-Oriented Data System. Synthesis Lectures on Information Concepts, Retrieval, and Services. Morgan & Claypool Publishers; 2010;2:1–143.

13. CyVerse Data Commons [Internet]. Available from: http://datacommons.cyverse.org/

14. Vaughn M, Carson J. CyVerse Software Development Kit [Internet]. Available from: https://github.com/cyverse/cyverse-sdk

15. Kurtzer GM, Sochat V, Bauer MW. Singularity: Scientific containers for mobility of compute. PLoS One. 2017;12:e0177459.

16. Hammer-Lahav DE, Hardt D. The oauth2. 0 authorization protocol. 2011. Technical report, IETF Internet Draft. 2011;

17. CyVerse Data Policy [Internet]. Available from: https://www.cyverse.org/data-policy

18. Publishing Data Through The Data Commons [Internet]. Available from: https://wiki.cyverse.org/wiki/display/DC/Publishing+Data+through+the+Data+Commons

19. Youens-Clark K, Bomhoff M. iMicrobe Samples Metadata Search [Internet]. Available from: https://www.imicrobe.us/#/samples

20. Wilkinson MD, Dumontier M, Aalbersberg IJJ, Appleton G, Axton M, Baak A, et al. The FAIR Guiding Principles for scientific data management and stewardship. Sci Data. 2016;3:160018.

21. Merkel D. Docker: lightweight Linux containers for consistent development and deployment. Linux J. Belltown Media; 2014;2014:2.

22. Youens-Clark K, Bomhoff M. iMicrobe Apps [Internet]. Available from:

https://www.imicrobe.us/#/apps

23. Mangul S, Martin LS, Eskin E, Blekhman R. Improving the usability and archival stability of bioinformatics software. Genome Biol. 2019;20:47.

24. Teytelman L, Stoliartchouk A, Kindler L, Hurwitz BL. Protocols.io: Virtual Communities for Protocol Development and Discussion. PLoS Biol. 2016;14:e1002538.

25. Hurwitz BL, Ponsero AJ, Youens-Clark K, Bomhoff M. Protocols.io iMicrobe Group [Internet]. Available from: http://www.protocols.io/groups/imicrobe

26. Hurwitz BL, Ponsero A, Youens-Clark K, Bomhoff M. iMicrobe Documentation [Internet]. Available from: https://hurwitzlab.gitbook.io/imicrobe/

27. Arkin AP, Stevens RL, Cottingham RW, Maslov S, Henry CS, Dehal P, et al. The DOE Systems Biology Knowledgebase (KBase) [Internet]. bioRxiv. 2016 [cited 2019 Apr 1]. p. 096354. Available from: https://www.biorxiv.org/content/10.1101/096354v1.abstract

28. MGnify [Internet]. Available from: https://www.ebi.ac.uk/metagenomics

29. MGnify Pipelines [Internet]. Available from: https://www.ebi.ac.uk/metagenomics/pipelines/

30. Wilke A, Bischof J, Gerlach W, Glass E, Harrison T, Keegan KP, et al. The MG-RAST metagenomics database and portal in 2015. Nucleic Acids Res. 2016;44:D590–4.

31. Meyer F. MG-RAST API [Internet]. Available from: http://api.metagenomics.anl.gov/api.html

32. Markowitz VM, Chen I-MA, Palaniappan K, Chu K, Szeto E, Pillay M, et al. IMG 4 version of the integrated microbial genomes comparative analysis system. Nucleic Acids Res. 2014;42:D560–7.

33. Gonzalez A, Navas-Molina JA, Kosciolek T, McDonald D, Vázquez-Baeza Y, Ackermann G, et al. Qiita: rapid, web-enabled microbiome meta-analysis. Nat Methods. 2018;15:796–8.

34. Miller M. 16s_cluster-0.0.1u2 [Internet]. Available from: https://github.com/hurwitzlab/imicrobe-16SrDNA-OTU-Clustering

35. Czaplicki E. Elm: Concurrent frp for functional guis. Senior thesis, Harvard University. 2012;

36. Jones SP. Haskell 98 Language and Libraries: The Revised Report. Cambridge University Press; 2003.

37. Surhone LM, Tennoe MT, Henssonow SF. Node.Js. Mauritius: Betascript Publishing; 2010.

38. MySQL Relational Database Management System [Internet]. Available from: https://dev.mysql.com/

39. Chodorow K, Dirolf M. MongoDB: The Definitive Guide. O'Reilly Media; 2010.

40. Crockford D. JSON RFC [Internet]. Available from: https://www.ietf.org/rfc/rfc4627.txt

41. Youens-Clark K, Bomhoff M. iMicrobe Projects [Internet]. Available from: https://www.imicrobe.us/#/projects

42. Youens-Clark K, Bomhoff M. iMicrobe Projects API [Internet]. Available from: https://www.imicrobe.us/api/v1/projects

43. MySQL FULLTEXT Indexes [Internet]. Available from: https://dev.mysql.com/doc/refman/5.5/en/fulltext-search.html

44. Docker Hub [Internet]. Available from: https://hub.docker.com/

45. da Veiga Leprevost F, Grüning BA, Alves Aflitos S, Röst HL, Uszkoreit J, Barsnes H, et al. BioContainers: an open-source and community-driven framework for software standardization. Bioinformatics. academic.oup.com; 2017;33:2580–2.

46. Youens-Clark K. Trim Galore Biocontainer/Singularity [Internet]. Available from:

https://github.com/hurwitzlab/trim-galore

47. Dooley R. Agave App Inputs and Parameters [Internet]. Available from: http://developer.agaveapi.co/#inputs-and-parameters

48. Youens-Clark K. The Appetizer [Internet]. Available from: http://appetizer.hurwitzlab.org/

49. Youens-Clark K; Bomhoff M; Ponsero A; Wood-Charlson EM; Lynch J; Choi I; Hartman JH; Hurwitz BL: Supporting data for "iMicrobe: Tools and data-driven discovery platform for the microbiome sciences" GigaScience Database. 2019. http://dx.doi.org/10.5524/100611

50. Youens-Clark K, McNichol J. C-Microbial-Map [Internet]. Available from: https://github.com/simonscmap/c-microbial-map

51. Kim D, Song L, Breitwieser FP, Salzberg SL. Centrifuge: rapid and sensitive classification of metagenomic sequences. Genome Res. 2016;26:1721–9.

52. Thornton J, Youens-Clark K. Centrifuge Bubble Plot [Internet]. Available from: https://github.com/hurwitzlab/centrifuge-bubble

53. Buchfink B, Xie C, Huson DH. Fast and sensitive protein alignment using DIAMOND. Nat Methods. 2015;12:59–60.

54. Youens-Clark K, Hurwitz B. Fizkin [Internet]. Available from: https://github.com/hurwitzlab/fizkin

55. Rho M, Tang H, Ye Y. FragGeneScan: predicting genes in short and error-prone reads. Nucleic Acids Res. 2010;38:e191.

56. Boyd JA, Woodcroft BJ, Tyson GW. GraftM: a tool for scalable, phylogenetically informed classification of genes within metagenomes. Nucleic Acids Res. 2018;46:e59.

57. Miller M. iMicrobe Demultiplexer [Internet]. Available from:

https://github.com/mattmiller899/demultiplex_app

58. Li D, Liu C-M, Luo R, Sadakane K, Lam T-W. MEGAHIT: an ultra-fast single-node solution for large and complex metagenomics assembly via succinct de Bruijn graph. Bioinformatics. 2015;31:1674–6.

59. Seemann T. Prokka: rapid prokaryotic genome annotation. Bioinformatics. 2014;30:2068–9.

60. Xie Y, Wu G, Tang J, Luo R, Patterson J, Liu S, et al. SOAPdenovo-Trans: de novo transcriptome assembly with short RNA-Seq reads. Bioinformatics. 2014;30:1660–6.

61. Choi I, Ponsero AJ, Bomhoff M, Youens-Clark K, Hartman JH, Hurwitz BL. Libra: scalable k-mer-based tool for massive all-vs-all metagenome comparisons. Gigascience [Internet]. 2019;8. Available from: http://dx.doi.org/10.1093/gigascience/giy165

62. Vik DR, Roux S, Brum JR, Bolduc B, Emerson JB, Padilla CC, et al. Putative archaeal viruses from the mesopelagic ocean. PeerJ. 2017;5:e3428.

63. Ondov BD, Treangen TJ, Melsted P, Mallonee AB, Bergman NH, Koren S, et al. Mash: fast genome and metagenome distance estimation using MinHash. Genome Biol. 2016;17:132.

64. Noguchi H, Taniguchi T, Itoh T. MetaGeneAnnotator: detecting species-specific patterns of ribosomal binding site for precise gene prediction in anonymous prokaryotic and phage genomes. DNA Res. 2008;15:387–96.

65. Mende DR, Bryant JA, Aylward FO, Eppley JM. Environmental drivers of a microbial genomic transition zone in the ocean's interior. Nature [Internet]. nature.com; 2017; Available from: https://www.nature.com/articles/s41564-017-0008-3

66. Hyatt D, Chen G-L, Locascio PF, Land ML, Larimer FW, Hauser LJ. Prodigal: prokaryotic gene recognition and translation initiation site identification. BMC Bioinformatics. 2010;11:119.

67. Van Doorslaer K, Pace J, Youens-Clark K, Freeman C. Puma [Internet]. Available from: https://github.com/KVD-lab/puma

68. Bolduc B, Roux S. Ref2ReadMapper [Internet]. Available from: https://bitbucket.org/bolduc/docker-read2refmapper/

69. Bomhoff M. SRA FASTQ Dump [Internet]. Available from: https://github.com/hurwitzlab/fastq-dump

70. Krueger F. Trim galore. A wrapper tool around Cutadapt and FastQC to consistently apply quality and adapter trimming to FastQ files. 2015;

71. Bolger AM, Lohse M, Usadel B. Trimmomatic: a flexible trimmer for Illumina sequence data. Bioinformatics. 2014;30:2114–20.

72. Meinicke P. UProC: tools for ultra-fast protein domain classification. Bioinformatics. 2015;31:1382–8.

73. Bolduc B, Jang HB, Doulcier G, You Z-Q, Roux S, Sullivan MB. vConTACT: an iVirus tool to classify double-stranded DNA viruses that infect Archaea and Bacteria. PeerJ. 2017;5:e3243.

74. Galiez C, Siebert M, Enault F, Vincent J, Söding J. WIsH: who is the host? Predicting prokaryotic hosts from metagenomic phage contigs. Bioinformatics. 2017;33:3113–4.

Click here to access/download;Figure;figure_1.png

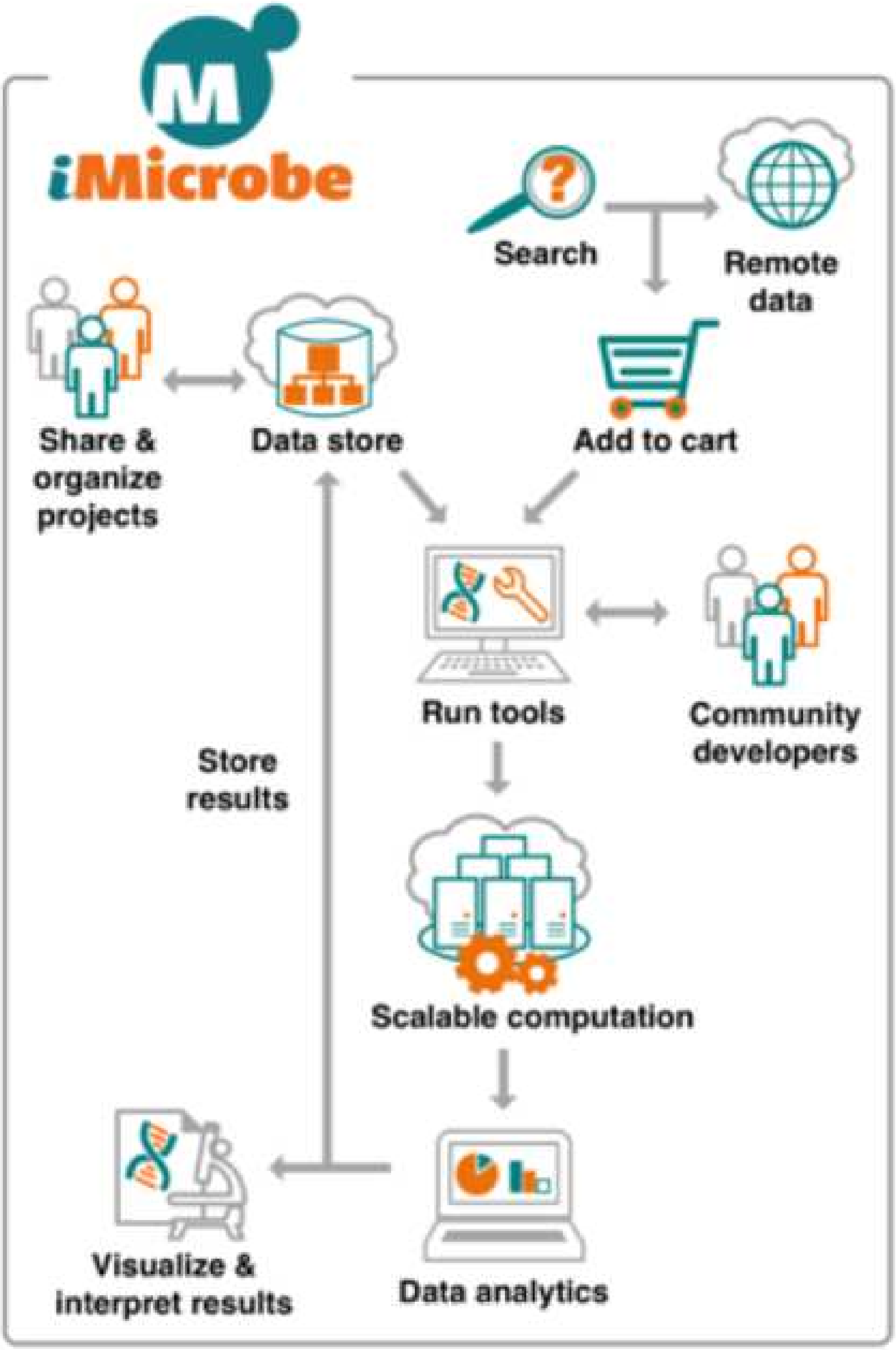

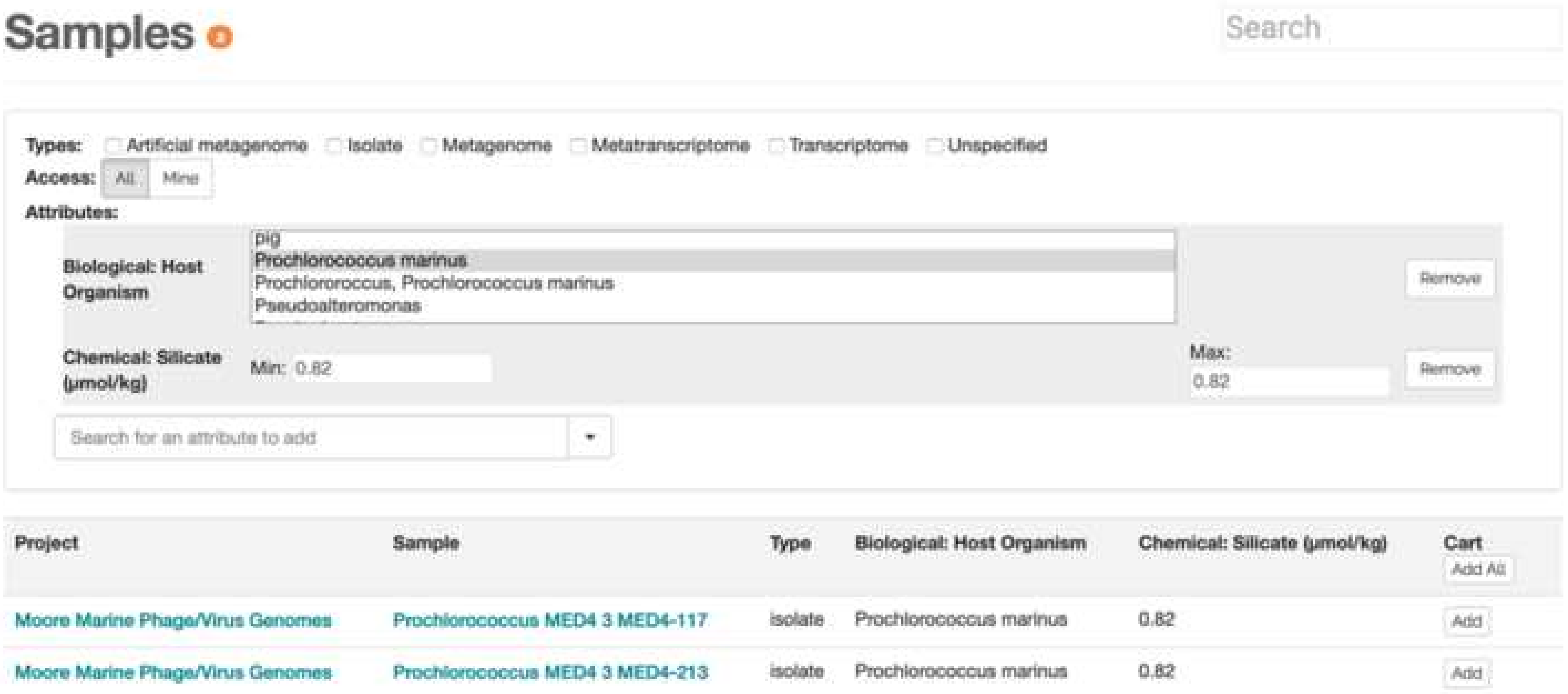
Samples
Search
Types: Artificial metagenome Isolate Metagenome Metatranscriptome Transcriptome Unspecified
Access: All Mine
Attributes:
Biological: Host Organism
pig
Prochlorococcus marinus
Prochlororoccus, Prochlorococcus marinus
Pseudoalteromonas
Remove
Chemical: Silicate (µmol/kg)
Min: 0.82
Max: 0.82
Remove
Search for an attribute to add
Project
Sample
Type
Biological: Host Organism
Chemical: Silicate (µmol/kg)
Cart
Add All
Moore Marine Phage/Virus Genomes
Prochlorococcus MED4 3 MED4-117
isolate
Prochlorococcus marinus
0.82
Add
Moore Marine Phage/Virus Genomes
Prochlorococcus MED4 3 MED4-213
isolate
Prochlorococcus marinus
0.82
Add

Click here to access/download;Figure;figure_3.png

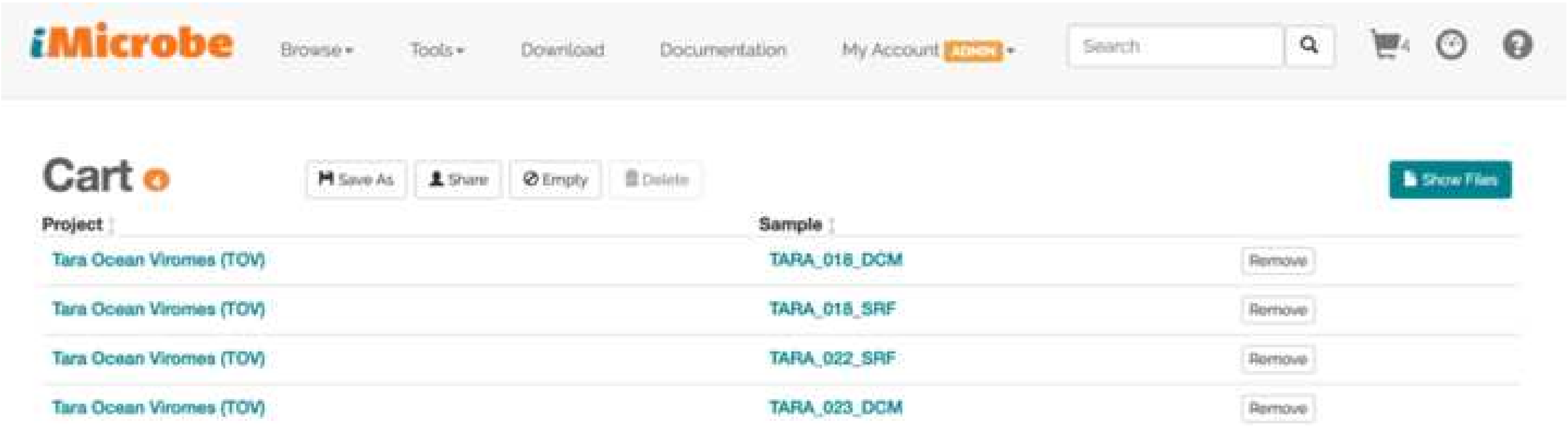

Click here to access/download;Figure;figure_4.png

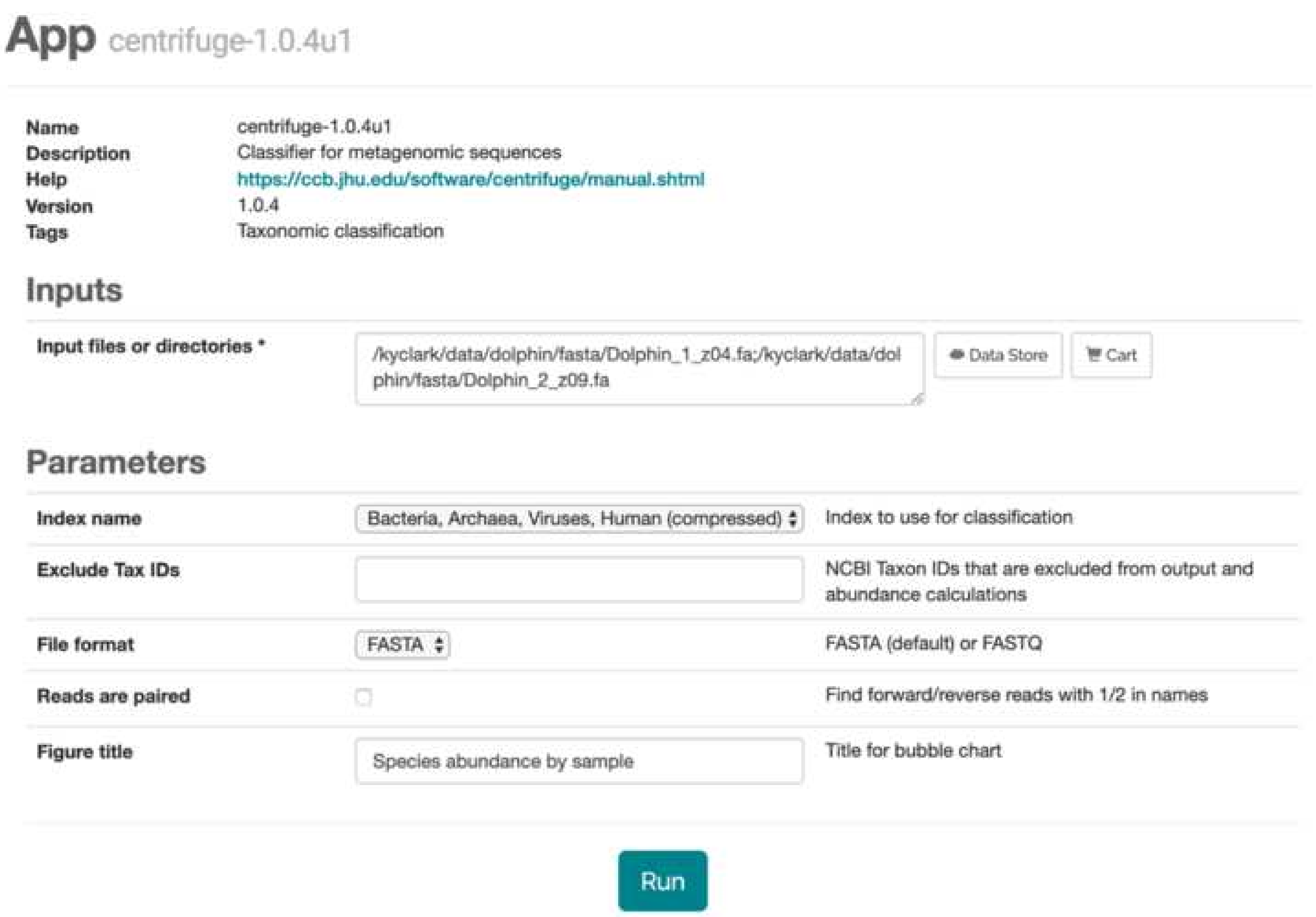

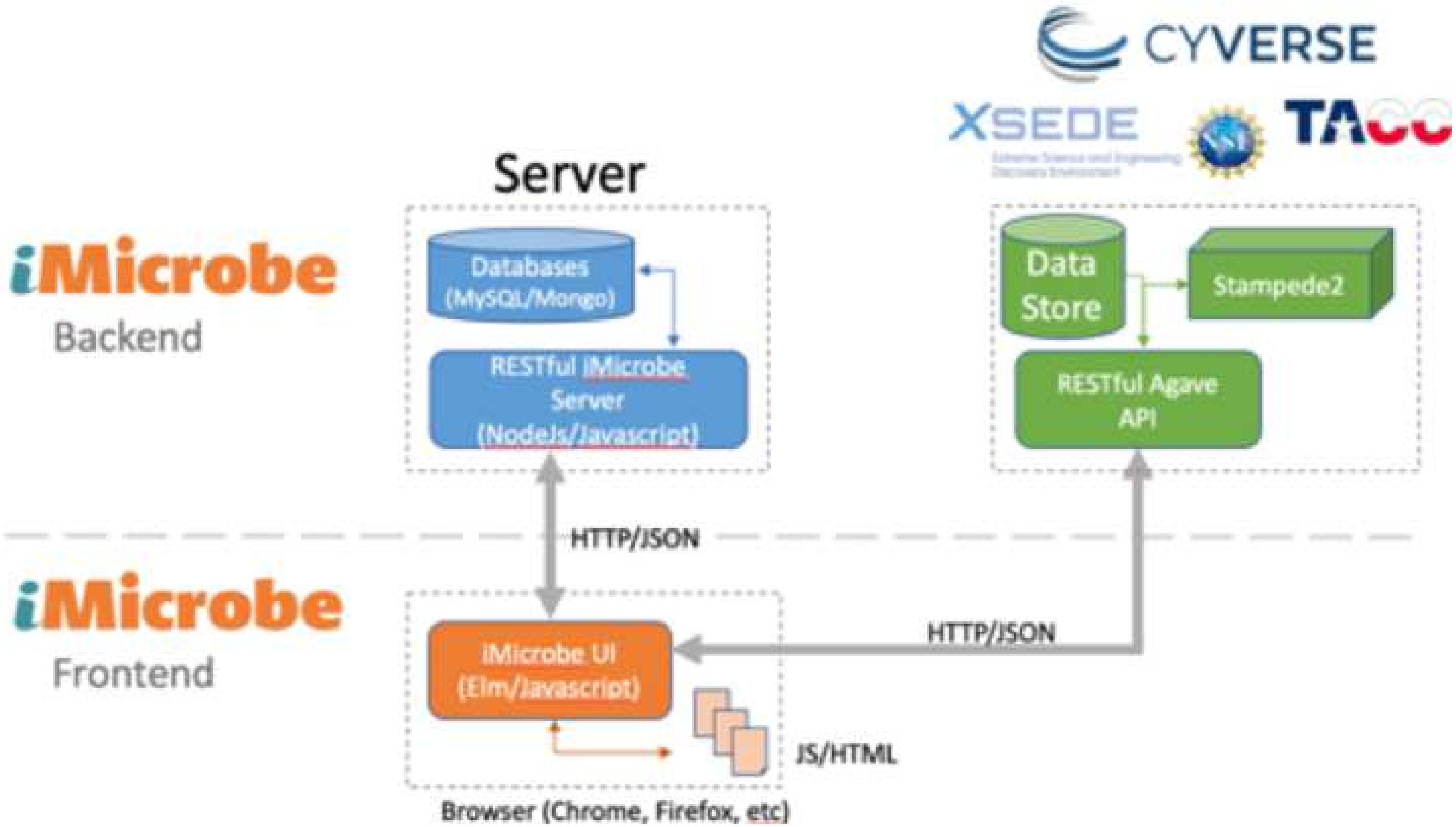
CYVERSE
XSEDE
TACC
Server
iMicrobe
Backend
Databases
(MySQL/Mongo)
RESTful iMicrobe
Server
(NodeJs/Javascript)
Data
Store
Stampede2
RESTful Agave
API
HTTP/JSON
iMicrobe
Frontend
iMicrobe UI
(Elm/Javascript)
HTTP/JSON
JS/HTML
Browser (Chrome, Firefox, etc)

Click here to access/download;Figure;figure_6.png

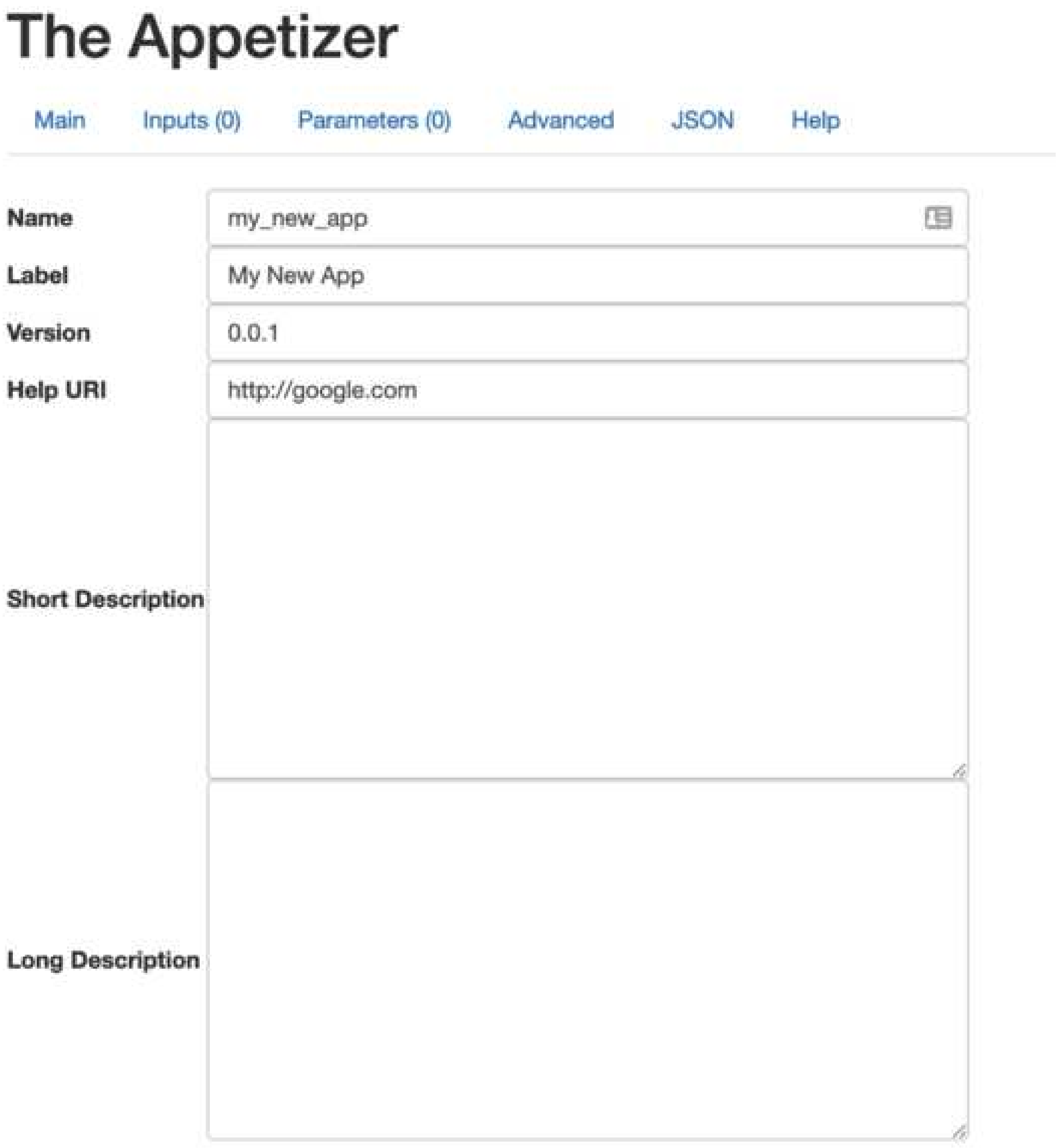

response to reviews

Click here to access/download
Supplementary Material
response_to_reviewers.docx
