# Supplementary material for: iMicrobe: Tools and data-driven discovery platform for the microbiome sciences
Source: Gigascience. 2019 Jul 9;8(7):giz083. doi: 10.1093/gigascience/giz083 (PMC6615980; doi:10.1093/gigascience/giz083)
Supplement: giz083_Response_to_Reviewer_Comments_Original_Submission [file giz083_response_to_reviewer_comments_original_submission.pdf]

Author's Response To Reviewer Comments

Close

Response to Reviewers

Reviewer #1:
This manuscript describes iMicrobe, which is an online discovery and analysis platform for microbiome data. The manuscript is well written and easy to follow, and the resource is described in detail. The iMicrobe platform addresses an important need, allowing researchers to analyse their data and other publicly available datasets without the overhead of designing and implementing their own analysis workflows or pipelines. While other platforms already exist for this purpose, they tend to be relatively inflexible in terms of workflows provided, whereas iMicrobe offers a range of user-selectable analysis components. Provision of an additional microbiome analysis platform is also very positive for the research community, as microbiome research is a broad and complex field requiring a diversity of approaches, with no single tool or platform able to address every analysis need.

The iMicrobe platform is commendable in a number of its aims. These include i) emphasis on contextual sample metadata, ii) recording of analysis provenance, iii) containerisation of tools / analysis components to make them interoperable and to run on diverse compute environments, and iv) in providing users with access to super-compute resources by leveraging CyVerse and XSEDE. Nevertheless, I have some reservations about some of the finer details described in the manuscript that should be clarified or resolved prior to publication.

Response: We are grateful for your careful review and kind words about the iMicrobe platform. The points you make on the functionality and utility are exactly in line with what we hope to provide to the community.

In terms of data sets that can be analysed using the resource, there does not seem to be any mechanism for users to submit their sequencing data into a publicly available archive. Access to this data is vital so that data sets can be re-used, re-analysed and results compared for the benefit of the scientific community. Several of the major microbiome analysis platforms, including QIITA, MGnify and MG-RAST already offer this facility, brokering data into ENA. The iMicrobe developers should consider adding this option.

Response: We agree with the reviewer about the importance of being able to not only manage data in iMicrobe, but also submit to data repositories. We recently became a data broker for ENA and added functionality to the iMicrobe Data Dashboard to submit data to the European Nucleotide Archive (ENA). We added the description of this new feature in the manuscript as such:

“Additionally, iMicrobe is a data broker for the European Nucleotide Archive (ENA). Users can upload sequence files, enter project/sample metadata, and publish to ENA, all within the iMicrobe interface. iMicrobe validates the data/metadata and suggests additions or corrections but does not enforce any standards. This feature is available on request. A future version of the site will include this and conform to community standards.”

On page 6, the authors highlight the ability to search data sets by metadata. This is a very useful facility. However, the web site would benefit greatly from pagination. At present, searching for a widely populated attribute such as 'location:depth' returns a single page with thousands of samples listed, which is unwieldy.

Response: We appreciate and agree with this suggestion. We added this suggestion to our list of planned site improvements. We also added in a sentence in the manuscript describing the feedback button on our site. All feedback is sent to a Basecamp project site, where bug fixes and new feature suggestions are discussed and prioritized.

Also on page 6, the authors state 'Samples are described in detail', but it is not clear where these

descriptions are coming from. Are these provided by the original data submitters or are they curated by iMicrobe? Do projects comply with the MIxS community standards in terms of associated information; are the annotation fields and units used in line with the checklists for metagenome and metatranscriptome data?

Response: We thank the reviewer for raising these important questions. Many of the datasets included in iMicrobe to date were inherited from the CAMERA project. Unfortunately, these historical data were not curated by CAMERA or the original data providers and do not conform to MiXS standards. We are currently working on a project called Planet Microbe that will provide a new model for interoperable and standardized data for large-scale ocean expeditions (e.g. Tara, Malaspina, GOS, and Ocean Sampling Day). Moreover, we will add tools that allow the user to conform to these community-defined standards and submit data to ENA based on these standards. The idea is to harmonize historical datasets, and then provide a utility for users to continue to submit "gold-standard" data to promote data interoperability and re-use. This resource will be available for the ocean science community, but will be a model for other communities going forward.

We added the following sentence under the Metadata Search Section:

"Data sets included in the metadata search were inherited from the CAMERA project and were not thoroughly curated by CAMERA or the original data providers and do not conform to current community standards. As such, discrepancies may exist and users can contact original data providers and the iMicrobe developers via the website to address problems."

Furthermore, who is the owner of the annotation (iMicrobe or the original data submitter?) and who should be contacted or is authorised to make changes or updates (for example, to correct an attribute that has been misannotated)?

Response: The data and the annotations belong to the original data submitter. To date, discrepancies have been reported directly to iMicrobe via our feedback page (https://www.imicrobe.us/#/contact), and our developers have corrected the mistakes. We clarified this point in the paper in the above point about data inherited from CAMERA.

On page 10, the authors state that 'any data set with a web link are accessible and computable in iMicrobe'. How realistic is this statement and does it need some kind of caveat for excessively large data sets? For example, if a user was to provide a link to the Tara Oceans project, which represents many Tb of sequencing effort, how feasible would it be to assemble and annotate the complete dataset, and what would be the kind of timescale?

Response: We thank the reviewer for this very important comment. Indeed, the Agave API can only retrieve reasonably sized data sets available over the Internet, and may fail for very large scale datasets such as the complete Tara ocean dataset. Moreover, iMicrobe uses resources available via TACC where compute jobs are typically limited to 24-48 hours. This is indeed a limit in term of computation power available through iMicrobe. We updated the main text to make this point clearer and added warning messages to the interface to inform the user on this limitation as such:

"Reasonably sized data sets that are available via a web-link (FTP or HTTP) are accessible and computable in iMicrobe if the analysis can be completed within a standard 24-48-hour window."

On pages 12-15, the authors compare iMicrobe to other major publicly available microbiome analysis platforms and highlight the modular and configurable nature of the analyses available in iMicrobe. However, the manuscript does not mention the volume of data available via these resources (eg, 64,000 publicly available datasets in MG-RAST, 160,000 in MGnify, 250,000 in QIITA) and how this compares to iMicrobe (~6k). I think this is important to mention for fairness, since one of the benefits of using a standardised analysis pipeline is that it allows high-throughput, thus there tends to be a configurable analysis component/throughput trade-off. The authors might also want to discuss analysis of different kingdoms by the different platforms (for example, IMG/MG provide viral taxonomic analysis; MGnify, QIITE and MG-RAST do not). The statement 'Metadata searches in MGnify include temperature, depth and a few other fields', is also incorrect, as MGnify has over a hundred searchable metadata fields, which can be returned via the API (although metadata completeness depends on user submission and hence varies between projects).

Response: We thank the reviewer for these very important points. We agree with the reviewer on these

points and updated the main text to provide a fairer comparison of the platforms:

“”

In Table 1, the authors list the apps that are currently available in iMicrobe, and this list will hopefully grow over time. The authors should comment on why (at least some) of these components were chosen - are they the best in breed in terms or performance, or were they chosen for more prosaic reasons, such as ease of containerisation?

Response: We thank the reviewer for this question. We do hope to increase the number of apps available through iMicrobe, especially by involving the research community for the inclusion of their most used tools and pipelines and by leveraging a tool we developed called The Appetizer.
The initial App development focused on tools and pipelines for read-based analytics, or community-specific tools or pipelines (such as the iVirus apps) that were not available in other platforms. We updated the manuscript to include this description:

“The current selection of apps provide additional read-based analytics not currently available on other platforms and specialized pipelines from collaborators. Future development will also include community-defined pipelines that are made available by major data repositories, such as ENA, to allow users to harmonize their data with other public data sources in a consistent manner.”

Along these lines, I have concerns relating to some of the Centrifuge taxonomic analysis results available on the site, in that many samples appear to have Homo sapiens as the most abundant source of sequencing reads. In some cases, such as human host-associated samples, this could represent host contamination and may therefore be reasonable. However, reads classified as human are also the majority in various environmental samples (Viral Stromatolite Metagenome; Microbial community gene expression across a productivity gradient of the Amazon River plume; Botany Bay Metagenomes, etc). Is this really contamination or is it a spurious result from Centrifuge?

Response: Thank you for this comment. We have also noticed this issue with the Centrifuge taxonomic annotation tool and are currently investigating it. For another paper, we are developing apps (and containers) for all k-mer based taxonomic classifiers and comparing them side-by-side using synthetic and biological datasets of known composition. This work will help to define issues in classification that can be propagated back to the site to correct any issues. The paper is currently in review and edits are due back by the end of this month, so this work will be complete in the next month.

Finally, for a number of datasets (for example Canine fecal metagenome, CAM_S_443), I was not able to see any downloadable files, protein analysis results or taxonomic analysis results on the site. Some kind of QC visualisation should be added to the site to help understand what lies behind this type of result (for example did the sequences not pass validation or did the analysis pipeline fail for some reason?)

We are aware of issues with the CAMERA data sets on iMicrobe. There is currently an effort by the National Microbiome Data Collaborative (NMDC) to curate these among many other historical data sets using community-defined standards. We hope to leverage these improved data sets on our site in the future.

Reviewer #2:
The authors present what appears to be a potent platform for analysis of microbiome sequencing data. The platform offers a some free compute and storage resources that appear to be enough for smaller studies. Within the platform, comparisons can be made to already publicly available data, or additional data provided by the user. Flexibility appears high, and the authors appear to expect most of the workflow design will be made by the end-users.

Online analysis platforms similar to iMicrobe have existed for several years, often using similar selling points, which typically include e.g. scaling to large analyses utilizing powerful compute resources, using or integrating with large datasets produced by others, etc. Some of the recurrent issues with online analysis platforms include the need to upload substantial amounts of data to an online server, the reliability and expected lifetime of the platform, and often the limited amount of flexibility in analyses. The first item is hard to work around, so I won't spend any time on that. It appears iMicrobe deals with the latter by essentially offering a web-based interface to standard command-line bioinformatics applications packaged in Singularity containers, leaving the workflow planning and execution to the end-

users. I feel there is too little space in the article devoted to explaining how to connect the output from one app to the input of another and generally how to implement complete analysis workflows on the platform. There are some references to protocols.io, but I think it would benefit the article to have some more detail about this topic included.

Response: We thank the reviewer for a careful review of our work. We agree with the reviewer and think that the implementation of more complex workflows where the output of one app could be connected to a second app is an important feature. This constitutes our main future direction for the platform. We added this point in the discussion as such:

“Currently workflows are implemented inside an app. Future development plans include chaining apps together to create ad hoc workflows.”

The idea of an online analysis platform where data can be easily compared to other data from other groups is appealing, but for it to be truly useful it requires a large and active user base and reliable long-term funding. It is unclear to me from reading the paper what I can expect as an end-user about the long-term accessibility to the platform and data. Is there a designated place where end-users should direct questions, discussions, etc (other than maybe protocols.io)?

Response: We completely agree with the reviewer. Long-term usage and availability of the platform is one of the most critical aspect of this kind of project. Given lessons learned from the CAMERA project, we are highly aware of the consequences of the long-term availability of the ressources. We have therefore made several steps to allow reusability of this project by other communities. Specifically, we are currently working on submitting all CAMERA datasets to ENA that are not currently available in NCBI/ENA. Moreover, the tools developed for iMicrobe are available as containers for the community to reuse either by building locally from the recipes available in the public Hurwitz Lab GitHub repos (https://github.com/hurwitzlab), downloading them from ftp.imicrobe.us, or using directly on the Stampede2 cluster. We updated manuscript to make this critical point clear as follows:

“To ensure the long-term availability of the apps, we provide recipes to build the Singularity containers in the GitHub repository for each tool as well as the containers themselves on the iMicrobe FTP site and direct access to the containers in a public directory on the Stampede2 cluster.”

We agree with the reviewer that a place for user discussion and comments is needed. To this end, we added an additional sentence in the manuscript to say that this feature is available in the iMicrobe group at protocols.io.

“Through protocols.io, scientists can also create and share their own protocols and/or groups in the microbiome sciences as well as engage in discussions.”

To me, one of the weaker points of the article is the motivation for the existence of another online analysis platform. Thus, the section with comparisons to other online platforms for microbiome dataset analysis is very important. The idea of a comparison section is so that users familiar with any of the mentioned services can compare the basic similarities/differences more easily. However, I feel the descriptions are fairly thin and doesn't provide enough detail to produce any meaningful comparisons. Maybe the information be summarized in a feature table that compares all tools simultaneously rather than just providing plain text paragraphs doing pairwise comparisons to iMicrobe?

Response: We thank the reviewer for this comment, we agree with these points and added a two tables summarizing the comparison as such:

Table 2: Comparison of metagenomic platforms’ cyberinfrastructure capabilities.
KBase MGnify MG-RAST IMG/M QIITA iMicrobe
Create apps ✓ ✓
Run apps at will ✓ ✓ ✓ ✓
Upload private data ✓ ✓ ✓* ✓ ✓
Share private data ✓
Search public data ✓ ✓ ✓ ✓ ✓
* for users of JGI sequencing services

Table 3: Comparison of metagenomics platforms’ app capabilities.

KBase MGnify MG-RAST IMG/M QIITA iMicrobe
General QC ✓ ✓ ✓ ✓ ✓ ✓
Genomics Assembly ✓ ✓
Gene calling ✓ ✓
Gene annotation ✓ ✓
Metabolic modeling ✓
Sequence analysis ✓ ✓
Comparative genomics ✓
Metagenomics (assembly-based analysis) Assembly ✓ ✓ ✓
Gene calling ✓ ✓ ✓
Gene annotation ✓ ✓ ✓
Taxonomic classification of contigs ✓ ✓ ✓
Protein clustering ✓ ✓
Read mapping to contigs or other reference ✓
Metagenomics (read-based analysis) Read taxonomic classification ✓ ✓ ✓ ✓
Read ORF prediction ✓ ✓ ✓
Read functional annotation ✓ ✓ ✓
Read clustering ✓
Amplicon OTUs & taxonomic lineage ✓ ✓ ✓ ✓ ✓

The mention of limitations of QIIME2 to 16S data in the section on QIITA appears incorrect. As far as I can tell from QIIME2's homepage there exists plugins (MetaPhlAn2 and SHOGUN) to also analyze shotgun data.

Response: We thank the reviewer for this correction, we updated the text accordingly as such:

“QIITA [33] allows users to upload and analyze maker gene data sets (16S rRNA, ITS, and 18S rRNA), metagenomic, and metabolomic datasets using QIIME2 and Global Natural Product Social Molecular Networking (GNPS) tools. Using these tools, users can examine the taxonomic composition of marker gene and whole genome shotgun (WGS) metagenomic datasets, and perform automated molecular network analysis and crowdsourced MS/MS spectrum curation for metabolomic datasets. However, QIIME2 does not currently support upstream analyses for WGS datasets or analyses beyond taxonomic annotation and analysis. iMicorbe currently hosts a 16S rRNA pipeline and clustering tool [34], in addition to many other general purpose tools for QC, trimming, read assembly, pairwise sequence alignment, and gene functional annotation, among others.”

I would like to see a more in depth explanation of the long-term plan to handle app versioning. In a couple of years, there will have been a multitude of different tools and a large amount of newer versions of the same tools. How will that be managed in the interface? For now, it appears it is mostly a long list of all available applications, which will most likely become cumbersome or hard to browse over time.

Response: We agree with the reviewer, this is a very important point. We started implementing terms to the tools describing their operation, data input and output according to the EDAM ontology of bioinformatic operations. In the future, when the number of apps grow larger, this aims will allow the user to retrieve apps by their function, input data type or output. We added this text to the manuscript:

“Currently users can find tools by using standardized tags or app names and descriptions. As the app lists grows, we plan to offer additional ways to organize and search for apps, including by application versions.”

Close
