# Supplementary material for: iMicrobe: Tools and data-driven discovery platform for the microbiome sciences
Source: Gigascience. 2019 Jul 9;8(7):giz083. doi: 10.1093/gigascience/giz083 (PMC6615980; doi:10.1093/gigascience/giz083)
Supplement: giz083_Reviewer_1_Report_Original_Submission — Alex Mitchell -- 4/21/2019 Reviewed [file giz083_reviewer_1_report_original_submission.pdf]

Reviewer Report

Title: iMicrobe: Tools and data-driven discovery platform for the microbiome sciences

Version: Original Submission **Date:** 4/21/2019

Reviewer name: Alex Mitchell

Reviewer Comments to Author:

This manuscript describes iMicrobe, which is an online discovery and analysis platform for microbiome data. The manuscript is well written and easy to follow, and the resource is described in detail. The iMicrobe platform addresses an important need, allowing researchers to analyse their data and other publicly available datasets without the overhead of designing and implementing their own analysis workflows or pipelines. While other platforms already exist for this purpose, they tend to be relatively inflexible in terms of workflows provided, whereas iMicrobe offers a range of user-selectable analysis components. Provision of an additional microbiome analysis platform is also very positive for the research community, as microbiome research is a broad and complex field requiring a diversity of approaches, with no single tool or platform able to address every analysis need.
The iMicrobe platform is commendable in a number of its aims. These include i) emphasis on contextual sample metadata, ii) recording of analysis provenance, iii) containerisation of tools / analysis components to make them interoperable and to run on diverse compute environments, and iv) in providing users with access to super-compute resources by leveraging CyVerse and XSEDE. Nevertheless, I have some reservations about some of the finer details described in the manuscript that should be clarified or resolved prior to publication.
In terms of data sets that can be analysed using the resource, there does not seem to be any mechanism for users to submit their sequencing data into a publicly available archive. Access to this data is vital so that data sets can be re-used, re-analysed and results compared for the benefit of the scientific community. Several of the major microbiome analysis platforms, including QIITA, MGnify and MG-RAST already offer this facility, brokering data into ENA. The iMicrobe developers should consider adding this option.
On page 6, the authors highlight the ability to search data sets by metadata. This is a very useful facility. However, the web site would benefit greatly from pagination. At present, searching for a widely populated attribute such as 'location:depth' returns a single page with thousands of samples listed, which is unwieldy.
Also on page 6, the authors state 'Samples are described in detail', but it is not clear where these descriptions are coming from. Are these provided by the original data submitters or are they curated by iMicrobe? Do projects comply with the MIxS community standards in terms of associated information; are the annotation fields and units used in line with the checklists for metagenome and metatranscriptome data? Furthermore, who is the owner of the annotation (iMicrobe or the original data submitter?) and who should be contacted or is authorised to make changes or updates (for example, to correct an attribute that has been misannotated)?
On page 10, the authors state that 'any data set with a web link are accessible and computable in

iMicrobe'. How realistic is this statement and does it need some kind of caveat for excessively large data sets? For example, if a user was to provide a link to the Tara Oceans project, which represents many Tb of sequencing effort, how feasible would it be to assemble and annotate the complete dataset, and what would be the kind of timescale?
On pages 12-15, the authors compare iMicrobe to other major publicly available microbiome analysis platforms and highlight the modular and configurable nature of the analyses available in iMicrobe. However, the manuscript does not mention the volume of data available via these resources (eg, 64,000 publicly available datasets in MG-RAST, 160,000 in MGnify, 250,000 in QIITA) and how this compares to iMicrobe (~6k). I think this is important to mention for fairness, since one of the benefits of using a standardised analysis pipeline is that it allows high-throughput, thus there tends to be a configurable analysis component/throughput trade-off. The authors might also want to discuss analysis of different kingdoms by the different platforms (for example, IMG/MG provide viral taxonomic analysis; MGnify, QIITE and MG-RAST do not). The statement 'Metadata searches in MGnify include temperature, depth and a few other fields', is also incorrect, as MGnify has over a hundred searchable metadata fields, which can be returned via the API (although metadata completeness depends on user submission and hence varies between projects).
In Table 1, the authors list the apps that are currently available in iMicrobe, and this list will hopefully grow over time. The authors should comment on why (at least some) of these components were chosen - are they the best in breed in terms or performance, or were they chosen for more prosaic reasons, such as ease of containerisation? Along these lines, I have concerns relating to some of the Centrifuge taxonomic analysis results available on the site, in that many samples appear to have Homo sapiens as the most abundant source of sequencing reads. In some cases, such as human host-associated samples, this could represent host contamination and may therefore be reasonable. However, reads classified as human are also the majority in various environmental samples (Viral Stromatolite Metagenome; Microbial community gene expression across a productivity gradient of the Amazon River plume; Botany Bay Metagenomes, etc). Is this really contamination or is it a spurious result from Centrifuge?
Finally, for a number of datasets (for example Canine fecal metagenome, CAM_S_443), I was not able to see any downloadable files, protein analysis results or taxonomic analysis results on the site. Some kind of QC visualisation should be added to the site to help understand what lies behind this type of result (for example did the sequences not pass validation or did the analysis pipeline fail for some reason?)

Level of Interest

Please indicate how interesting you found the manuscript: Choose an item.

Quality of Written English

Please indicate the quality of language in the manuscript: Choose an item.

Declaration of Competing Interests

Please complete a declaration of competing interests, considering the following questions:

- Have you in the past five years received reimbursements, fees, funding, or salary from an organisation that may in any way gain or lose financially from the publication of this manuscript, either now or in the future?
- Do you hold any stocks or shares in an organisation that may in any way gain or lose financially from the publication of this manuscript, either now or in the future?
- Do you hold or are you currently applying for any patents relating to the content of the manuscript?
- Have you received reimbursements, fees, funding, or salary from an organization that holds or has applied for patents relating to the content of the manuscript?
- Do you have any other financial competing interests?
- Do you have any non-financial competing interests in relation to this paper?

If you can answer no to all of the above, write 'I declare that I have no competing interests' below. If your reply is yes to any, please give details below.

I declare that I have no competing interests

I agree to the open peer review policy of the journal. I understand that my name will be included on my report to the authors and, if the manuscript is accepted for publication, my named report including any attachments I upload will be posted on the website along with the authors' responses. I agree for my report to be made available under an Open Access Creative Commons CC-BY license (http://creativecommons.org/licenses/by/4.0/). I understand that any comments which I do not wish to be included in my named report can be included as confidential comments to the editors, which will not be published.

I agree to the open peer review policy of the journal

To further support our reviewers, we have joined with Publons, where you can gain additional credit to further highlight your hard work (see: https://publons.com/journal/530/gigascience). On publication of this paper, your review will be automatically added to Publons, you can then choose whether or not to claim your Publons credit. I understand this statement.

Yes Choose an item.
