# Supplementary material for: iMicrobe: Tools and data-driven discovery platform for the microbiome sciences
Source: Gigascience. 2019 Jul 9;8(7):giz083. doi: 10.1093/gigascience/giz083 (PMC6615980; doi:10.1093/gigascience/giz083)
Supplement: giz083_Reviewer_2_Report_Original_Submission — Fredrik Boulund -- 5/27/2019 Reviewed [file giz083_reviewer_2_report_original_submission.pdf]

Reviewer Report

Title: iMicrobe: Tools and data-driven discovery platform for the microbiome sciences

Version: Original Submission **Date:** 5/27/2019

Reviewer name: Fredrik Boulund

Reviewer Comments to Author:

The authors present what appears to be a potent platform for analysis of microbiome sequencing data. The platform offers a some free compute and storage resources that appear to be enough for smaller studies. Within the platform, comparisons can be made to already publicly available data, or additional data provided by the user. Flexibility appears high, and the authors appear to expect most of the workflow design will be made by the end-users.

Online analysis platforms similar to iMicrobe have existed for several years, often using similar selling points, which typically include e.g. scaling to large analyses utilizing powerful compute resources, using or integrating with large datasets produced by others, etc. Some of the recurrent issues with online analysis platforms include the need to upload substantial amounts of data to an online server, the reliability and expected lifetime of the platform, and often the limited amount of flexibility in analyses. The first item is hard to work around, so I won't spend any time on that. It appears iMicrobe deals with the latter by essentially offering a web-based interface to standard command-line bioinformatics applications packaged in Singularity containers, leaving the workflow planning and execution to the end-users. I feel there is too little space in the article devoted to explaining how to connect the output from one app to the input of another and generally how to implement complete analysis workflows on the platform. There are some references to protocols.io, but I think it would benefit the article to have some more detail about this topic included.

The idea of an online analysis platform where data can be easily compared to other data from other groups is appealing, but for it to be truly useful it requires a large and active user base and reliable long-term funding. It is unclear to me from reading the paper what I can expect as an end-user about the long-term accessibility to the platform and data. Is there a designated place where end-users should direct questions, discussions, etc (other than maybe protocols.io)?

To me, one of the weaker points of the article is the motivation for the existence of another online analysis platform. Thus, the section with comparisons to other online platforms for microbiome dataset analysis is very important. The idea of a comparison section is so that users familiar with any of the mentioned services can compare the basic similarities/differences more easily. However, I feel the descriptions are fairly thin and doesn't provide enough detail to produce any meaningful comparisons. Maybe the information be summarized in a feature table that compares all tools simultaneously rather than just providing plain text paragraphs doing pairwise comparisons to iMicrobe?

The mention of limitations of QIIME2 to 16S data in the section on QIITA appears incorrect. As far as I can tell from QIIME2's homepage there exists plugins (MetaPhlAn2 and SHOGUN) to also analyze shotgun data.

I would like to see a more in depth explanation of the long-term plan to handle app versioning. In a

couple of years, there will have been a multitude of different tools and a large amount of newer versions of the same tools. How will that be managed in the interface? For now, it appears it is mostly a long list of all available applications, which will most likely become cumbersome or hard to browse over time.

Level of Interest

Please indicate how interesting you found the manuscript: Choose an item.

Quality of Written English

Please indicate the quality of language in the manuscript: Choose an item.

Declaration of Competing Interests

Please complete a declaration of competing interests, considering the following questions:

- Have you in the past five years received reimbursements, fees, funding, or salary from an organisation that may in any way gain or lose financially from the publication of this manuscript, either now or in the future?
- Do you hold any stocks or shares in an organisation that may in any way gain or lose financially from the publication of this manuscript, either now or in the future?
- Do you hold or are you currently applying for any patents relating to the content of the manuscript?
- Have you received reimbursements, fees, funding, or salary from an organization that holds or has applied for patents relating to the content of the manuscript?
- Do you have any other financial competing interests?
- Do you have any non-financial competing interests in relation to this paper?

If you can answer no to all of the above, write 'I declare that I have no competing interests' below. If your reply is yes to any, please give details below.

I declare that I have no competing interests

I agree to the open peer review policy of the journal. I understand that my name will be included on my report to the authors and, if the manuscript is accepted for publication, my named report including any attachments I upload will be posted on the website along with the authors' responses. I agree for my report to be made available under an Open Access Creative Commons CC-BY license (http://creativecommons.org/licenses/by/4.0/). I understand that any comments which I do not wish to be included in my named report can be included as confidential comments to the editors, which will not be published.

I agree to the open peer review policy of the journal

To further support our reviewers, we have joined with Publons, where you can gain additional credit to further highlight your hard work (see: https://publons.com/journal/530/gigascience). On publication of this paper, your review will be automatically added to Publons, you can then choose whether or not to claim your Publons credit. I understand this statement.

Yes Choose an item.
